# Birth rate decline in the later phase of the COVID-19 pandemic: the role of policy interventions, vaccination programmes, and economic uncertainty

**DOI:** 10.1093/hropen/hoae052

**Published:** 2024-09-10

**Authors:** Maria Winkler-Dworak, Kryštof Zeman, Tomáš Sobotka

**Affiliations:** Vienna Institute of Demography, Austrian Academy of Sciences, Vienna, Austria; Vienna Institute of Demography, Austrian Academy of Sciences, Vienna, Austria; Vienna Institute of Demography, Austrian Academy of Sciences, Vienna, Austria

**Keywords:** births, total fertility rate, COVID-19 pandemic, policy responses, inflation, vaccination, economic uncertainty, unemployment rate, higher-income countries

## Abstract

**STUDY QUESTION:**

What are the factors influencing the decline in the birth rates observed in higher-income countries in the later phase of the COVID-19 pandemic?

**SUMMARY ANSWER:**

Our results suggest that economic uncertainty, non-pharmaceutical policy interventions, and the first wave of the population-wide vaccination campaign were associated with the decline in birth rates during 2022.

**WHAT IS KNOWN ALREADY:**

During the COVID-19 pandemic, birth rates in most higher-income countries first briefly declined and then shortly recovered, showing no common trends afterwards until early 2022, when they unexpectedly dropped.

**STUDY DESIGN, SIZE, DURATION:**

This study uses population-wide data on monthly total fertility rates (TFRs) adjusted for seasonality and calendar effects provided in the Human Fertility Database (HFD). Births taking place between November 2020 and October 2022 correspond to conceptions occurring between February 2020 and January 2022, i.e. after the onset of the pandemic but prior to the Russian invasion of Ukraine. The data cover 26 countries, including 21 countries in Europe, the USA, Canada, Israel, Japan, and the Republic of Korea.

**PARTICIPANTS/MATERIALS, SETTING, METHODS:**

First, we provided a descriptive analysis of the monthly changes in the TFR. Second, we employed linear fixed effects regression models to estimate the association of explanatory factors with the observed seasonally adjusted TFRs. Our analysis considered three broader sets of explanatory factors: economic uncertainty, policy interventions restricting mobility and social activities outside the home, and the progression of vaccination programmes.

**MAIN RESULTS AND THE ROLE OF CHANCE:**

We found that birth trends during the COVID-19 pandemic were associated with economic uncertainty, as measured by increased inflation (*P* < 0.001), whereas unemployment did not show any link to births during the pandemic (*P* = 0.677). The stringency of pandemic policy interventions was linked to a postponement of births, but only in countries with lower institutional trust and only in the early phase of the pandemic (*P* = 0.003). In countries with higher trust, stricter containment measures were positively associated with birth rates, both for conceptions in the first year of the pandemic (*P* = 0.019) and, albeit only weakly significant, for conceptions later in the pandemic (*P* = 0.057). Furthermore, we found a negative association between the share of the population having received the first dose of the COVID-19 vaccination and TFRs (*P* < 0.001), whereas the share of the population having completed the primary vaccination course (usually consisting of two doses) was linked to a recovery of birth rates (*P* < 0.001).

**LARGE SCALE DATA:**

N/A.

**LIMITATIONS, REASONS FOR CAUTION:**

Our research is restricted to higher-income countries with relatively strong social support policies provided by the government as well as wide access to modern contraception. Our data did not allow analyses of birth trends by key characteristics, such as age, birth order, and social status.

**WIDER IMPLICATIONS OF THE FINDINGS:**

This is the first multi-country study of the drivers of birth trends in the later phase of the COVID-19 pandemic. In the past, periods following epidemics and health crises were typically associated with a recovery in births. In contrast, our results show that the gradual phasing out of pandemic containment measures, allowing increased mobility and a return to more normal work and social life, contributed to declining birth rates in some countries. In addition, our analysis indicates that some women avoided pregnancy until completion of the primary vaccination protocol.

**STUDY FUNDING/COMPETING INTEREST(S):**

This study did not use any external funding. The authors acknowledge funding from their home institution, the Vienna Institute of Demography of the Austrian Academy of Sciences, and from the Open-Access Fund of the Austrian Academy of Sciences. For the purpose of open access, the authors have applied a CC BY public copyright licence to any Author Accepted Manuscript versions arising from this submission. All authors declare that they have no conflicts of interest.

WHAT DOES THIS MEAN FOR PATIENTS? Shocks, crises, and health emergencies often affect reproductive plans because people are reluctant to make major decisions during uncertain times. The recent COVID-19 pandemic is one such shock, causing temporary ups and downs in birth rates. We studied monthly changes in birth rates across 26 higher-income countries, most of which had low total fertility rates (i.e. a low number of children per woman) before the pandemic. We focus especially on birth trends from late 2021 to October 2022. In this period, birth rates dropped in most of these countries. These births were conceived from early 2021 until the start of the Russian invasion in Ukraine in February 2022, which is yet another shock that may have affected reproductive plans in some countries.

Why did birth rates drop in most of the analysed countries in early 2022? We find three reasons for the fall in birth rates. First, rising inflation led to declining birth rates. Second, because of pandemic containment measures in the preceding year, people worked from home and spent more time with their families, resulting in a better work–life balance for some couples. In some countries, this led to more births occurring in 2021; however, easing out of the pandemic interventions afterwards then put a break on this temporary positive effect on births. Third, the COVID-19 vaccination campaign was also linked with a temporary drop in birth rates. Some women might have wanted to be fully vaccinated before getting pregnant in order to protect their and their child’s health. We show that when more people were eventually fully vaccinated, birth rates increased again. However, this increase in birth rates was hidden by the two other factors that lowered birth rates in most countries.

Our results show that the vaccine did not harm women’s ability to have children. Rather, we found that women were temporarily avoiding or postponing pregnancies. Birth rates already started dropping when most of the women of childbearing age could not yet get vaccinated, but rose when most of the population was fully vaccinated. Also, the number of births fell by a different magnitude in the various countries: Portugal even saw more births during the peak vaccination period. If the vaccine caused infertility, the birth rates would have dropped sharply in all the countries at the same time, when the vaccination take-up was peaking. The findings underscore the importance of providing early, clear, accurate, and consistent information about the safety of the vaccine to women and couples of reproductive age.

## Introduction

The COVID-19 pandemic of 2020–2022 contributed to distinct swings in birth rates. The initial shock was linked in most countries to a short-term decrease in the number of births around December 2020 to January 2021, followed by an equally brief recovery around March 2021 and a more differentiated development in the subsequent months that varied across countries (e.g. OECD, 2021a,b; Gray *et al.*, 2022; Nisén *et al.*, 2022; Pomar *et al.*, 2022; Bailey *et al.*, 2023; Fallesen and Cozzani, 2023; Gietel-Basten and Chen, 2023; Kearney and Levine, 2023; Lappegård *et al.*, 2023; Plach *et al.*, 2023; Sobotka *et al.*, 2023). Some countries, including the Nordic countries, the Netherlands, Switzerland, Germany, Israel, and the USA, even experienced a minor ‘baby boom’ during the second pandemic year, 2021. On balance, the changes in birth rates were smaller and more heterogenous than initially expected when considering the unprecedented impact of COVID-19 and the government responses to it on everyday lives, the labour market, and social relations (Settersten *et al.*, 2020; Mayer, 2022; Nitsche and Wilde, 2024).

However, in the later phase of the pandemic, many higher-income countries experienced yet another shift in birth rates, an unexpected and robust downturn from early 2022 (Le Vu *et al.*, 2023; Sobotka *et al.*, 2023; Bujard and Andersson, 2024; Jasilioniene *et al.*, 2024) that often persisted or even accelerated later in 2022 and 2023 (HFD, 2024). This trend appeared surprising in light of past evidence on the impact of health, political, and economic shocks, and upheavals on birth trends. Such shocks typically bring about a decline or postponement of births (e.g. Caldwell, 2004; Sobotka *et al.*, 2011; Marteleto *et al.*, 2020; Boberg-Fazlic *et al.*, 2021), but subsequently, as societies recover from these shocks, births tend to recover as well (Mamelund, 2004; Boberg-Fazlic *et al.*, 2021; De Geyter, 2022).

What could be the drivers of the unexpected decrease in births starting around January 2022? Going back nine months in time, to account for a typical length of pregnancy, we arrive in spring 2021, a time that can be considered a gradual ‘return to normality’. The disruptive impact of the pandemic diminished markedly in most countries. Lockdowns and social distancing measures were gradually phased out and were eventually lifted in 2022. As a consequence, people’s mobility and social contacts increased. Moreover, economic and labour market indicators had largely recovered from the initial pandemic shock. This return to normality was also achieved thanks to the COVID-19 vaccination programme, which was eventually becoming accessible to the whole population around mid-2021 in most countries. At the same time, inflation gradually increased in 2021 due to the increase in oil prices and the global supply chain crisis (supply and demand shocks brought on by the pandemic). Later, surging energy prices and wider uncertainty in the wake of the Russian invasion of Ukraine started fully affecting birth trends in late 2022. As a result of this renewed decline in birth rates, some countries reported record-low period total fertility rates (TFRs) in 2022 and 2023 (e.g. Nordic Statistic Database, 2023).

While birth trends during the early stages of the COVID-19 pandemic have been extensively researched (e.g. Pomar *et al.*, 2022; Bailey *et al.*, 2023; Kearney and Levine, 2023; Plach *et al.*, 2023; Sobotka *et al.*, 2023), the majority of studies have focused on single countries and conceptions in the first year of the pandemic. The most recent birth declines and their drivers are not yet well documented and understood. Our study focuses on three sets of factors that can be empirically assessed and that may explain the unexpected drop in births starting around January 2022: (i) economic uncertainty, (ii) phasing out of policy interventions restricting mobility and social contacts outside of the immediate family, and (iii) the role of the vaccination programme.

There is ample evidence of the link between economic factors and birth trends, where unemployment, inflation and economic uncertainty mostly depress birth rates (e.g. Sobotka *et al.*, 2011; Goldstein *et al.*, 2013; Schneider, 2015; Comolli, 2017; Matysiak *et al.*, 2021; Neels et al., 2024). Economic uncertainty, including job disruptions and worries about unemployment and income loss, jumped in the initial stage of the pandemic (OECD, 2021a,b). In response, some women decided to delay or forego motherhood (Matsushima *et al.*, 2023). Starting in the (later part of) spring of 2020, governments invested massively in job retention and income support schemes to mitigate the negative impact of the pandemic on the labour market, household income, and economic output. In most of the higher-income countries, the unemployment rate returned to pre-pandemic levels by early 2021 and continued to decline until mid-2022. However, inflation rates started rising from mid-2021 during the period of economic recovery, which may have counteracted the positive response on birth rates from economic recovery (OECD, 2023b).

To combat the spread of the virus and to support the economy, governments issued non-pharmaceutical policy interventions (NPIs), such as work and school closures, travel restrictions, lockdowns, as well as income support and special subsidies for businesses affected by these interventions. These containment measures led to major disruptions in social and family life (OECD, 2021a,b; Mayer, 2022). They also resulted in increased stress and relationship struggles (Bellani and Vignoli, 2022), factors that are negatively associated with an intention to become pregnant (Manning *et al.*, 2022; Tasneem *et al.*, 2023), especially when the containment measures were stricter and lasted longer. It has been shown (Pomar *et al.*, 2022) that the decline in birth rates during the first COVID-19 wave was strongly associated with the duration of the lockdowns in 21 higher-income countries. At the same time, economic support cushioned financial pressure and economic uncertainty. Plach *et al.* (2023) found that stricter containment measures led to a postponement of births, while economic support policies were positively associated with birth rates, but only in countries with low pre-pandemic social support policies (measured by public expenditures on family, health, and unemployment support). The authors argued that pre-pandemic support policies broadly reflected the level of social trust, which might mitigate the negative consequences of pandemic-related uncertainty on birth trends. However, their analysis was mostly based on data pertaining to the early phase of the pandemic. As the pandemic progressed, individuals developed coping strategies (Toffolutti *et al.*, 2022), which may have altered the link between policy responses and birth rates in the later part of the pandemic.

With limited opportunities for leisure, recreation, and socialization, people started spending much more time at home with their partners and families. Working from home and saving commuting time to the workplace contributed to a better work–life balance. The opportunity costs of having a child declined. Under favourable circumstances, especially when feeling economically and socially secure, some couples might have rethought their priorities and decided to have a(nother) child or, more likely, have their next planned child earlier (Berrington *et al.*, 2022; Neyer *et al.*, 2022; Lappegård *et al.*, 2023). Bujard and Andersson (2024) term this a ‘cocooning effect’; less poetically, Kearney and Levine (2023) referred to people who were ‘stuck home’ with their romantic partners. As lockdowns and other restrictions gradually eased, especially after the COVID-19 vaccine became widely available in March–June 2021, people resumed work-related, leisure, and socializing activities outside the home. In countries where COVID-19 containment measures resulted in fewer births, their ending would be expected to boost birth rates. In contrast, in countries where the ‘cocooning effect’ contributed to rising birth rates, the end of pandemic-related restrictions would be expected to depress birth rates.

The decline in birth rates in early 2022 could also be linked to the first COVID-19 vaccination campaign in 2021. A large body of literature shows that COVID-19 vaccination does not lead to infertility problems among women or men or to increased adverse pregnancy outcomes (Chen *et al.*, 2021; Zaçe *et al.*, 2022; Wang *et al.*, 2023); vaccination also does not increase the risk of miscarriage among pregnant women (Rimmer *et al.*, 2023). However, it is likely that vaccination affected birth rates indirectly: couples might have decided to temporarily put pregnancy plans on hold during the vaccination program to reduce any potential harm to their foetus’s health (Bujard and Andersson, 2024). Such a decision would not be completely irrational; when vaccines were developed and introduced in late 2020, national health organizations and associations were hesitant to recommend vaccination during pregnancy until conclusive evidence was reached that COVID-19 vaccines are perfectly safe for pregnant women (see e.g. UK Government, 2021). Until early 2021, health authorities, including the US Centre for Disease Control and Prevention (CDC), adopted a cautious approach, suggesting that ‘pregnant women may choose to get any of the vaccines and should discuss risks and benefits with their healthcare providers’ (CDC, 2021). In mid-2021, only 22 out of 224 countries or territories recommended and 78 permitted (with qualifications) vaccination of pregnant women (Berman Institute of Bioethics & Center for Immunization Research JHU, 2023). Most of the vaccines available in 2021 required two doses scheduled 3–12 weeks apart (or even longer intervals, especially when the supply of vaccines was still restricted) to complete the full course of vaccination (for a comparison see e.g. Fiolet *et al.*, 2022). Thus, some couples might have postponed their planned pregnancy for several months until finishing the full vaccination course.

In addition to these factors, birth trends might have been affected by the dynamics of the COVID-19 pandemic itself. Periods of higher infection rates and excess mortality might be associated with depressed birth rates due to the worries women may have about becoming infected while pregnant, or to the desire to avoid hospitals and healthcare systems during infection peaks and to avoid possible exposure to COVID-19 during routine check-ups (Berrington *et al.*, 2022). Some studies have shown a negative association between reported COVID-19 infections, deaths, or overall excess deaths and birth rates, especially in the earlier phases of the pandemic (De Geyter *et al.*, 2022; Kearney and Levine, 2023).

The objective of this study is to analyse the monthly birth trends in higher-income countries during the COVID-19 pandemic, with a particular focus on 2022, when birth rates dropped in the majority of countries. To gain a deeper understanding of the factors influencing this decline, the study investigates the role of policy interventions, the vaccination programme, and economic uncertainty in driving the decline of births during the later phase of the pandemic.

## Materials and methods

### Study population and period

This study uses population-wide data on monthly births and TFRs collected in the Short-term Fertility Fluctuations (STFF) data series within the Human Fertility Database (HFD, 2024). The STFF data provide up-to-date information on live births by month of occurrence in selected countries with complete registration of births and available monthly reporting. Their monthly format makes the STFF data especially suitable for studying changes in birth rates that may arise in response to sudden economic, political, or pandemic shocks and changing policies, including the COVID-19 pandemic and the policies enacted to combat the spread of the virus. Our analysis focuses on birth trends from November 2020 to October 2022, covering conceptions occurring from February 2020 to January 2022. This period encompasses the onset of the pandemic but ends before the Russian invasion of Ukraine in February 2022, which might have also affected birth dynamics. The data cover 26 countries, including 21 countries in Europe, the USA, Canada, Israel, Japan, and the Republic of Korea (hereafter called South Korea). Table 1 provides an overview of the data, countries and regions covered. Among the data covered in the STFF data, we did not include Bulgaria, Lithuania, and Russia, due to missing data for some of the explanatory variables.

**Table 1.** Annual total fertility rates in 2019–2022, number of births, population size, and trust in government before the pandemic by country.

	Total fertility rate				Births (thousand)	Population (million)	
Country	2019	2020	2021	2022	2022	2022	Trust
*Northern Europe*							
Denmark	1.70	1.68	1.73	1.55	58.4	5.9	Higher
Finland	1.35	1.37	1.46	1.32	45.0	5.6	Higher
Norway	1.53	1.48	1.55	1.40	51.5	5.5	Higher
Sweden	1.71	1.67	1.67	1.50	104.7	10.5	Higher
*Western Europe*							
Belgium	1.60	1.57	1.60	1.54	113.6	11.6	Lower
France	1.83	1.79	1.80	1.76	686.9	65.5	Lower
Ireland	1.71	1.66	1.72	1.60	54.4	5.0	Lower
Netherlands	1.57	1.55	1.62	1.51	167.5	17.5	Higher
UK	1.64	1.57	1.53	1.50	672.6	66.9	Lower
*German-speaking countries*							
Austria	1.46	1.44	1.48	1.41	82.6	9.0	Lower
Germany	1.54	1.53	1.58	1.47	738.8	82.8	Higher
Switzerland	1.48	1.47	1.52	1.39	82.4	8.7	Higher
*Central-Eastern Europe*							
Czechia	1.75	1.76	1.82	1.68	101.3	10.5	Lower
Hungary	1.49	1.56	1.59	1.55	88.5	9.7	Lower
Latvia	1.61	1.55	1.57	1.46	16.0	1.9	Lower
Poland	1.41	1.38	1.33	1.25	305.1	38.3	Lower
Slovenia	1.61	1.60	1.64	1.57	17.6	2.1	Lower
*Southern Europe*							
Greece	1.34	1.39	1.43	1.31	76.1	10.6	Lower
Italy	1.27	1.25	1.25	1.24	392.6	58.7	Lower
Portugal	1.43	1.41	1.34	1.45	83.7	10.3	Lower
Spain	1.24	1.19	1.19	1.20	330.2	47.2	Lower
*North America*							
Canada	1.47	1.40	1.43	1.36	351.7	38.2	Higher
USA	1.70	1.64	1.66	1.66	3 665.0	332.0	Lower
*East Asia*							
Japan	1.34	1.33	1.30	1.25	770.8	122.3	Lower
South Korea	0.92	0.84	0.81	0.78	249.2	51.2	Lower
*Other regions*							
Israel	3.01	2.90	3.00	2.93	181.2	9.5	Lower

While the analysed data provide complete coverage of births, data for some countries might be affected by late reporting, reporting by month of registration rather than by month of birth, and different rules pertaining to the inclusion or exclusion of births by foreigners, refugees, and asylum seekers residing in a country. Details about the birth data for individual countries are provided in the STFF Metadata document (HFD STFF, 2024).

### Indicators used: monthly total fertility rates

The analysis used monthly series of TFRs adjusted for both calendar and seasonal variation, i.e. the monthly number of births is adjusted for weekday and monthly variations of births, where the adjustment was done separately for each country. The method of estimating the monthly TFRs from the absolute monthly number of births as well as the seasonal adjustment are explained in detail in the STFF Methodological Note (Jdanov *et al.*, 2022).

The TFR is an indicator estimating the number of children that would be born per woman if age-specific fertility rates among women of reproductive age (15–49 years) remained the same indefinitely as in the observed period. Compared with the absolute number of births or crude birth rates, the TFR has the advantage of not being affected by changes in the size of the female population of reproductive age over time and can easily be compared across countries. However, the TFR may be affected by changes in the age of childbearing. When births are shifted to younger or older childbearing ages, the TFR does not properly reflect the ultimate family size among women at the end of their reproductive span (Bongaarts and Feeney, 1998; Sobotka and Lutz, 2011).

### Covariates

Explanatory variables include indicators reflecting economic uncertainty, NPIs, as well as indicators of the vaccination rollout.

For economic uncertainty, we used the seasonally adjusted monthly harmonized unemployment rate (OECD, 2023d) and the monthly consumer price index, with 2015 as the base year (OECD, 2023a). For easier interpretation, we rescaled the consumer price index relative to its value in the year 2019. We also considered other monthly economic indicators such as the consumer confidence index and the economic policy uncertainty index (Baker *et al.*, 2016). However, these indicators were not available for all countries in this study.

We include two NPI indices (the stringency index and the economic support index) from the Oxford COVID-19 Government Response Tracker (Hale *et al.*, 2021). The stringency index is a composite measure of nine policy responses: school closures, workplace closures, cancellation of public events, restrictions on public gatherings, closures of public transport, stay-at-home orders, restrictions on internal movement, international travel controls, and public information campaigns. Each component is normalized to range from 0 to 100, and the index is computed as an average of the nine components. Higher values indicate more stringent measures, with 100 representing the most stringent response. Similarly, the economic support index is calculated as the average of the policy responses on income support as well as on debt relief and contract relief.

Building upon the argument of Plach *et al.* (2023) on the importance of social trust mediating the association between government policies and birth rates, we differentiated countries according to the level of social trust using annual data on trust in government before the pandemic (2010–2019) from OECD (2023c). By employing partitioning around medoids methods (Kaufman and Rousseeuw, 1990), countries were grouped into those with lower and higher trust in government (see Table 1). Using different clustering methods, such as k-Means or hierarchical clustering, did not yield different country clustering for the set of countries used in this study. The resulting clustering gave similar results to the ones based on pre-pandemic social support used by Plach *et al.* (2023), although Canada and Ireland display lower social support but higher trust, and conversely, Austria, France, and Belgium show high social support but lower trust.

Vaccination programmes in most countries were age-graded, with the oldest population being the first to receive the vaccine. Women and men of reproductive age became eligible several months later, usually in the spring or early summer of 2021 (Hale *et al.*, 2021). Israel was the main exception as a ‘forerunner’ country staging an earlier and faster vaccination campaign and having 60% of its population receiving at least one dose by March 2021. Most vaccines required two doses to complete the primary protocol. To account for potential pregnancy postponement during the initial COVID-19 vaccination programme, we used data on both the share of the population that received at least one dose and the share of the population who completed the initial COVID-19 vaccination protocol over time. If some women indeed decided to avoid or postpone pregnancies around the time of their vaccination, the former indicator, representing ongoing vaccination, should have a negative effect on birth rates, while the latter, representing the completion of vaccination, should have a positive effect. The data come from Our World in Data (Mathieu *et al.*, 2020), which collates and processes the up-to-date official vaccination data on a daily or weekly basis for the total population. We used interpolation techniques to derive the mid-month value. For an additional analysis, we used data available for broader age groups and selected the share of the population having received the first dose and completing the primary course of vaccination, respectively, in the prime childbearing age group (25–49 years). These data were only available for 20 out of the 26 countries. Israel, Canada, and Switzerland use different age groups; we therefore selected a narrower age group, specifically 30–39 years, to represent the prime childbearing ages.

### Control variables

In addition, we controlled for the health emergency during the pandemic. We used excess mortality as reported by Our World in Data (Mathieu *et al.*, 2020). Excess mortality is measured using a *p*-score, which corresponds to the relative difference between the reported number of deaths (HMD, 2023) and the projected (based on pre-pandemic trends) number of deaths from all causes (Karlinsky and Kobak, 2021). Data on excess mortality are provided on a monthly or weekly basis, where we converted weekly data into monthly averages. In addition, we included a dummy variable in our statistical analysis for the first wave of the COVID-19 pandemic from February to April 2020, reflecting the high level of uncertainty just after the start of the pandemic.

Supplementary Figure S1 illustrates pre-pandemic and pandemic seasonally adjusted monthly TFRs from November 2018 to December 2022, by country. Furthermore, Supplementary Fig. S2 presents monthly trends in the covariates from January 2019 to January 2022 overlaid on the seasonally adjusted monthly TFR (lagged by nine months), separately for all countries analysed.

### Statistical methods and models

We first analysed birth trends in 26 high-income countries since the onset of the COVID-19 pandemic, documenting the accelerated decline in births in many countries since early 2022. Next, we assessed the correlations of the individual explanatory variables with the seasonally adjusted monthly TFR, shifted by nine months to the approximate time of conception, in each country. Then, we adopted a multivariable approach by estimating a linear fixed effects regression model of the relationship between the TFR and the explanatory variables.

The baseline model included the indicators measuring economic uncertainty (the unemployment rate and the consumer price index), NPIs (the stringency index and the economic support index), vaccination rollout (the cumulative share of the population that received the first dose and that completed the initial vaccination protocol for the vaccination rollout), and controlled for pandemic severity and stage (using excess mortality and a dummy variable for the first COVID-19 wave).

Next, we added the stringency index lagged by one month to allow for later adjustments of birth rates associated with the containment measures. For instance, a negative coefficient of the (non-lagged) stringency index in conjunction with a positive coefficient of the lagged stringency index would suggest that birth rates were negatively associated nine months after restrictions were in place but that births were subsequently partly recovered one month later. Moreover, we examined whether the association between the NPI and TFRs differs across countries depending on the level of social trust and whether these associations have changed over the course of the pandemic.

We estimated the fixed effects regression models by adopting Driscoll and Kraay (1998) standard errors, which are robust to disturbances being heteroskedastic, autocorrelated, and cross-sectional dependent (Hoechle, 2007). In fact, the Breusch–Pagan test rejected the null hypothesis of homoskedasticity. We performed the Woolridge test for serial correlation in panel-data models and the Pesaran’s CD test for testing for cross-sectional dependence. Both tests rejected the absence of autocorrelation and cross-section dependence, respectively. A *P*-value <0.05 was considered to indicate statistical significance. While the data and the descriptive analysis were carried out in R 4.3.2 (R Core Team, Vienna, Austria), Stata 18 (StataCorp, College Station, TX, USA) was used for the regression analysis.

## Results

**Figure 1.**
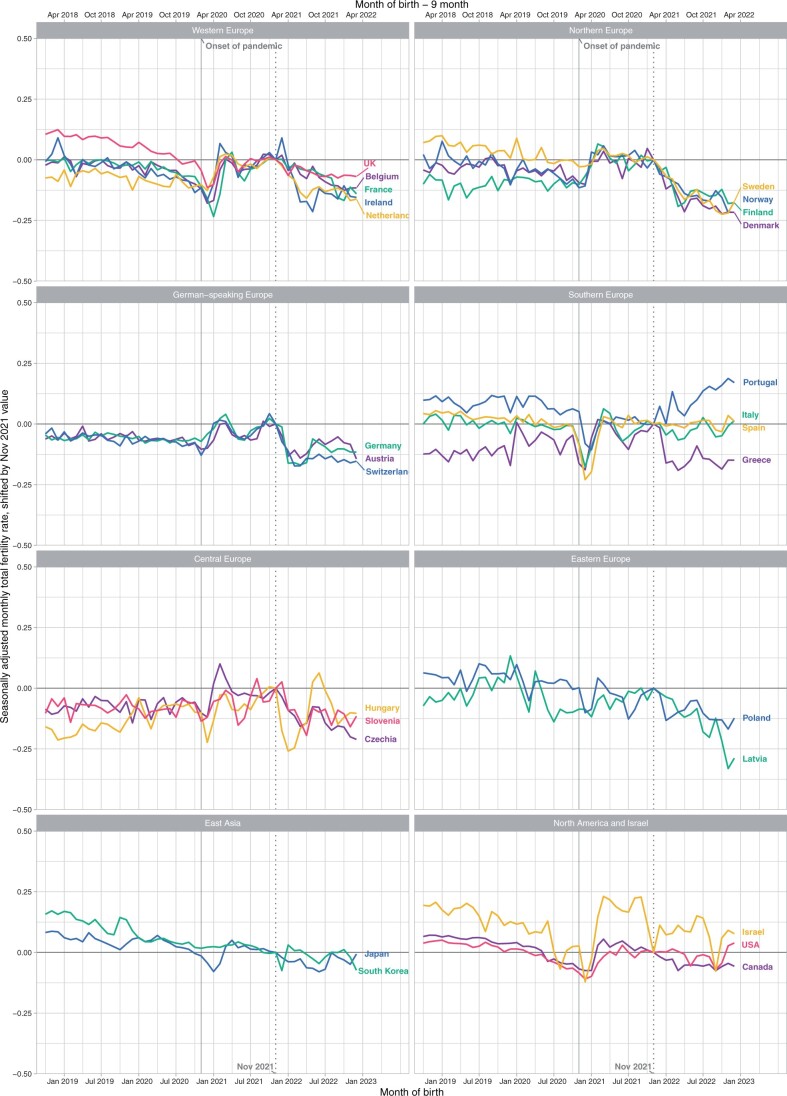
Seasonally adjusted monthly total fertility rates relative to the November 2021 level (November 2021 = 0), November 2019 to December 2022.

Figure 1 depicts the monthly trends in the TFR for all countries analysed by both the month of birth and the estimated month of conception from November 2019 to December 2022. The country TFRs are shown relative to those in November 2021 to enhance comparability and to demonstrate the extent of changes in the TFRs in early 2022 (for trends in absolute TFRs, see Supplementary Fig. S1). The figure reveals variations in birth trends across countries and broader regions, with common periods of ups and downs. These include especially the initial pandemic dip in birth rates around December 2020 and January 2021 (with conceptions around April 2020), a brief recovery two months later, and a decrease in birth rates since early 2022. This recent decrease in the TFR was pronounced in the Nordic countries, Western Europe (except in the UK), German-speaking countries (Austria, Germany, Switzerland), and Czechia, Hungary, Slovenia, Poland, Latvia, and Greece. In contrast, most countries outside Europe (Japan, South Korea, Canada, the USA) and Southern European countries (except for Greece) did not experience any sustained downward trend in TFRs since early 2022, whereas the TFR decline in Israel took place in late 2021.

The decline in TFRs was especially sharp in the first months of 2022 (in a few countries it started already in December 2021, and in Israel in October 2021), often with a temporary recovery (e.g. in Hungary, Germany, Austria, Ireland, Denmark, Finland, Greece, and Poland) around June 2022. This was mostly followed by a renewed, albeit more gradual, decline since August 2022. For instance, the estimated seasonally adjusted TFR in Germany decreased by approximately 10%, from 1.58 in December 2021 to 1.42–1.43 in January–April 2022, and then recovered to values at or above 1.50 in May–July 2022 before declining slightly again. Across all analysed countries, the TFR decreased on average by 0.10 between November 2021 and April 2022, a relative decline of 6%.

**Figure 2.**
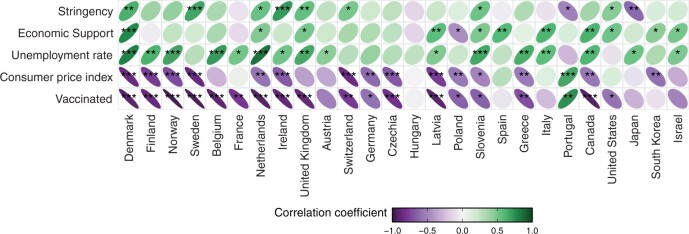
**Summary of correlation coefficients of explanatory variables with the monthly seasonally adjusted total fertility rate by country**. Computations by the authors; visualization was adopted from Cai et al. (2022). *P < 0.05, **P < 0.01, ***P < 0.001.

Figure 2 summarizes the correlation of the individual explanatory variables with the monthly seasonally adjusted TFR, where countries are clustered by geographical region. The colour and the angle of the ellipses indicate the direction of the correlation: purple and left-rotated ellipses represent negative correlations, and green and right-rotated ellipses denote positive correlations. The darker the colour and the ‘thinner’ the shape of the ellipse are, the stronger the correlation. The pale solid circles indicate that the series of the respective explanatory variable is unrelated to the TFR time series during the pandemic.

We found that the stringency index is positively associated with birth trends in most countries. The exceptions to this pattern are Japan and Portugal, where the TFR was significantly lower during stricter containment measures. This suggests that the decline in the stringency of pandemic containment measures and the associated increase in mobility were associated with a decline in TFRs.

Governmental economic support aimed to cushion economic uncertainty among the population and, accordingly, was positively correlated with TFRs during the pandemic. Strikingly, the unemployment rate was also positively associated with birth trends in almost all countries, challenging well-established findings about the negative impact of unemployment on birth rates. In line with past research, the consumer price index was strongly negatively correlated with childbearing in almost all countries.

Finally, vaccination rollout was also strongly negatively correlated with TFRs in almost all countries, with the most notable exception being Portugal, where the TFR increased along with vaccination take-up.

**Figure 3.**
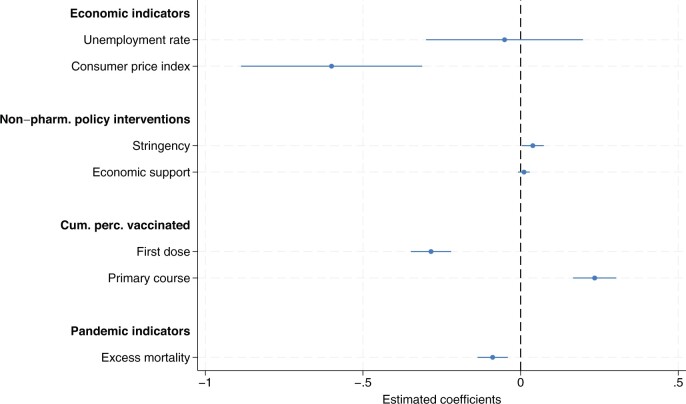
**Estimated model coefficients of the fixed effect model of the monthly seasonally adjusted total fertility rate per 100 women**. The regression model includes additional controls for the first COVID-19 wave and country-fixed effects. Cum. perc., cumulative percentage; Non-pharm., non-pharmaceutical.

Figure 3 plots the estimated model coefficients of the fixed effects regression model of the seasonally adjusted TFR per 100 women (see also Supplementary Table S1). The model includes economic indicators, two NPI indicators and indicators of vaccination rollout while additionally controlling for the health emergency and the first COVID-19 wave. For the economic indicators, we did not find the expected negative association with the unemployment rate (β = −0.051, 95% CI: −0.300 to 0.200). However, due to massive government interventions, the unemployment rate might not properly reflect economic uncertainty during the pandemic (OECD, 2020). In only a few countries, including Canada and the USA, unemployment surged after the onset of the pandemic (e.g. Kearney and Levine, 2023). The negative impact of the consumer price index on birth trends was confirmed in the multivariable analysis (β = −0.600, 95% CI: −0.887 to −0.312). On average, consumer prices increased for all countries by approximately five percentage points in 2021, with the Czech Republic and Poland experiencing a 10% point increase. Considering the average increase of five percentage points, higher inflation would be associated with a monthly decline in the TFR of three births per 100 women in the period of October 2021 to September 2022.

With regard to the non-pharmaceutical policy measures, we observed a small but statistically significant positive association between the stringency index and the TFR (β = 0.039, 95% CI: 0.004–0.073), while economic support did not show the expected positive association (β = 0.011, 95% CI: −0.008 to 0.029). In an alternative model, the normalcy index developed by The Economist (2021) was included instead of the stringency index in the regression model (see also Supplementary Table S2). The normalcy index was designed to reflect changes in the population’s mobility and activities outside of the home. It is composed of eight indicators covering three different domains (transport and travel; recreation and entertainment; and retail and work) where each indicator is measured as a percentage of its pre-pandemic level (as the average values of each indicator in January and February 2020). As the correlation between the normalcy index and the stringency index is high (rho = −0.88, *P* < 0.001), we did not include both indices in our analysis simultaneously. The estimated coefficient for the normalcy index was not significant (*P* = 0.218). However, the normalcy index has several important caveats. It is relative to a pre-pandemic level, which is only measured in January and February 2020, and it may therefore be biased by seasonal variations in the indicators of the different domains. Furthermore, it is not available for all countries considered in this study.

Our results suggest that the age-graded, two-dose vaccination scheme resulted first in lower TFRs and later was linked with their recovery. When the vaccination rollout gained momentum and the cumulative share of the population having received at least one dose of the COVID-19 vaccine increased, TFRs decreased (β = −0.260, 95% CI: −0.324 to −0.197). This suggests that some women chose to delay their pregnancies until after completing their vaccination. Indeed, we found a statistically significant positive association between the cumulative percentage of the population that completed the primary vaccination course and the TFR (β = 0.213, 95% CI: 0.144–0.281). It is worth noting that the estimated coefficient is almost equal in magnitude to the estimated negative coefficient of the indicator signalling the start of the vaccination rollout. In the spring of 2021, the share of the population that had received the first dose of the vaccination increased on average by 12 percentage points per month. This would be associated with an average decline of approximately three births per 100 women per month in the period from January to March 2022. In early summer, the share of the population completing the initial vaccination protocol similarly increased. This would be then associated with a compensatory average increase in the TFR of approximately 2.6 births per 100 women per month in the period from March to May 2022. However, the timing and pace of vaccine roll-out varied markedly across countries. In fact, some countries experienced increases in the share of the population vaccinated that were more than double the average numbers stated above.

Due to the age-graded nature of the vaccination roll-out, women of childbearing age were entitled to the vaccination in late spring or early summer 2021 in most countries. For a subset of countries, vaccination data are also available by broad age groups. As a robustness check, we included the share of the population of childbearing age instead of the total population in the regression model. This revealed a similar pattern of delay and recovery of births during the primary course of vaccination (see also Supplementary Table S2).

Finally, the regression model included two controls for the health emergency and the first COVID-19 wave (see Supplementary Table S1). The health crisis, as measured by excess mortality, was negatively associated with TFRs (β = −0.089, 95% CI: −0.127 to −0.051). The estimated coefficient for the early pandemic months, February to April 2020, is −8.275 (95% CI: −9.721 to −6.829), which is consistent with the markedly depressed TFRs nine months later, i.e. November 2020 to January 2021 (not shown in Fig. 3 due to the large effect size).

**Figure 4.**
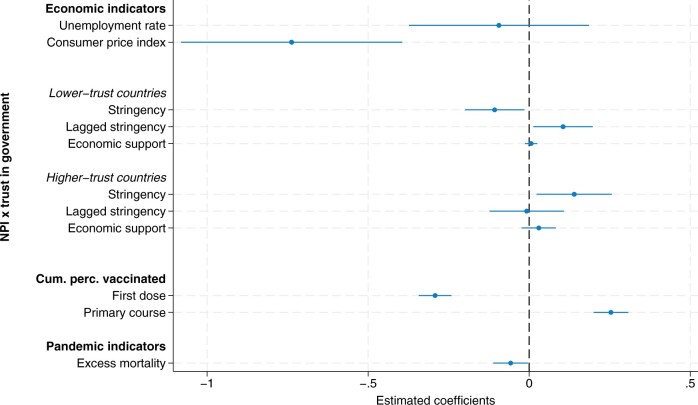
**Estimated model coefficients of the fixed effect model of the monthly seasonally adjusted total fertility rate per 100 women, distinguishing non-pharmaceutical policy interventions (NPI) in lower- and higher-trust countries**. The regression model additionally includes a time dummy for the first COVID-19 wave and country-fixed effects. Cum. perc., cumulative percentage.

Next, in Fig. 4, we inspect the association of NPIs with TFRs by first including the lagged stringency index and adding an interaction between the policy responses (stringency, lagged stringency, and economic support) and the level of trust in government in the country (see also Supplementary Table S1). In countries with lower levels of trust in government, stricter containment measures were associated with a statistically significant decrease in TFRs (β = −0.107, 95% CI: −0.200 to −0.015). The positive coefficient of the lagged stringency variable (β = 0.105, 95% CI: 0.013–0.198) suggests that births subsequently partly recovered. However, we did not find any evidence of such containment measure-associated postponement of births in countries with higher institutional trust. On the contrary, periods of stricter containment measures in higher-trust countries was associated with a statistically significant higher TFR (β = 0.140, 95% CI: 0.023–0.256) without any indication of a later ‘compensatory’ decline (β = −0.008, 95% CI: −0.123 to 0.108). Conversely, a decline in the stringency of measures were associated with depressed TFRs again. For instance, a reduction in the stringency index of approximately 10 points in one month would be associated with a decline in the TFRs of 1.4 births per 100 women in higher-trust countries nine months later.

Unlike the results for the stringency index, we did not find any visible country differences in the association between economic support and TFRs. In both country groups, the estimated effects were small and not significantly different from zero (β = 0.006, 95% CI: −0.013 to 0.198, and β = −0.030, 95% CI: −0.023 to 0.083, in lower- and higher-trust countries, respectively). While economic support cushioned economic uncertainty and income loss, it was not linked to childbearing behaviour during the pandemic when simultaneously considering economic indicators, containment measures, and vaccination rollout.

**Figure 5.**
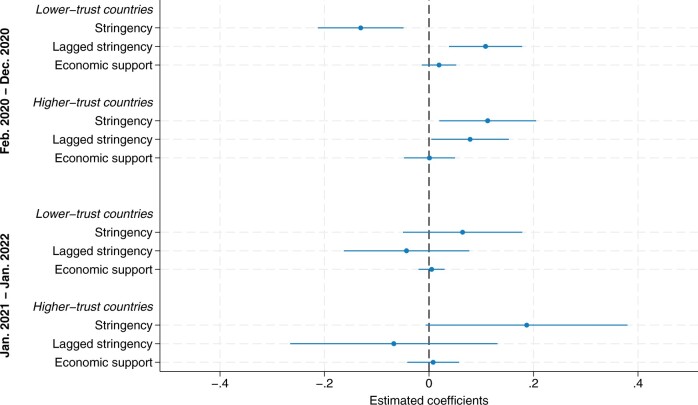
**Estimated model coefficients of the fixed effect model of the monthly seasonally adjusted total fertility rate per 100 women, for non-pharmaceutical policy interventions by level of trust and period of conception**. The regression model also includes the covariates representing the economic indicators and the vaccination rollout indicators as well as the controls for the severity of the pandemic and country fixed effects.

The vaccination rollout and the prospect of a return to normality may have altered the relationship between NPIs and TFRs over the course of the pandemic. In a further analysis, we thus estimated the link between NPIs and TFRs separately during the early and a later phase of the pandemic. Figure 5 displays the estimated coefficients for containment and economic support measures in lower- and higher-trust countries for two periods, February 2020 to December 2020, versus January 2021 to January 2022 (see also Supplementary Table S1). We confirmed the previously derived pattern of a decline and recovery of births in lower-trust countries, but only for the early phase of the pandemic. For conceptions from January 2021 to January 2022, we found no association between policy interventions and TFRs in lower-trust countries. In higher-trust countries, the stringency index tended to be positively associated with TFRs in both periods, but the statistical significance was weak (*P* = 0.057) in the later period.

As a robustness check, we ran separate regression models distinguishing conceptions in February–April 2020, May–December 2020, and January 2021 to January 2022 (see Supplementary Fig. S3). The results were robust and generally confirmed the findings presented above: The pattern of a decline and recovery of births in lower-trust countries was more pronounced during the first COVID-19 wave than in the remainder of the year 2020, and eventually disappeared in the later phase of the pandemic, as in Fig. 5. In higher-trust countries, the stringency index (or its lagged value) was positively associated with birth trends in all sub-periods. As for the economic indicators, the unemployment rate was not associated with birth trends in any of the sub-periods, while the consumer price index was negatively linked to birth trends during the first COVID-19 wave and in the later phase of the pandemic. In May–December 2020, the results did not show any association between the consumer price index and births. However, this period was characterized by stable prices and relatively stable birth trends. In the regression models, we also controlled for the evolution of the pandemic by including excess mortality. As expected, excess mortality was negatively associated with births during the first COVID-19 wave and during the later phase of the pandemic, but not during the summer and autumn of 2020.

## Discussion

The COVID-19 pandemic was associated with distinct short-term ups and downs in birth trends across low-fertility countries. This study sheds light on the factors driving these fluctuations. We focus especially on the later phase of the pandemic when vaccination became widely available, lockdowns, school closures, and social distancing measures gradually phased out, mobility and socialization outside of the home increased, more people returned to their offices and workplaces, and life partly returned to pre-pandemic ‘normality’. Unexpectedly, birth rates in many European countries dropped starting around January 2022, often putting an end to a minor pandemic-era upturn in births during spring–autumn 2021. Our results suggest that economic uncertainty, NPIs and the first wave of the population-wide vaccination programme contributed to the decline in birth rates during 2022.

One measure of economic uncertainty, inflation, displayed a strong and significantly negative association with birth rates during the pandemic. The rate of inflation across higher-income countries typically decreased during the early phase of the pandemic in 2020 but then began to rise during 2021. The gradual but steady rise in inflation in 2021 thus contributed to the observed decline in birth rates. In 2022, inflation in many OECD countries was more than twice as high as that in 2021. We therefore expect inflation to have a negative and relatively strong impact on birth trends beyond our study period, especially in 2023.

In contrast, the unemployment rate was not associated with birth trends during the COVID-19 pandemic. This unexpected finding could be partly explained by an anomalous labour market situation and policy interventions in the early stages of the pandemic. First, economic activity and unemployment rates in 2020–2021 were strongly affected by the government’s massive interventions to protect jobs and the economy. Most countries, therefore, did not experience strong swings in unemployment (the USA and Canada are the major exceptions among the countries analysed here). Second, social and welfare policies helped to reduce economic uncertainty among people potentially facing employment loss or unstable employment.

The association between containment measures and mobility restrictions on birth rates, as measured by the stringency index, differed between lower-trust countries and higher-trust countries. In countries with higher levels of trust, stricter policies were linked with higher birth rates, partly explaining the temporary upturn in births observed, e.g. in the Nordic countries, Germany, and the Netherlands. For some couples, especially in countries with stronger economic and family policy support, greater trust in government and less disruptive impacts of COVID-19-related policies on everyday life, this was a favourable time to have children despite the pandemic (Nisén *et al.*, 2022; Lappegård *et al.*, 2023; Bujard and Andersson, 2024). In spring 2021, the stringency of the containment measures was gradually reduced, putting an end to the previously observed birth rate-enhancing cocooning effect. Hence, TFRs in 2022 were lower compared to the preceding year.

In contrast, stricter containment policies in lower-trust countries were associated with a decline and subsequent recovery of births, in line with the findings of Plach *et al.* (2023). Less stringent policies would thus be linked to a recovery of births. However, our analysis revealed that the pattern of birth decline and recovery disappeared for conceptions in 2021. One possible interpretation could be that individuals developed coping strategies with regard to containment policies (Toffolutti *et al.*, 2022), and thus, the link between the stringency index and TFRs vanished. Apart from government policies, actual trends in mobility, work, and socialization outside of the home may also affect birth trends. However, a separate analysis found no evidence for an association between pandemic-related behavioural trends, as reflected by the normalcy index, and the TFR.

The analysis shows a negative association between the initial vaccination rollout and TFRs, whereas the completion of the full first vaccination course (usually consisting of two doses) was linked to a recovery of births. Similar to Bujard and Andersson (2024), we interpret this finding as a behavioural response to the perceived potential risks of vaccination for pregnant women. The decrease in TFRs during the introduction of vaccination was probably due to an ‘anticipatory’ postponement of births, as some women decided to complete their vaccination course before becoming pregnant to minimize the risks of potential COVID-19 infection to their health and pregnancy outcomes. Vaccination was strongly recommended for women with childbearing intentions because of the increased risk of severe illness during pregnancy and the elevated risk of complications during pregnancy due to COVID-19 infection (Wei *et al.*, 2021). Furthermore, women might have been concerned about the hypothetical negative impact of vaccination on their health, fecundity, or pregnancy outcomes. Such concerns have not been substantiated by scientific evidence (e.g. Chen *et al.*, 2021) but have had a strong echo in social media. Successful vaccination campaigns are also closely linked to the ‘return to normal life’ discussed above (Neyer *et al.*, 2022); the launch of broad-based vaccination programs signalled that the pandemic restrictions are likely to be lifted soon and that the pandemic was getting under control. Decisions to postpone pregnancy until the full course of the vaccination had been completed contributed to the downturn in birth rates in January–April 2022 (and, in some countries, already in December 2021) and later to their slight recovery in mid-2022.

In line with the scientific evidence, our analysis does not suggest a direct impact of vaccination on birth trends. Specifically, conceptions in most countries started dropping already in the early stage of the vaccination process, when most women of reproductive age had not yet been eligible for receiving the first dose. If vaccines as such directly contributed to the observed drop in births, birth rates in most countries would have started falling later than observed, and the decline would be of a similar magnitude across all countries during the peak of their vaccination campaigns.

Our research reveals a large cross-country diversity in responses of TFRs to the COVID-19 pandemic (Plach *et al.*, 2023; Sobotka *et al.*, 2023; Nitsche and Wilde, 2024). The downturn in birth rates since approximately January 2022 was visible in most, but not all, of the analysed countries; it did not occur or was only slight in most of the non-European countries studied. In Israel, birth rates decreased earlier, even in October and November 2021, which is consistent with its earlier and more intensive vaccination rollout, with women of reproductive age eligible for vaccination since January 2021 (Mathieu *et al.*, 2020). Portugal is also an outlier where birth rates moved in the opposite direction and started to recover since early 2022, following their steep decline during the main phases of the COVID-19 pandemic. The diversity of birth trends between countries in the wake of the vaccination campaign, also documented by Jasilioniene *et al.* (2024), points out again that COVID-19 vaccination did not have any direct biological impact on birth trends.

Many countries with a mild ‘baby boom’ during the periods of the most severe infection waves and government containment policies experienced an especially sharp downturn in births from early 2022. This includes the Nordic countries, Germany and Switzerland, Czechia, Greece, and Hungary. To some extent, this suggests that a ‘return to normality’ and the end of the ‘cocooning effect’ (i.e. people socializing more outside of the home, attending cinemas, restaurants, sports venues and recreation facilities, travelling again for leisure and recreation, and employees returning to the offices) is a key part of the explanation of declining birth rates. In many countries, the birth trajectory has returned to pre-pandemic trends, characterized by a longer-term decline (Jasilioniene *et al.*, 2024).

Our research demonstrates the usefulness of looking at birth trends and their drivers from a short-term (monthly or even more detailed) perspective rather than taking the usual approach of analysing annual data. Annual data do not have sufficient granularity to study the dynamics and drivers of changes in births during sudden shocks such as the COVID-19 pandemic. However, our research also has limitations. First, the data used in this study do not allow for analysing birth trends by key characteristics, such as age, birth order, and socio-economic status, or by region. Second, our analysis was restricted to higher-income countries with relatively strong social support policies provided by the government as well as wide access to modern contraception. The results may not apply to other country contexts. In middle-income countries, pandemic birth trends showed much larger variation than in the higher-income countries covered here and ranged from long-term drops to temporary increases (Kim *et al.*, 2024). Third, the results may be affected by the construction of the composite indicators employed. For instance, the non-pharmaceutical policy indicators are composite indices built from ordinal indicators. This approach may mask nuances across countries in the strictness of policies within the pre-established policy categories, but also qualitative policy differences at the national and sub-national level that cannot be precisely quantified by categorical indicators. Lastly, given the complexity of the unprecedented and simultaneous developments during the pandemic (Nitsche and Wilde, 2024), the results may also be affected by variables and mechanisms that we have not taken into account and that cannot be subsumed in the country-fixed effects. As a consequence, our findings cannot be interpreted in a causal way.

Our study covers conceptions leading to a live birth in the first two years of the pandemic. It cannot capture longer-term trends or delayed responses to the pandemic and its related policies. This also includes factors we have not been able to analyse, including the disruptions to dating, intimate life, and partnership formation (e.g. Ting and McLachlan, 2022), or long-term health consequences of the pandemic, which may affect birth trends for an extended period of time. The changes in birth rates until autumn 2022 analysed here do not mark the end of the period of unstable, ‘rollercoaster’ birth rates in low-fertility countries. As the COVID-19 pandemic gradually weakened after the vaccination campaign, new crises and disruptions emerged, especially the Russian war against Ukraine since February 2022 and the resulting surge in inflation and economic uncertainty. This pushed birth rates in many of the analysed countries to a new downturn occurring since late 2022 and continuing through 2023 (HFD, 2024), with many countries recording new lows in TFRs.

## Supplementary Material

hoae052_Supplementary_Data

## Data Availability

The data underlying this article were accessed from Human Fertility Database (https://humanfertility.org), OECD (https://oecd.org, DOIs: 10.1787/52570002-en, 10.1787/eee82e6e-en, 10.1787/1de9675e-en), Oxford COVID-19 Government Response Tracker (https://github.com/OxCGRT/covid-policy-dataset), and Our World in Data (https://ourworldindata.org/explorers/coronavirus-data-explorer).
