# Supplementary material for: Birth rate decline in the later phase of the COVID-19 pandemic: the role of policy interventions, vaccination programmes, and economic uncertainty
Source: Hum Reprod Open. 2024 Sep 10;2024(3):hoae052. doi: 10.1093/hropen/hoae052 (PMC11438547; doi:10.1093/hropen/hoae052)
Supplement: hoae052_Supplementary_Data [file hoae052_supplementary_data.pdf]

Supplementary material

Supplement to Winkler-Dworak, M., Zeman, K., and Sobotka, S. (2024). Birth decline in the later phase of the COVID-19 pandemic: The role of policy interventions, vaccination programmes, and economic uncertainty.

Supplementary Table S1: Estimated model coefficients of the fixed effect model of the monthly seasonally adjusted total fertility rate per 100 women.

	(1)<br>Model 1	(2)<br>Model 2	(3)<br>Model 3
**Economic indicators**			
Unemployment rate	-0.051	-0.094	-0.023
	(0.121)	(0.136)	(0.151)
Consumer price index	-0.600***	-0.738***	-0.775***
	(0.139)	(0.167)	(0.154)
**Non-pharmaceutical policy interventions**			
Stringency	0.039*		
	(0.017)		
Economic Support	0.011		
	(0.009)		
Lower-trust countries			
Stringency		-0.107*	
		(0.045)	
Lagged stringency		0.105*	
		(0.045)	
Economic support		0.006	
		(0.009)	
Higher-trust countries			
Stringency		0.140*	
		(0.057)	
Lagged stringency		-0.008	
		(0.056)	
Economic support		0.030	
		(0.026)	
**Conceptions in Feb. 2020–Dec.2020 x non-pharmaceutical policy interventions**			
Lower-trust countries			
Stringency			-0.131**
			(0.040)
Lagged stringency			0.108**
			(0.034)
Economic support			0.019
			(0.016)
Higher-trust countries			
Stringency			0.112*
			(0.045)
Lagged stringency			0.079*
			(0.036)
Economic support			0.001
			(0.024)

**Conceptions in Feb. 2020–Dec.2020 x non-pharmaceutical policy interventions**			
Lower-trust countries			
Stringency			0.064
			(0.055)
Lagged stringency			-0.043
			(0.058)
Economic support			0.005
			(0.012)
Higher-trust countries			
Stringency			0.187^{+}
			(0.094)
Lagged stringency			-0.067
			(0.096)
Economic support			0.008
			(0.024)
**Cumulative percentage vaccinated**			
First dose	-0.284***	-0.292***	-0.245***
	(0.031)	(0.025)	(0.020)
Primary course	0.235***	0.254***	0.213***
	(0.033)	(0.026)	(0.023)
**Pandemic indicators**			
Excess mortality	-0.089***	-0.058*	-0.062^{+}
	(0.023)	(0.026)	(0.033)
First Covid-19 wave	-7.846***	-5.852***	-4.838**
	(0.653)	(1.376)	(1.305)
Constant	215.5***	228.9***	231.9***
	(14.312)	(17.630)	(16.636)
Observations	624	624	624
Countries	26	26	26
Within R-squared	0.424	0.477	0.515

Standard errors in parentheses

$^{+}$ $p < 0.10$, * $p < 0.05$, ** $p < 0.01$, *** $p < 0.001$

Source: Own computations

Supplementary Table S2: Estimated model coefficients of alternative fixed effect model of the monthly seasonally adjusted total fertility rate per 100 women with normalcy index (Model 1A) and vaccination indicators of women of childbearing age (Model 1B).

	(1)<br>Model 1A[a]	(2)<br>Model 1B[b]
**Economic indicators**		
Unemployment rate	-0.388[+]	0.010
	(0.214)	(0.256)
Consumer price index	-0.620[***]	-1.110[***]
	(0.159)	(0.244)
**Non-pharmaceutical policy interventions**		
Stringency		0.017
		(0.024)
Economic Support	0.017	0.031[*]
	(0.013)	(0.012)
Normalcy	-0.027	
	(0.021)	
**Cumulative percentage vaccinated**		
Total population		
First dose	-0.260[***]	
	(0.031)	
Primary course	0.213[***]	
	(0.033)	
Childbearing age		
First dose, Age 25-49		-0.229[***]
		(0.023)
Primary course, Age 25-49		0.208[***]
		(0.038)
**Pandemic indicators**		
Excess mortality	-0.089[***]	-0.079[**]
	(0.018)	(0.020)
First Covid-19 wave	-8.275[***]	-8.094[***]
	(0.696)	(0.792)
Constant	224.0[***]	271.7[***]
	(15.597)	(24.544)
Observations	528	480
Countries	22	20
Within R-squared	0.393	0.435

Standard errors in parentheses

[a] Countries included: Austria, Belgium, Canada, Czechia, Denmark, France, Germany, Hungary, Ireland, Israel, Italy, Japan, Netherlands, Norway, Poland, Portugal, South Korea, Spain, Sweden, Switzerland, United Kingdom, United States

[b] Countries included: Austria, Belgium, Canada, Czechia, Denmark, Finland, France, Hungary, Ireland, Israel, Italy, Latvia, Netherlands, Norway, Poland, Portugal, Slovenia, Spain, Sweden, Switzerland

[+] $p < 0.10$, [*] $p < 0.05$, [**] $p < 0.01$, [***] $p < 0.001$

Source: Own computations

Supplementary Figure S1: Seasonally adjusted monthly total fertility rates from October 2018 to December 2022, by country.

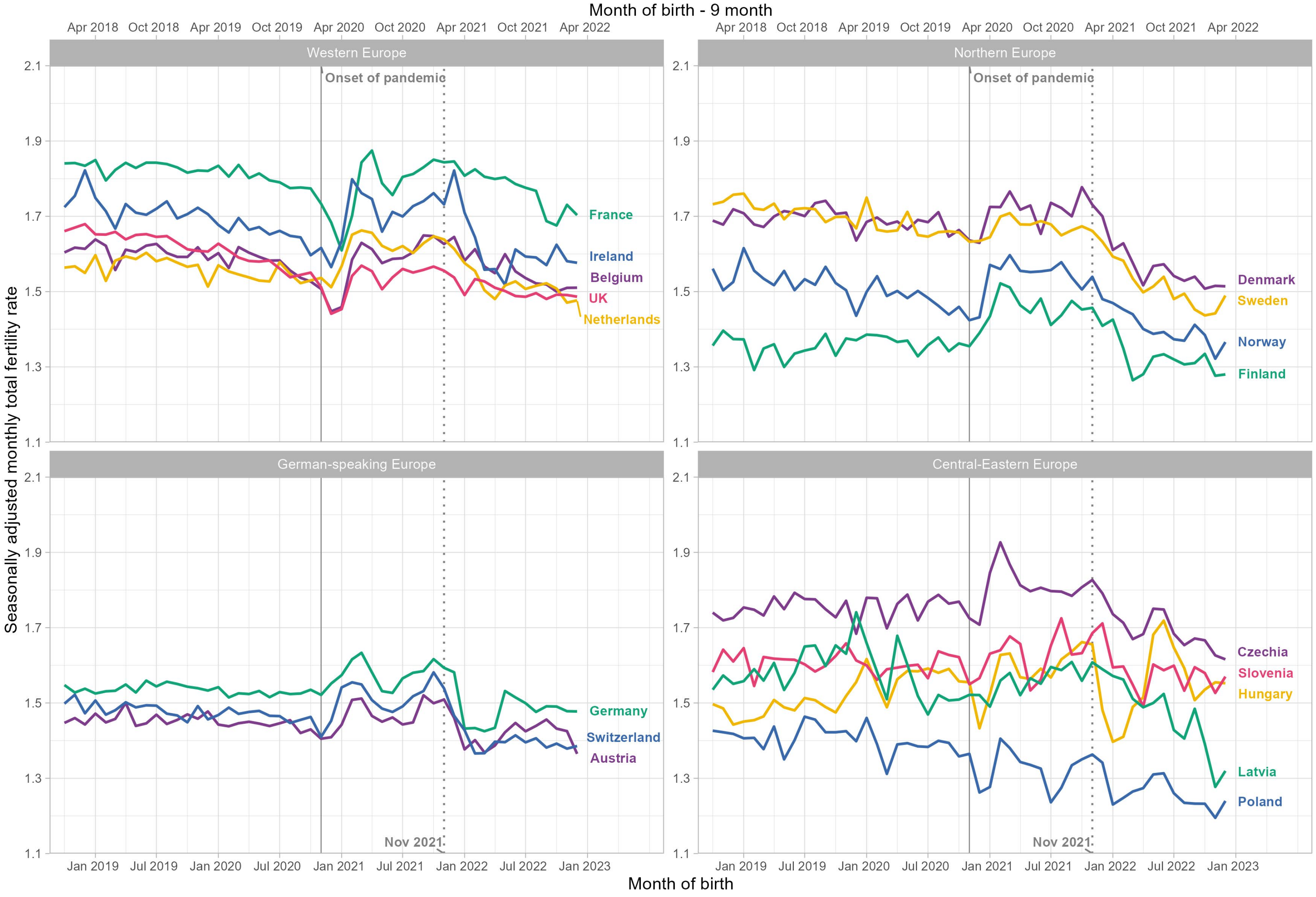

(figure continued on next page)

Supplementary Figure S1: continued

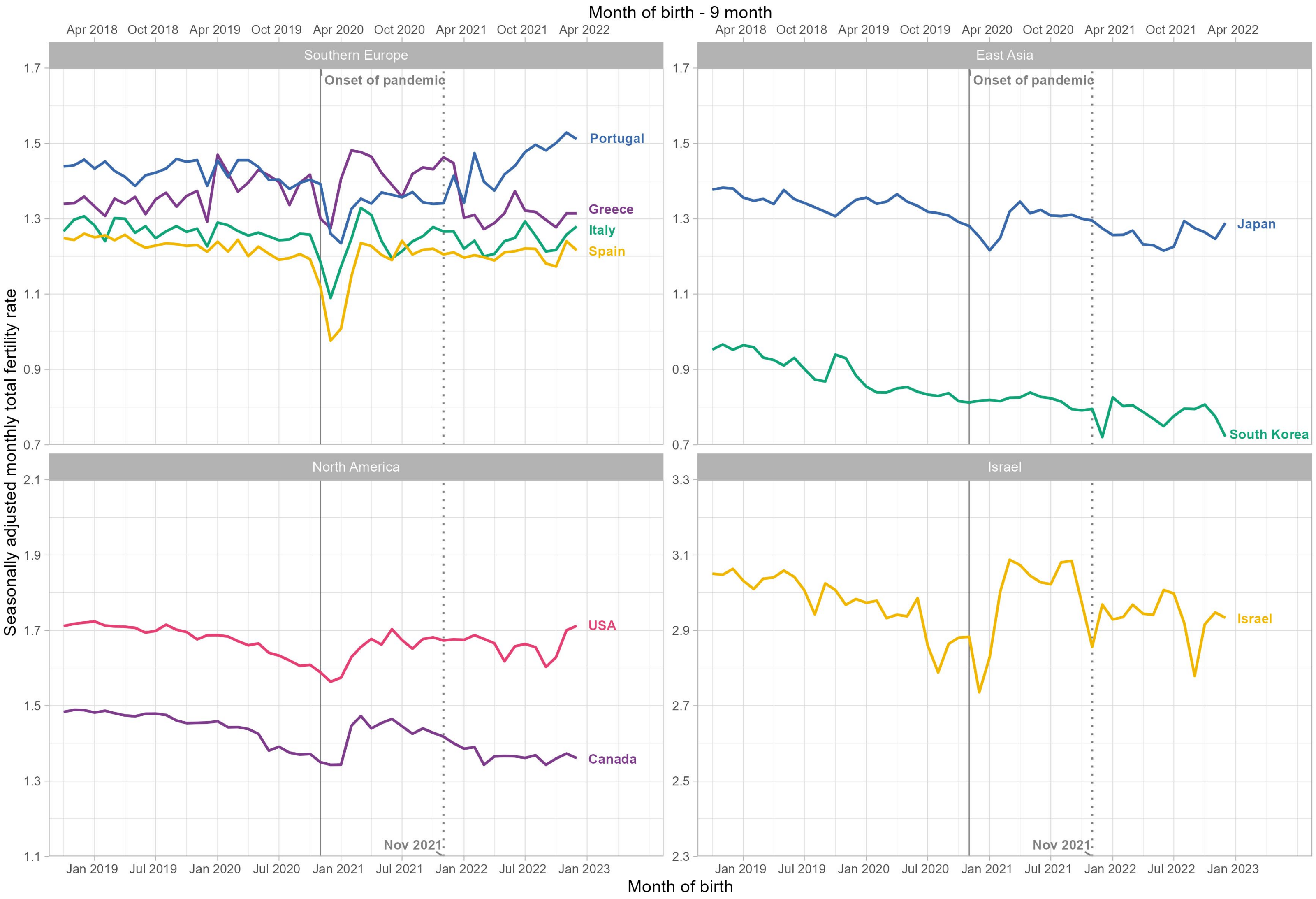

Supplementary Figure S2: Time trends of monthly total fertility rate (TFR) from Oct. 2018 to Oct. 2022 (left axis), and time trends of harmonized unemployment rate, consumer price index (rescaled), stringency index, economic support index, cumulative share of the population that has received at least one dose of the vaccination and that has completed the primary vaccination course, and excess mortality from Jan. 2018 to Jan. 2022 (right axis), by country

Austria

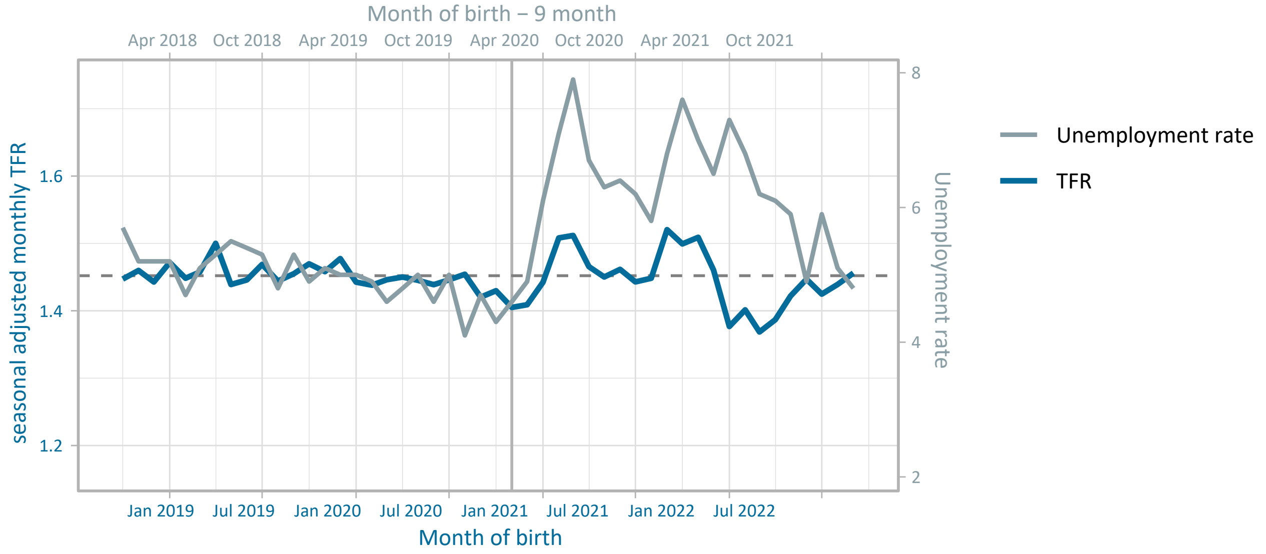

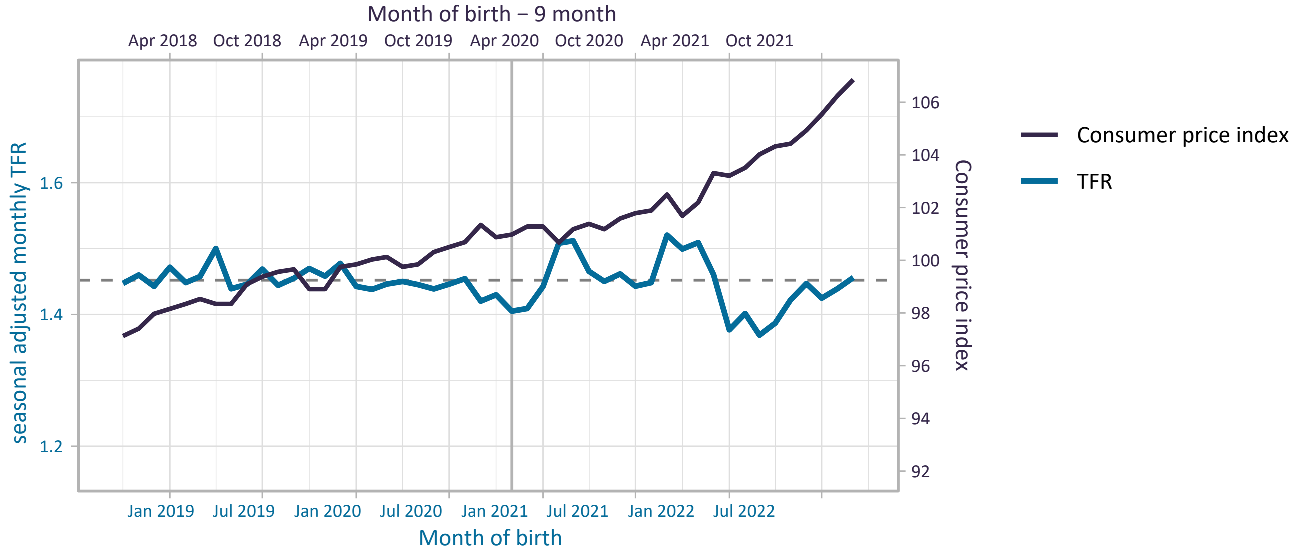

(figure continued on next page)

Austria

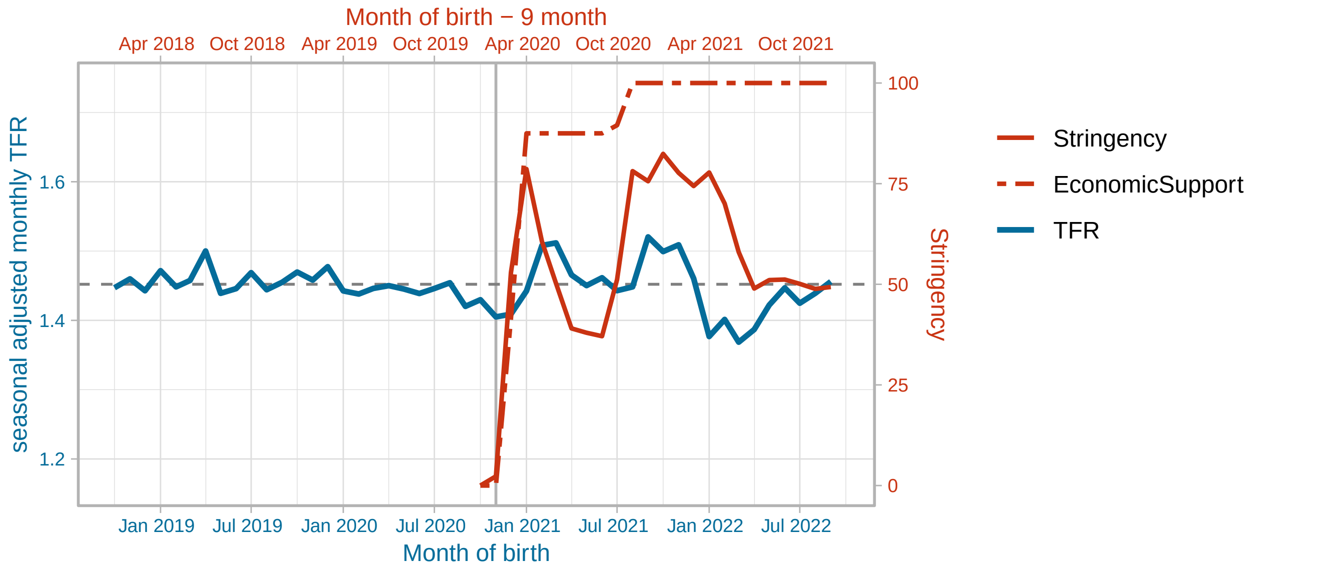

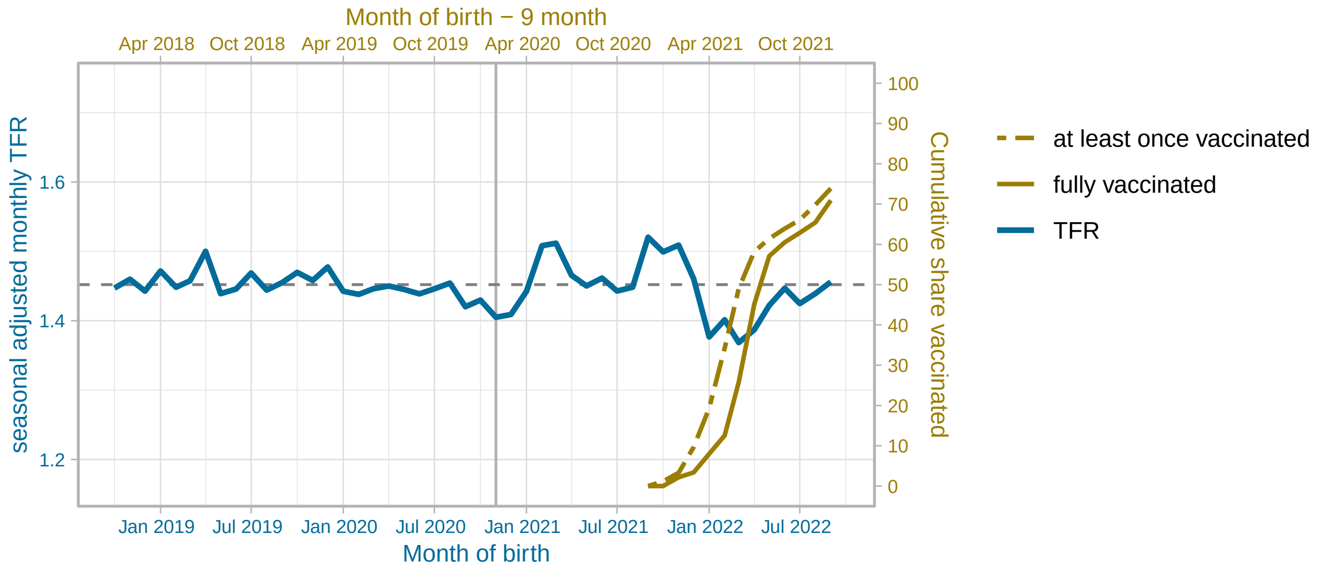

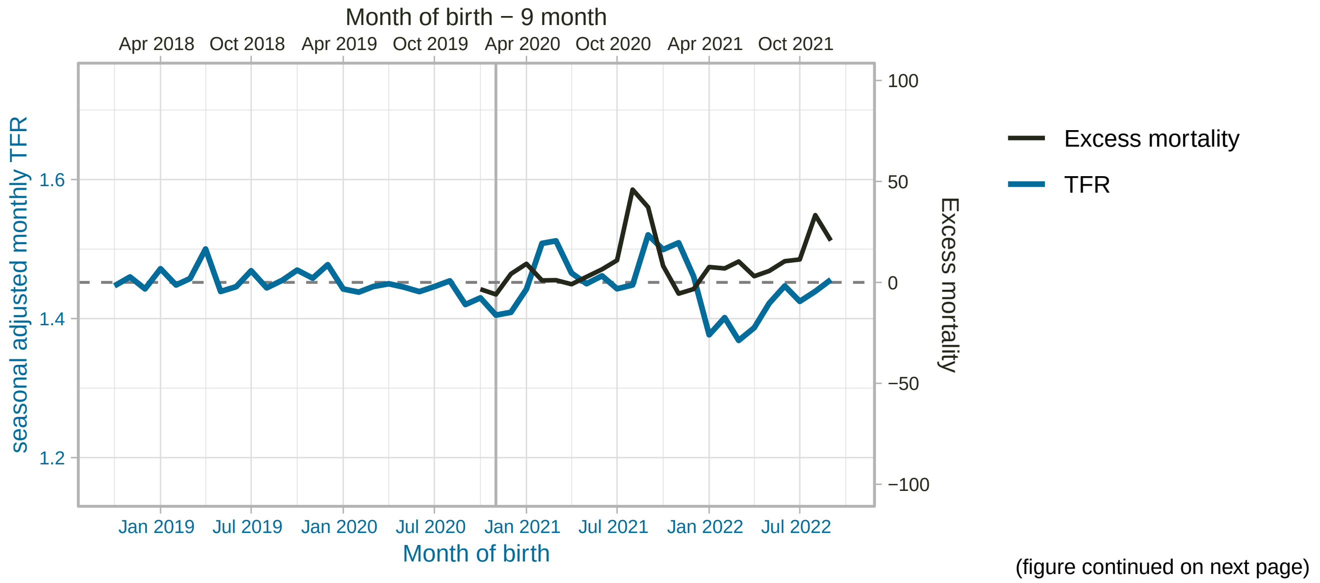

(figure continued on next page)

Belgium

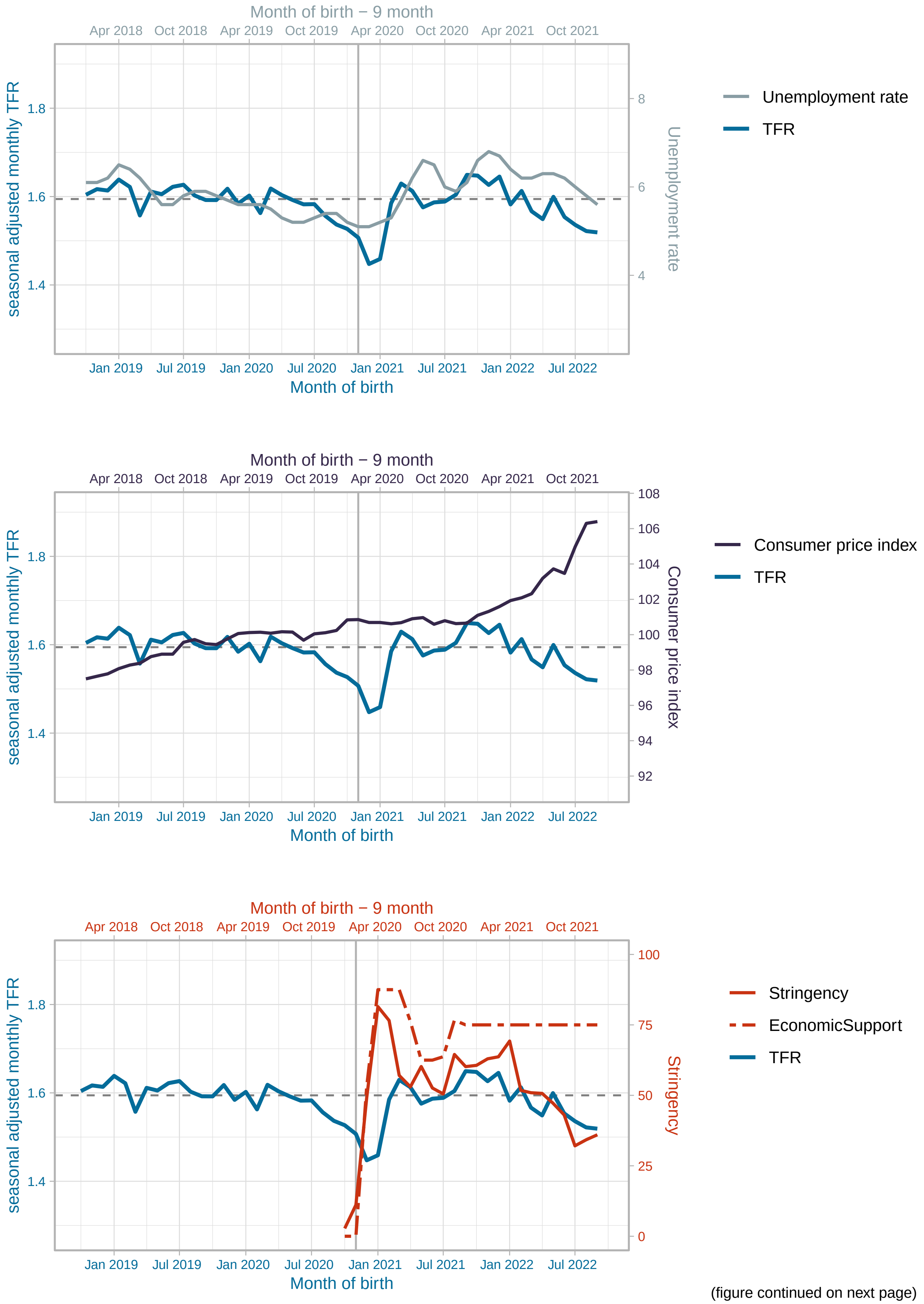

(figure continued on next page)

Belgium

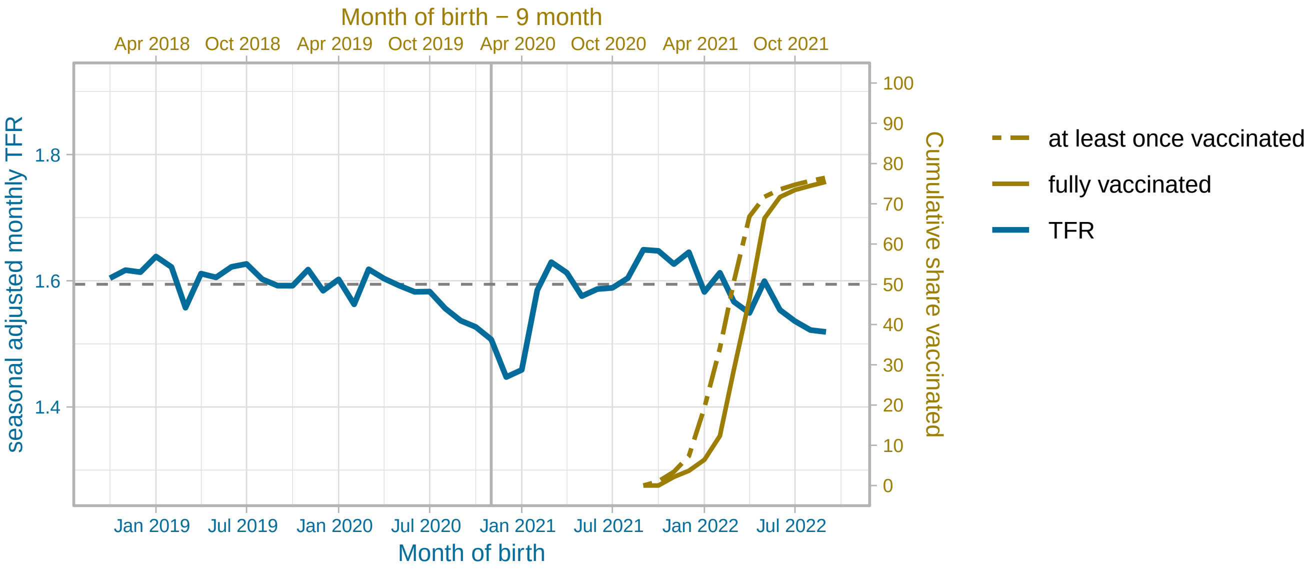

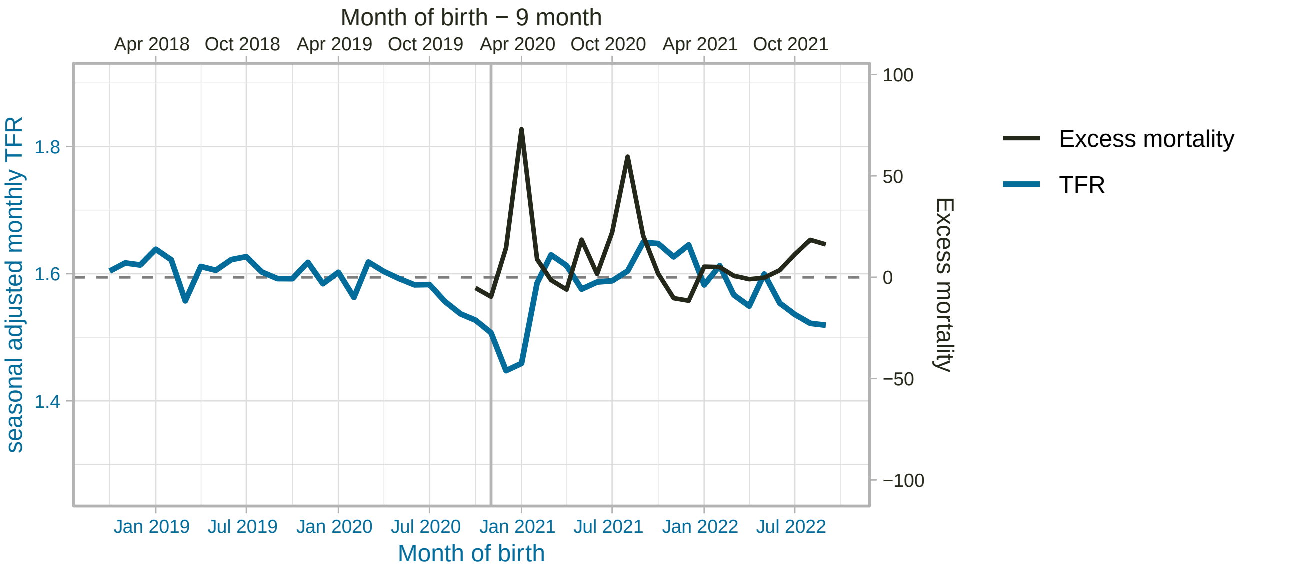

(figure continued on next page)

Canada

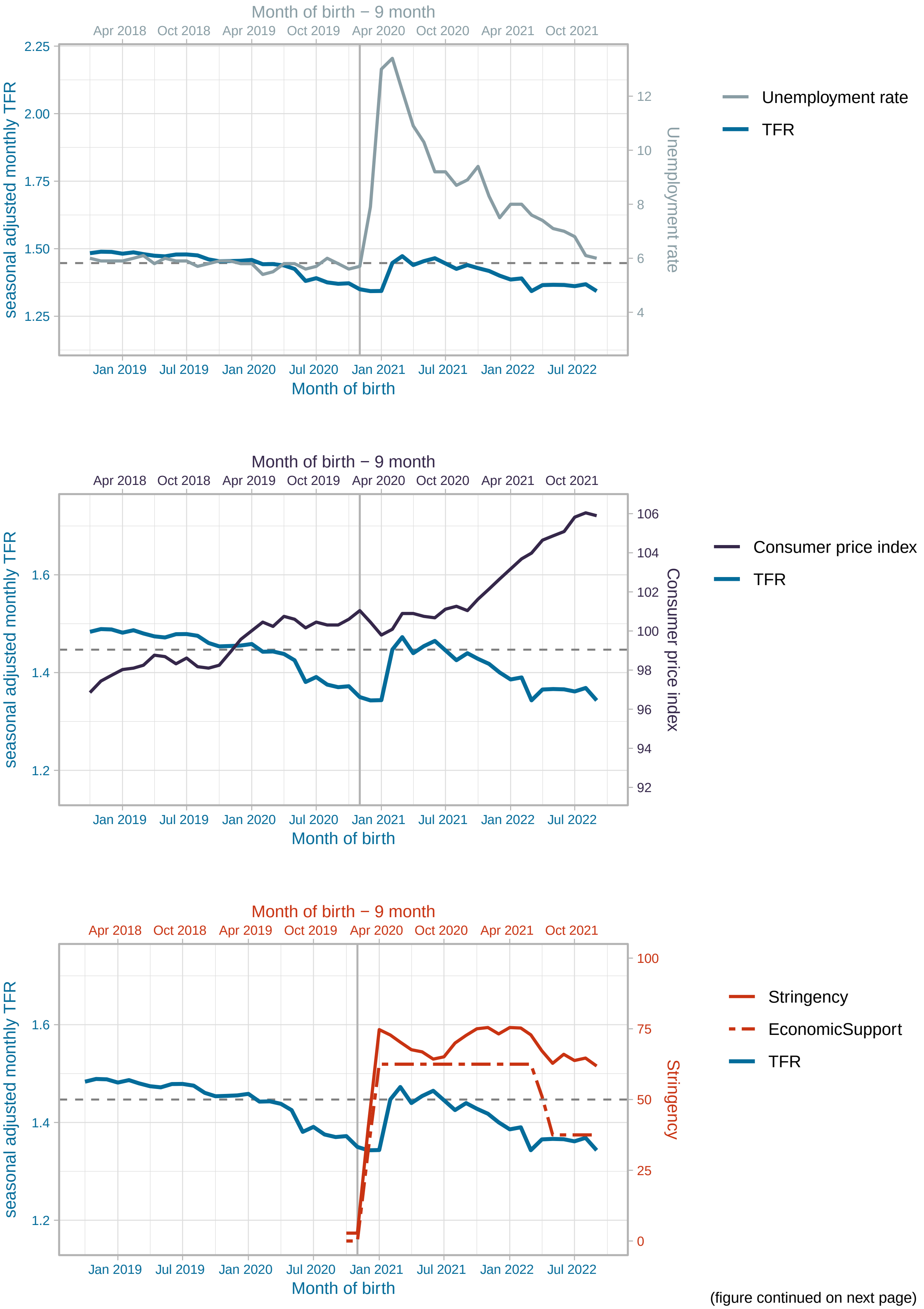

(figure continued on next page)

Canada

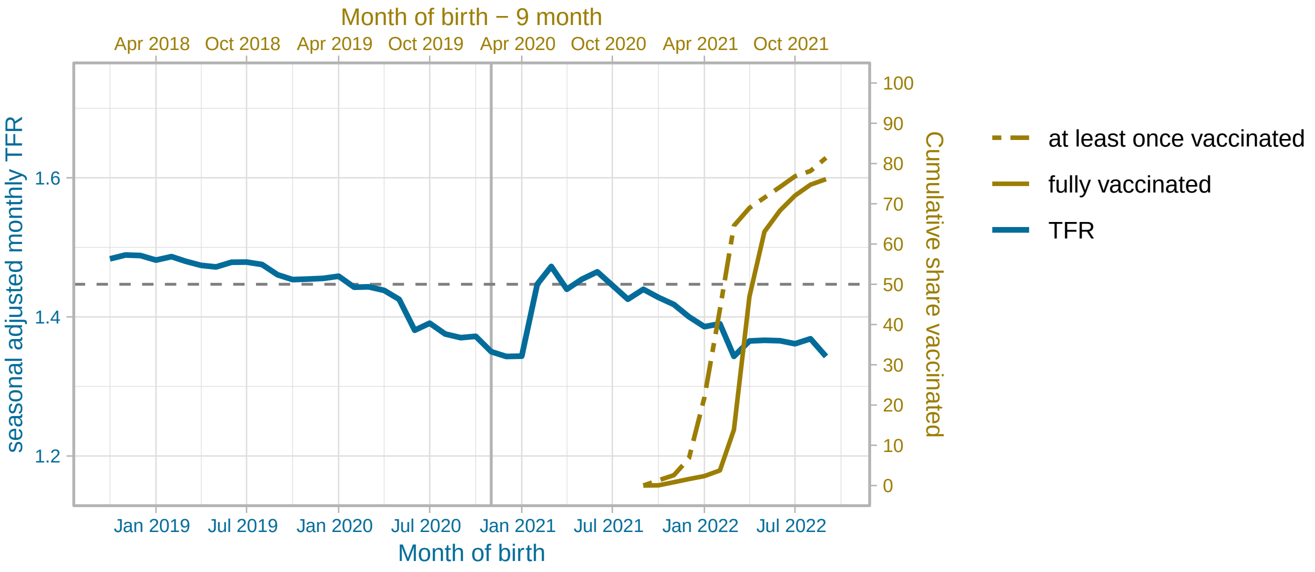

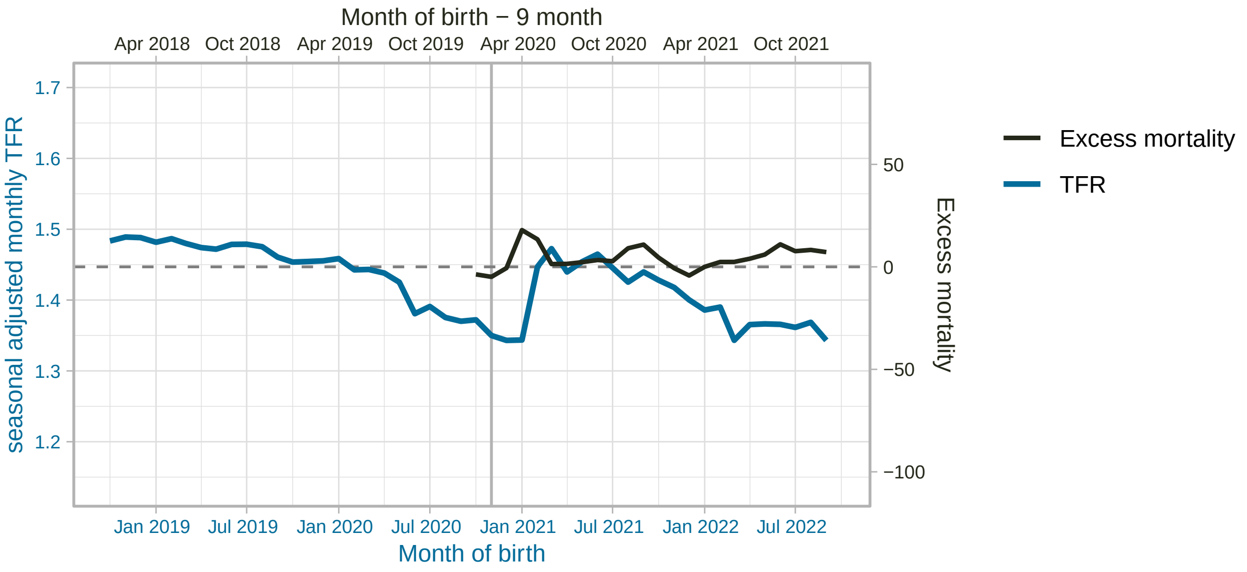

(figure continued on next page)

Switzerland

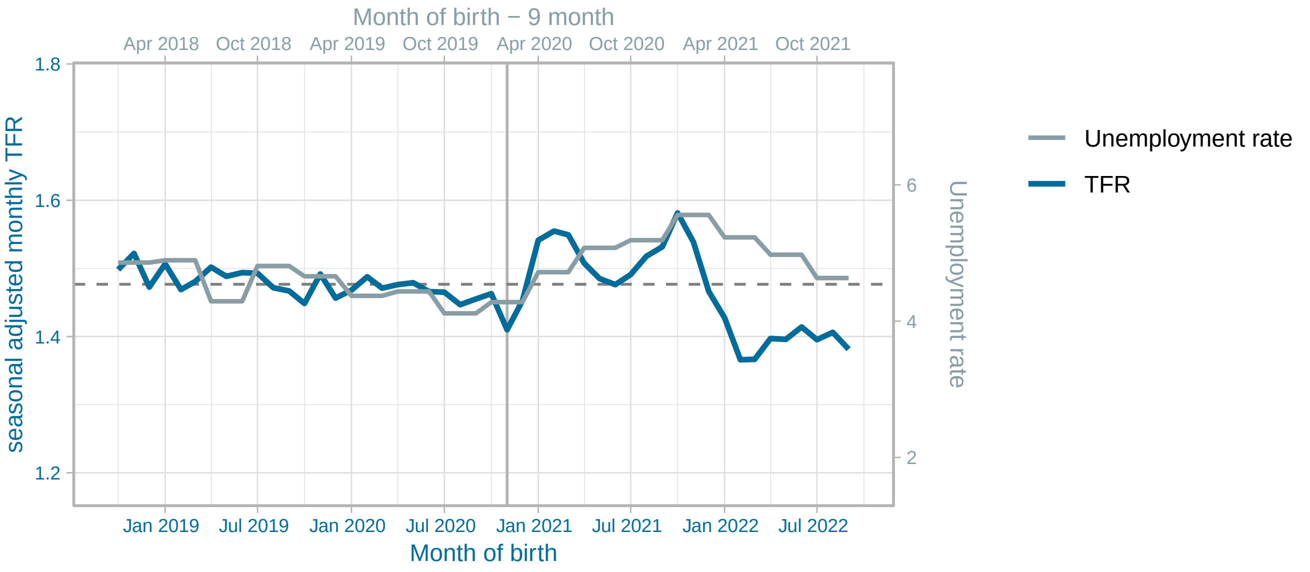

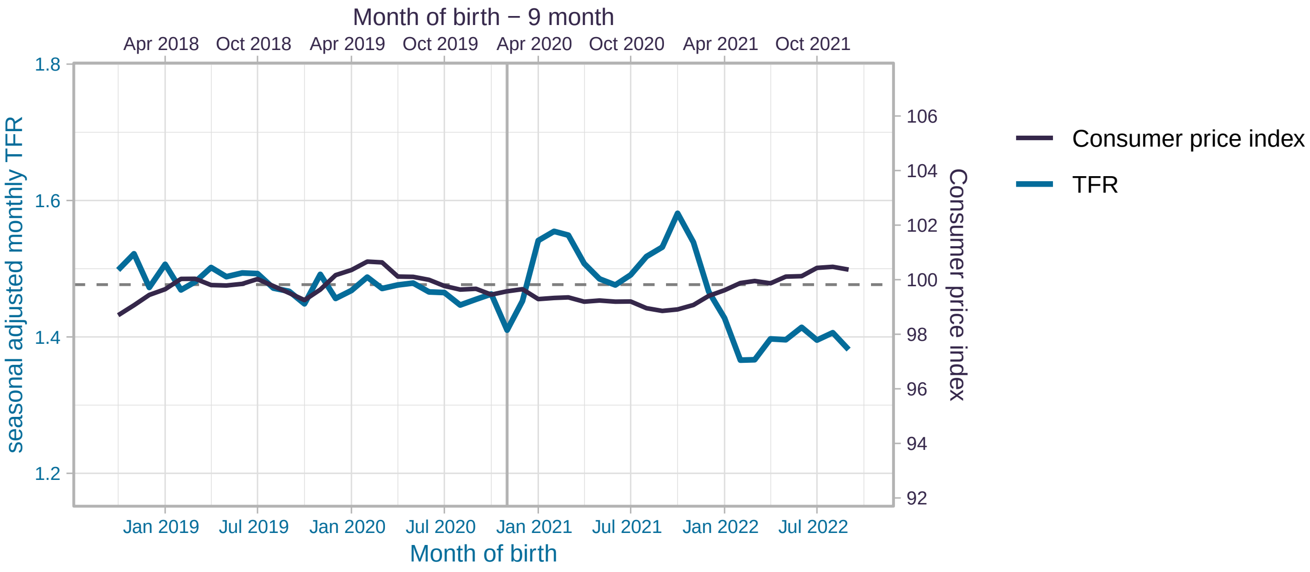

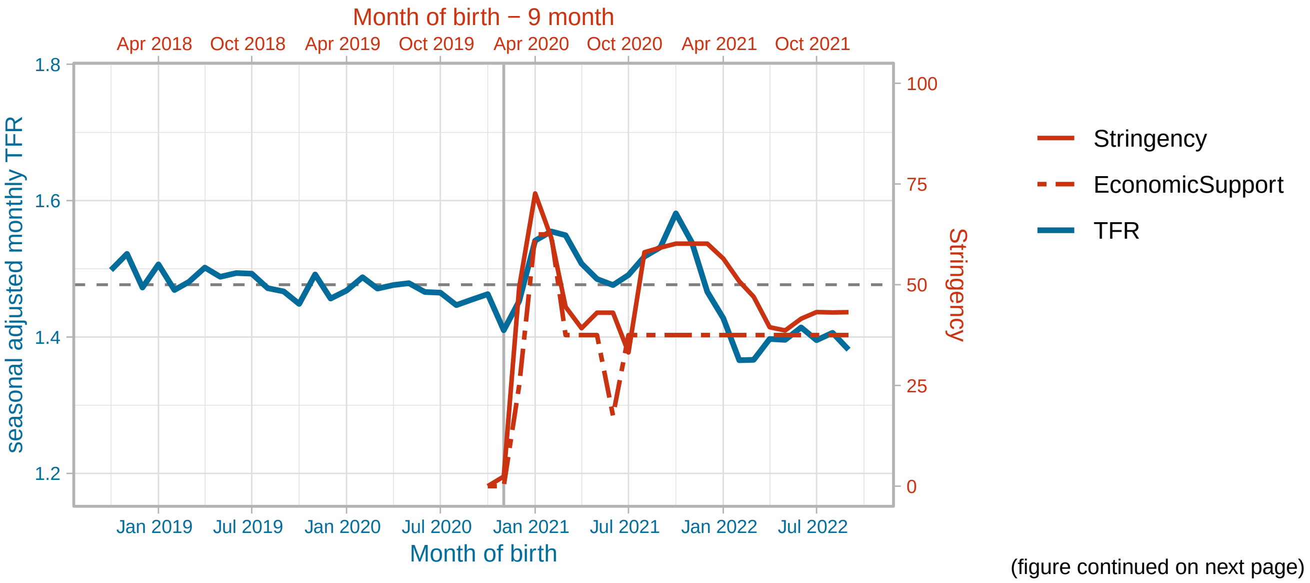

(figure continued on next page)

Switzerland

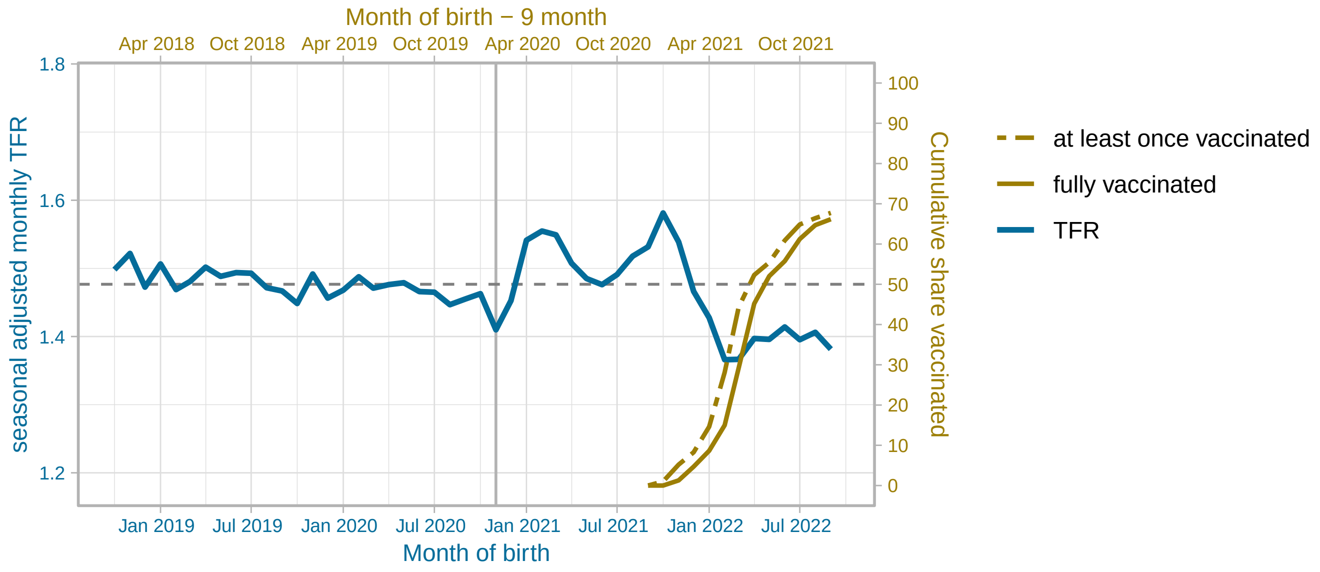

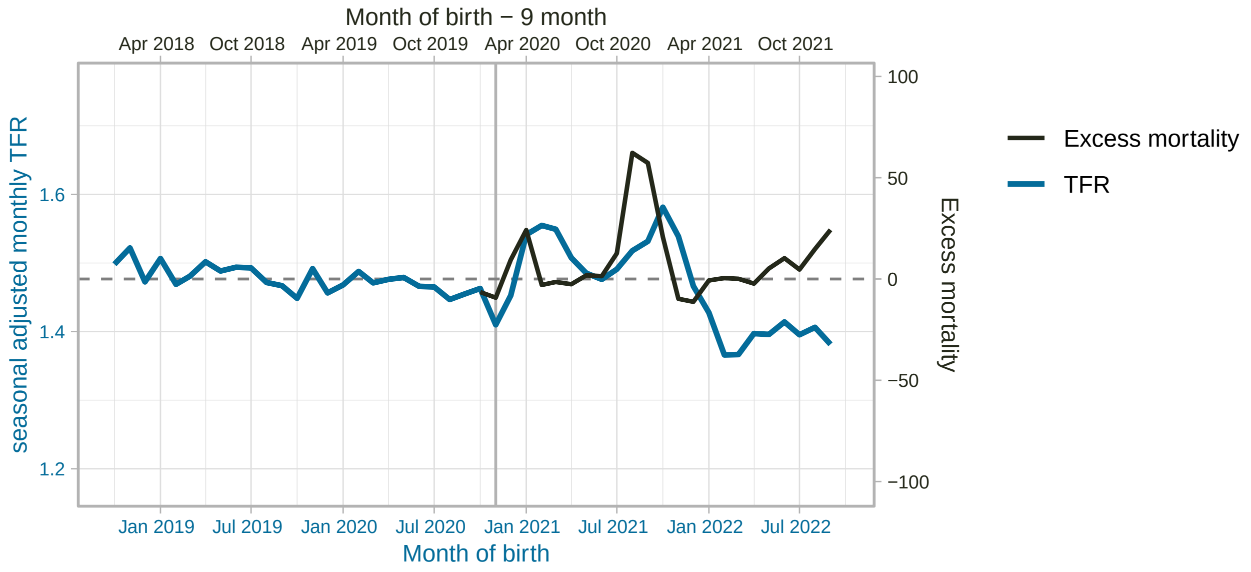

(figure continued on next page)

Czechia

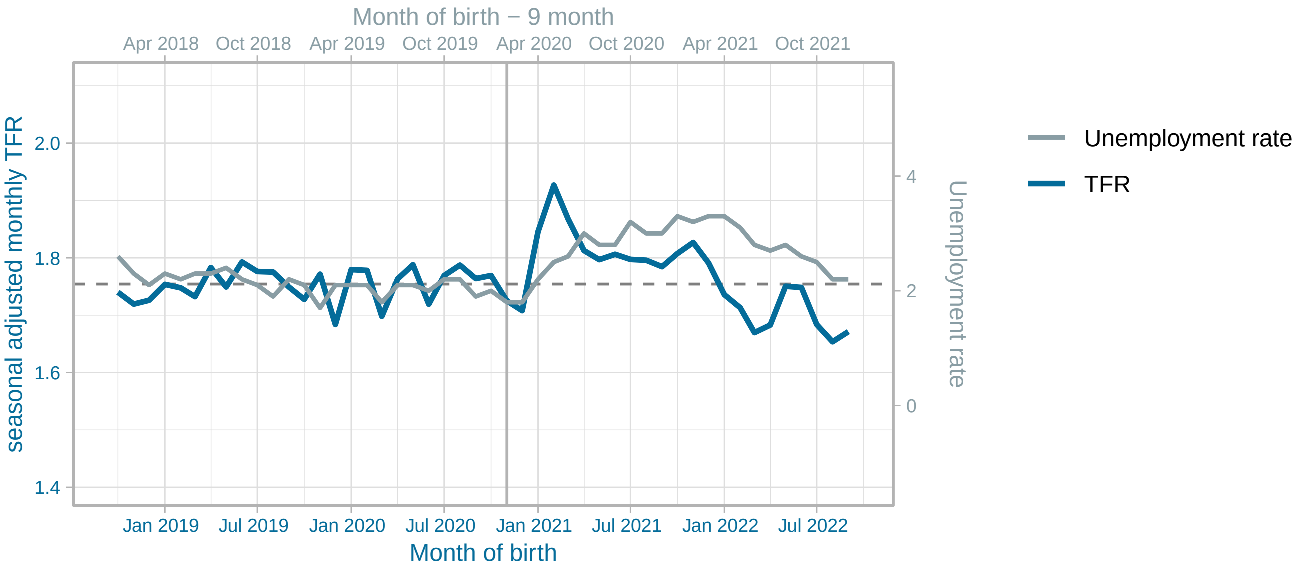

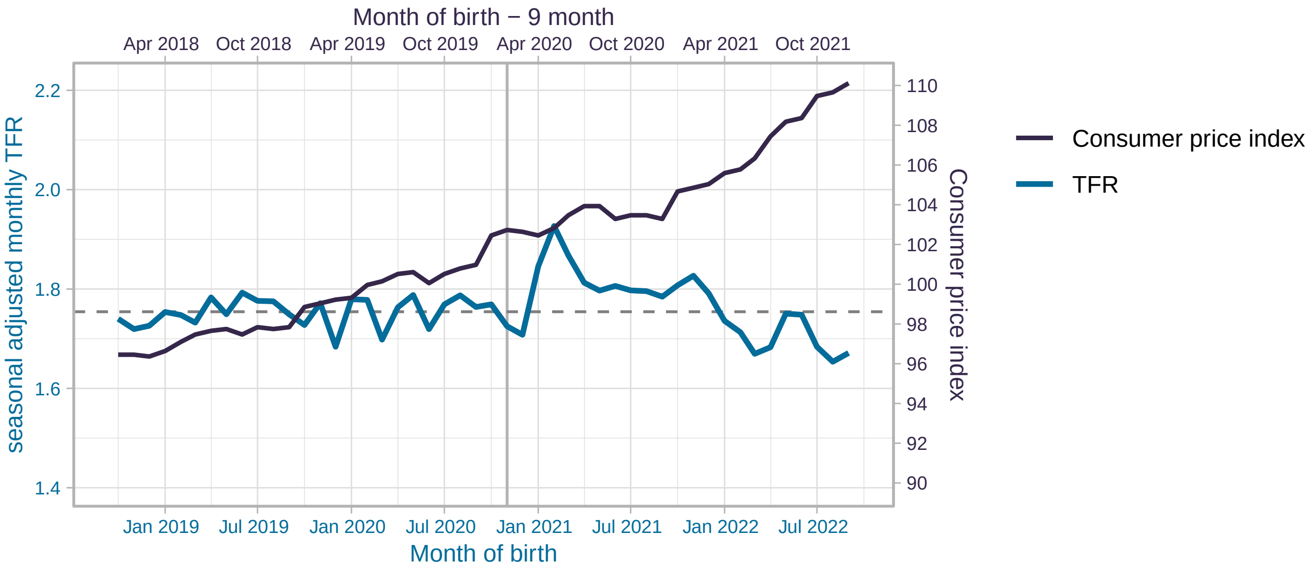

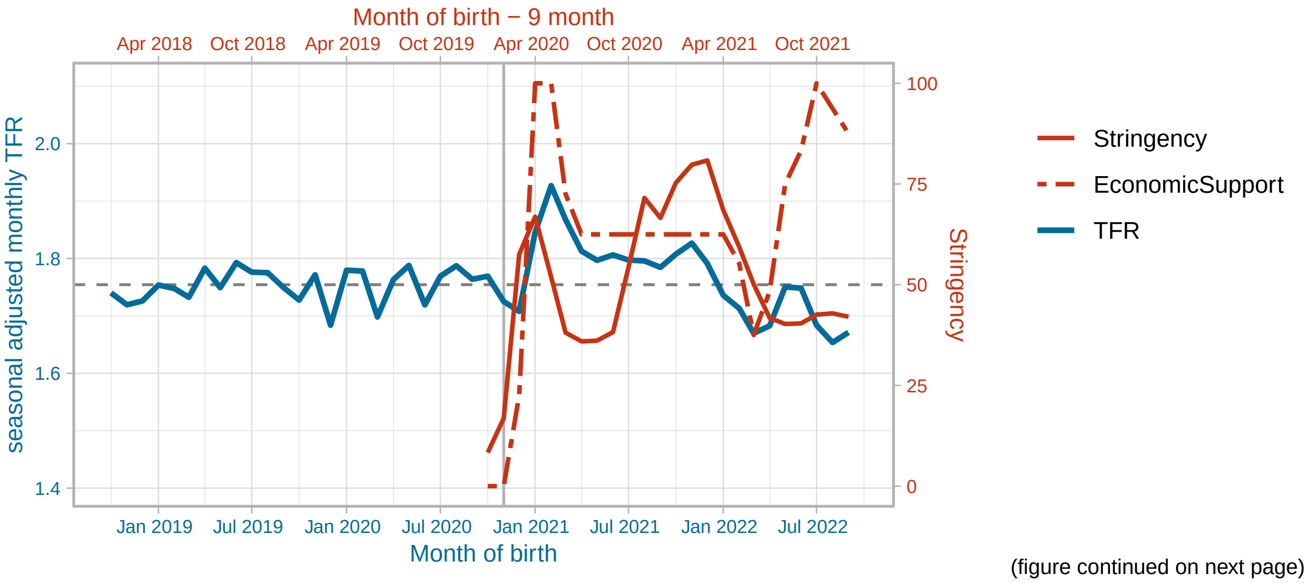

(figure continued on next page)

Czechia

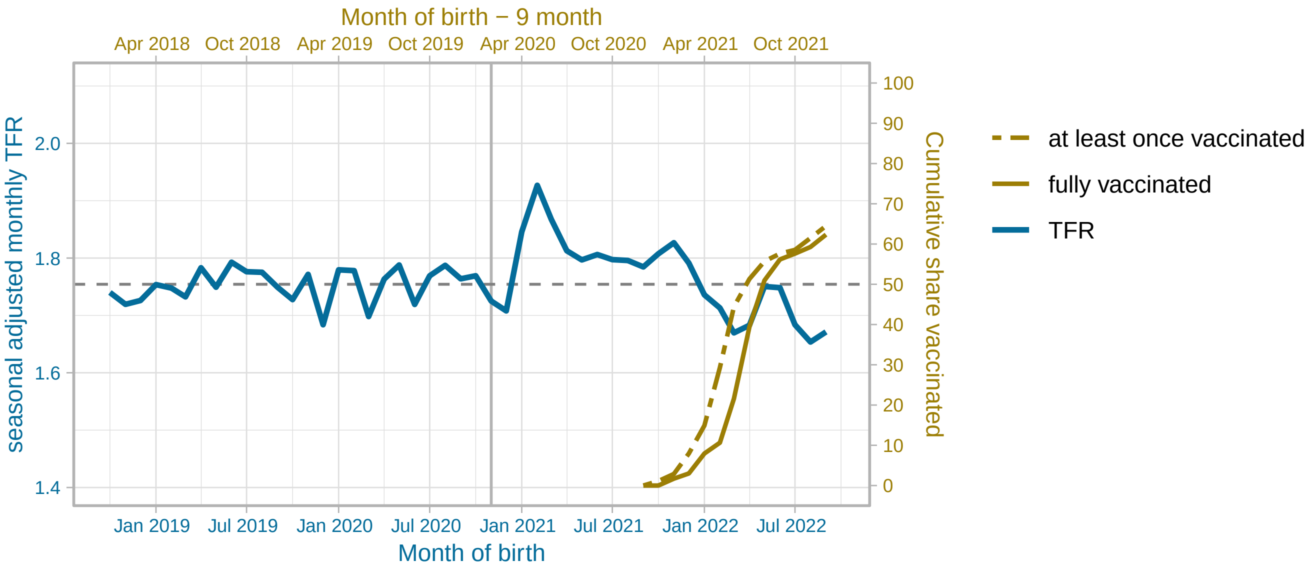

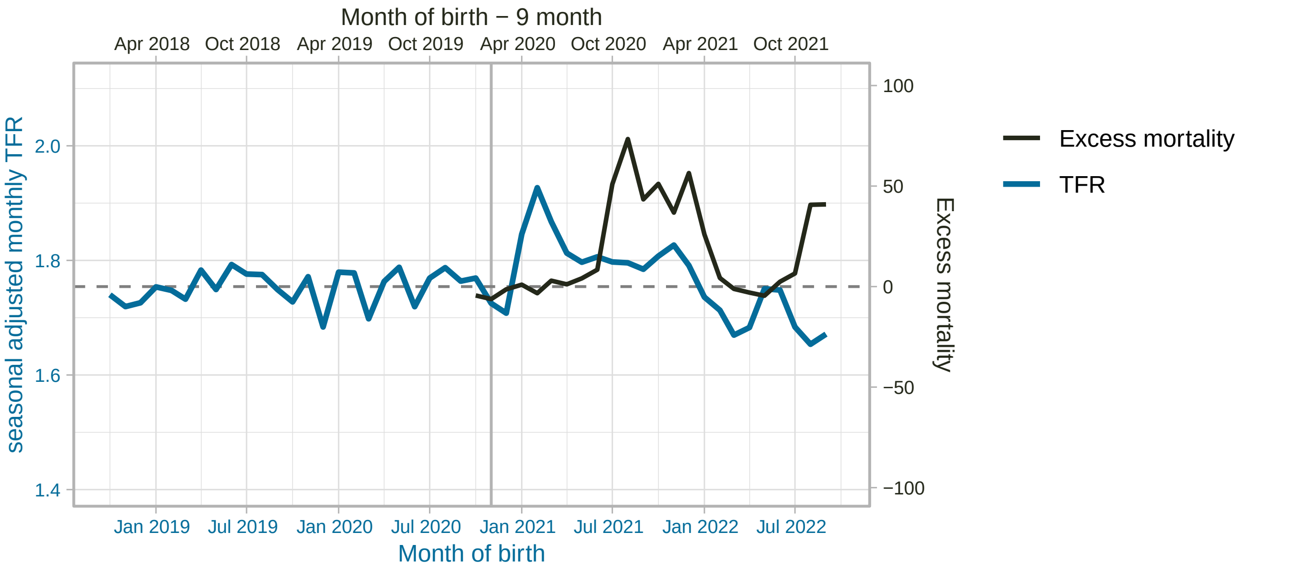

(figure continued on next page)

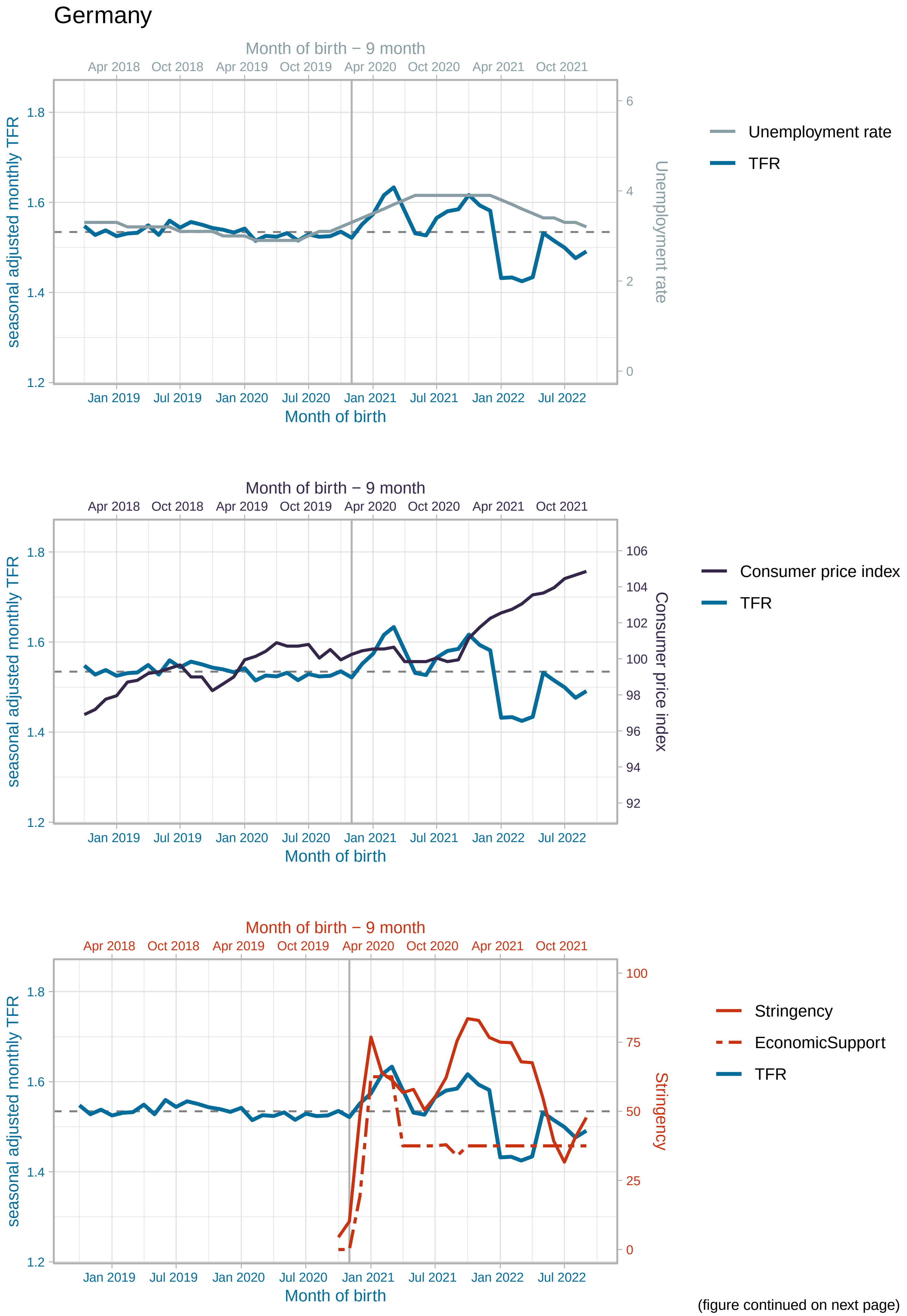

(figure continued on next page)

Germany

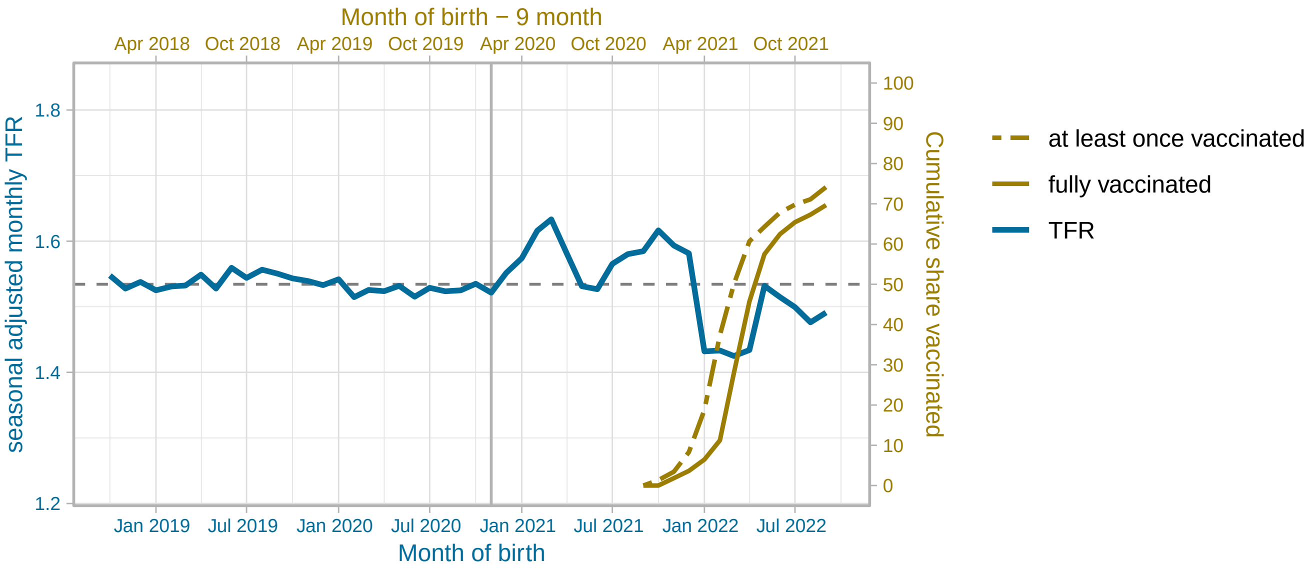

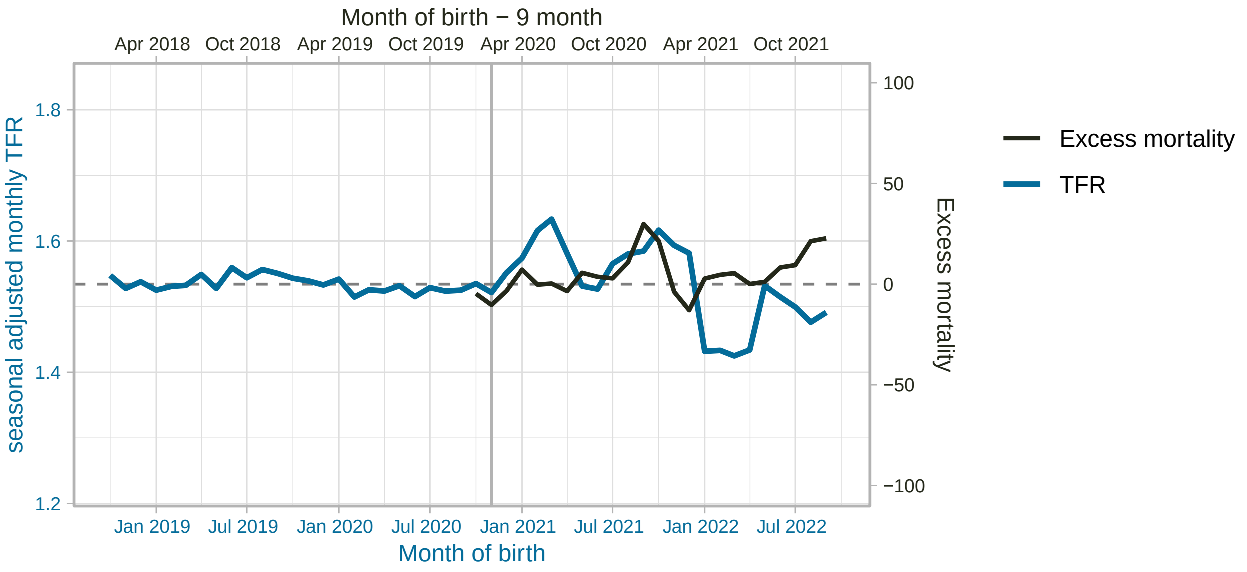

(figure continued on next page)

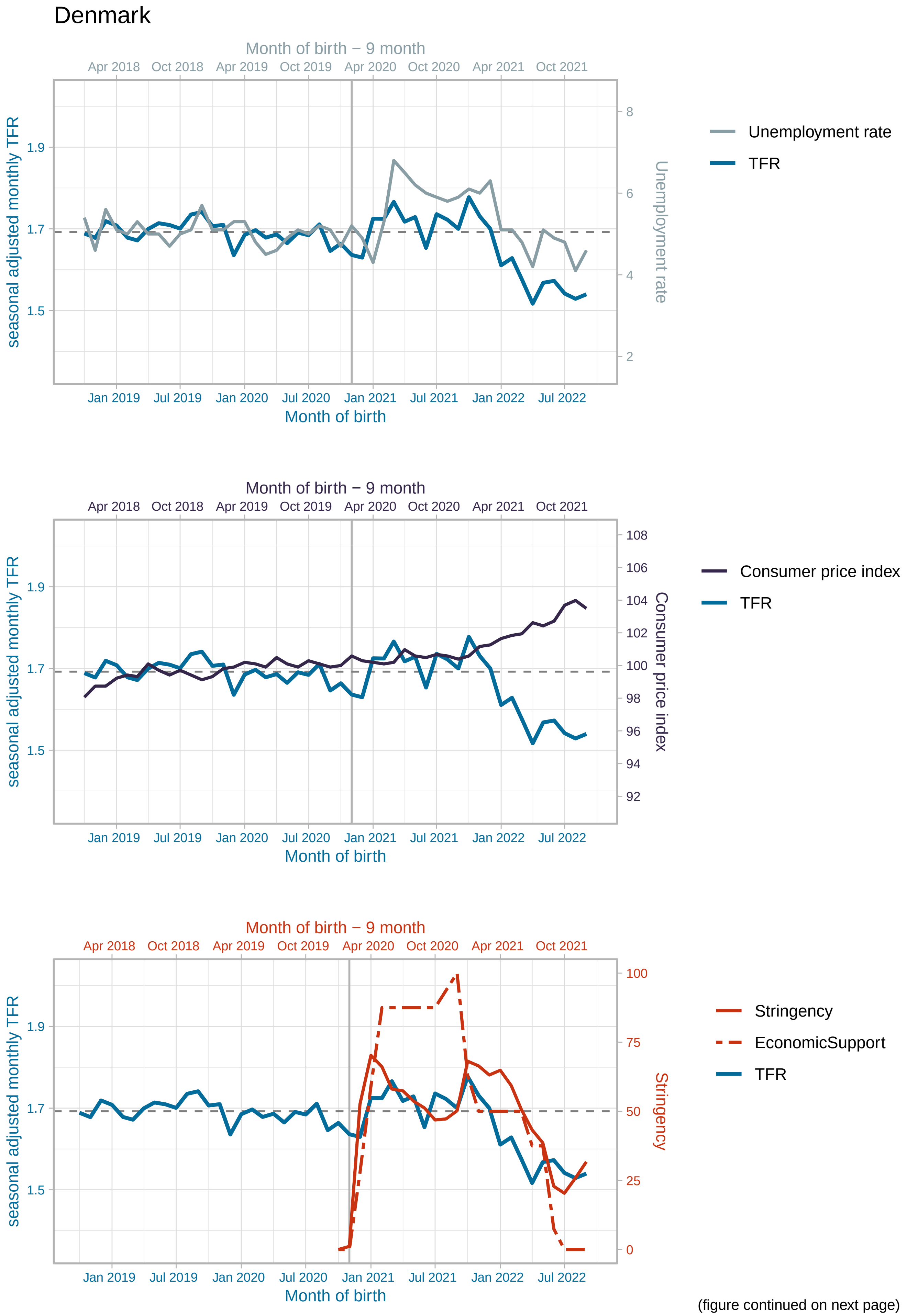

(figure continued on next page)

Denmark

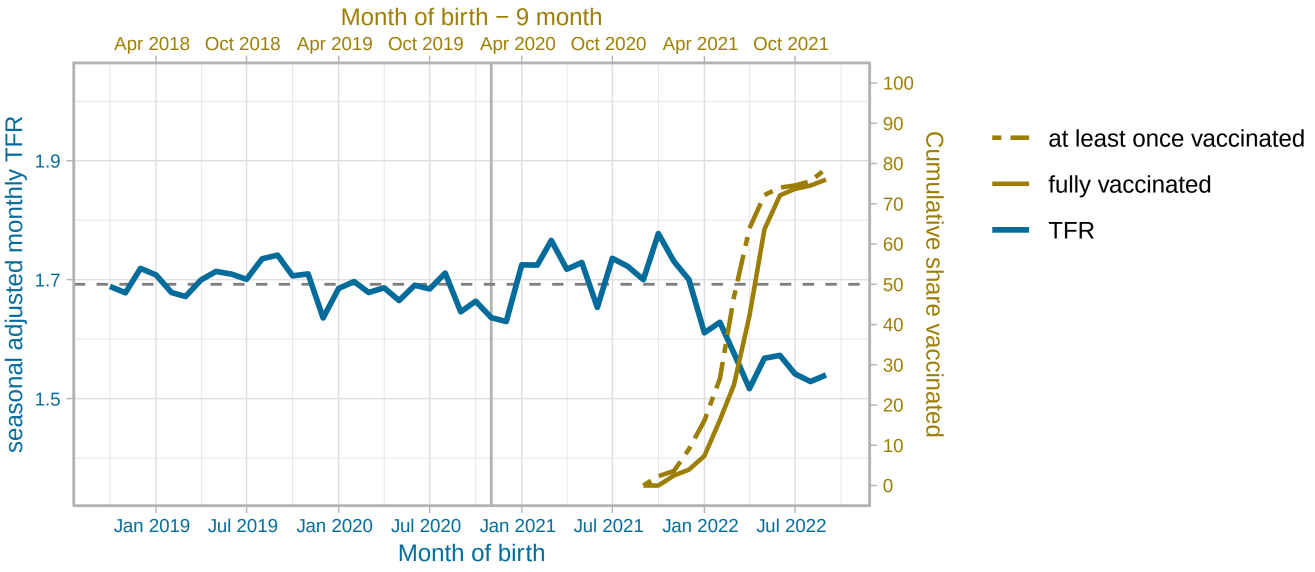

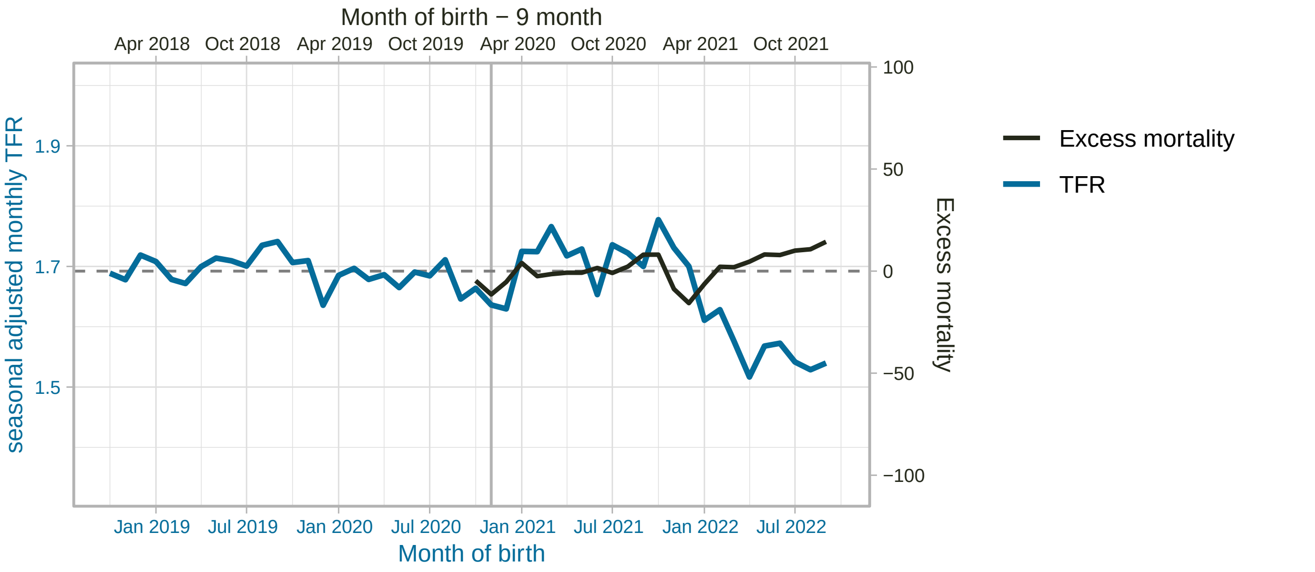

(figure continued on next page)

Spain

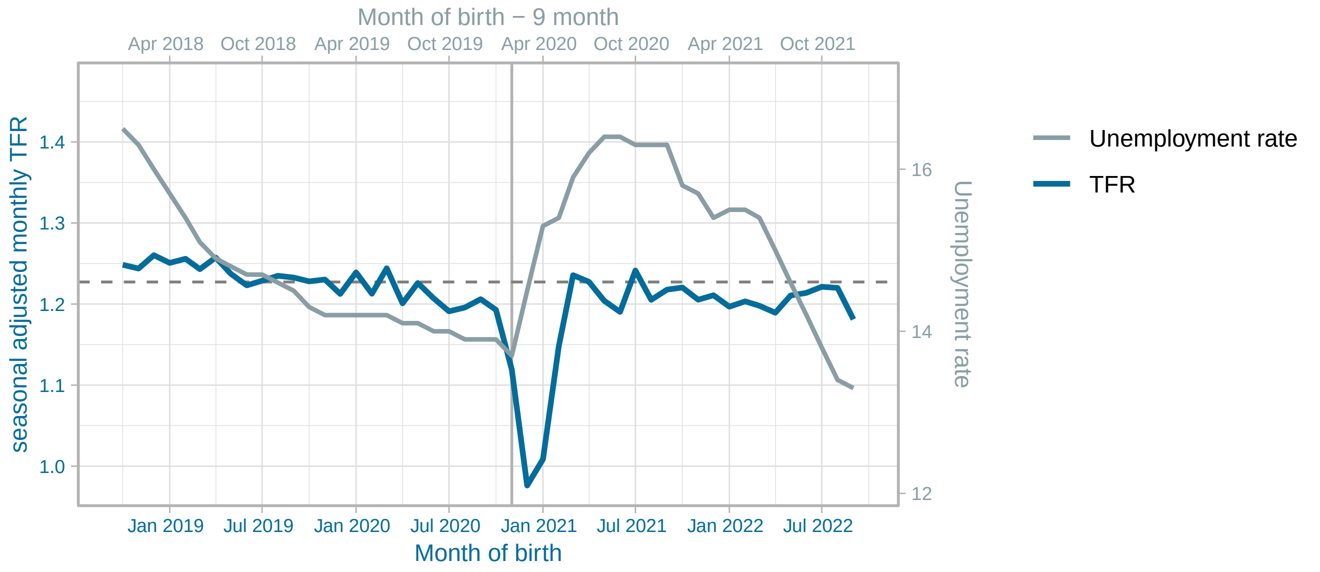

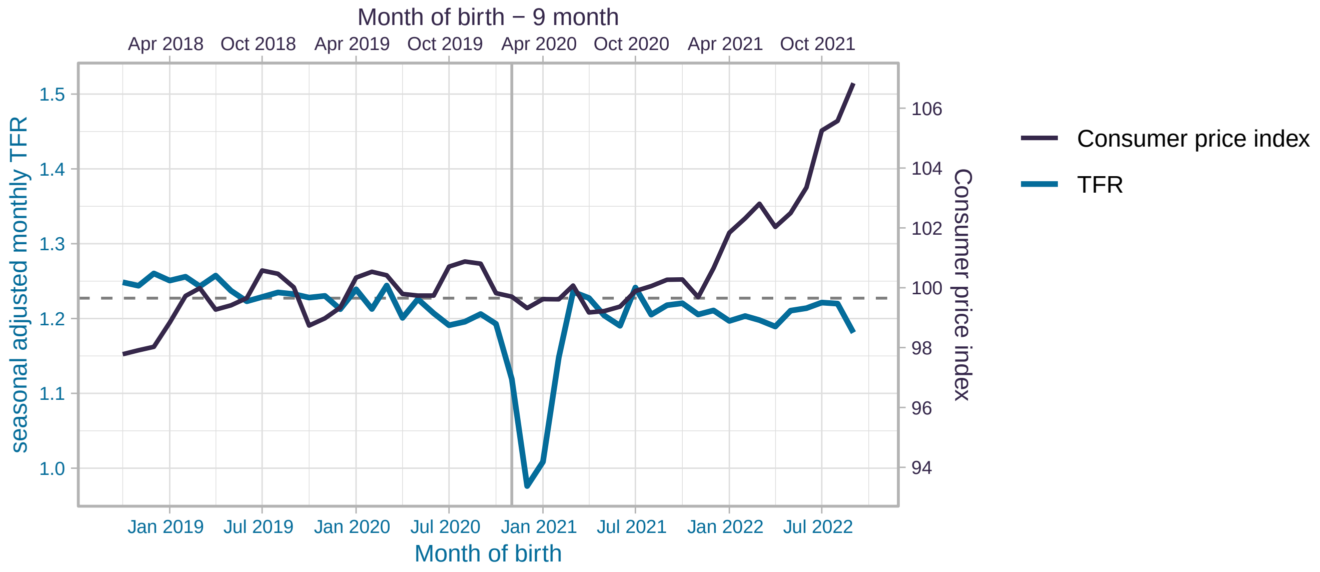

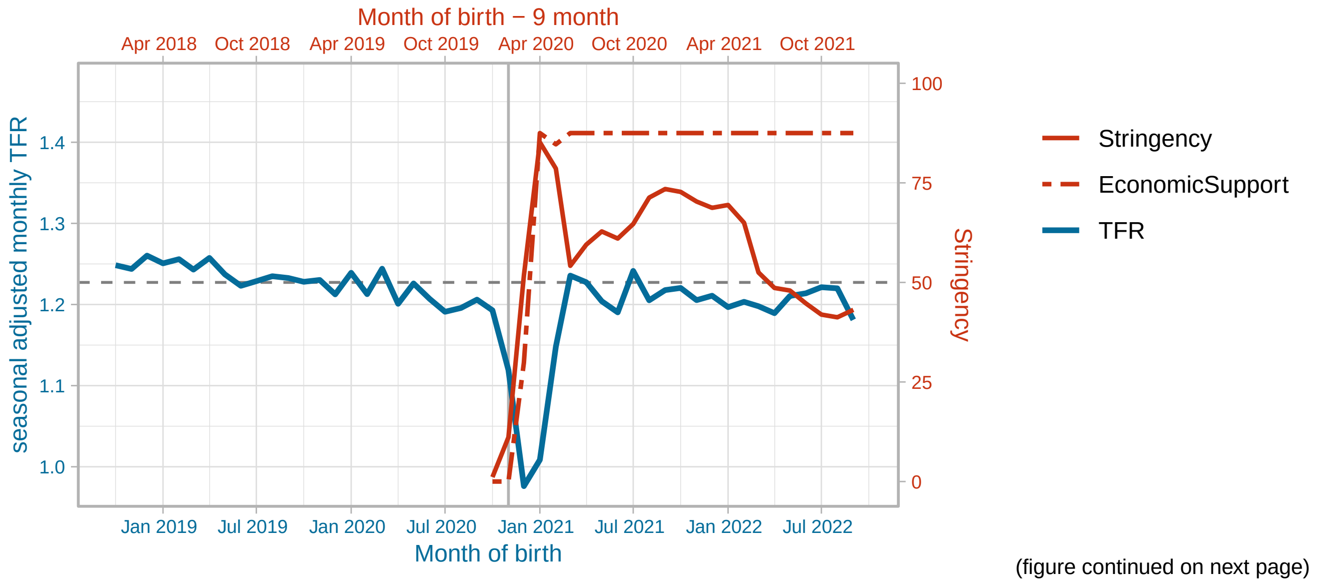

(figure continued on next page)

Spain

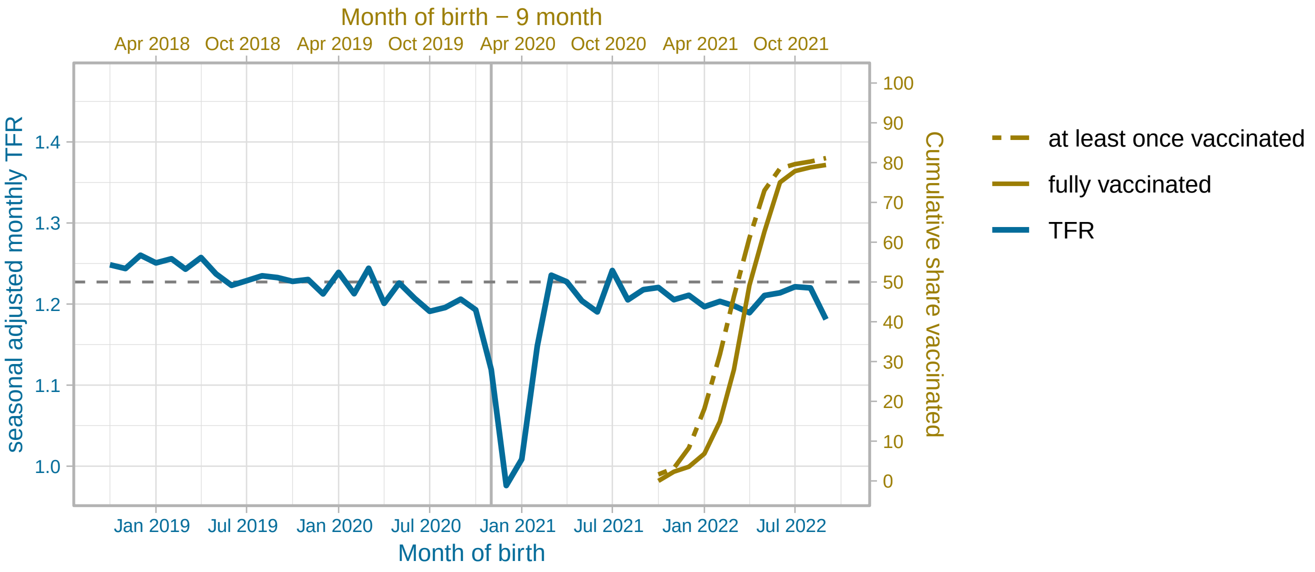

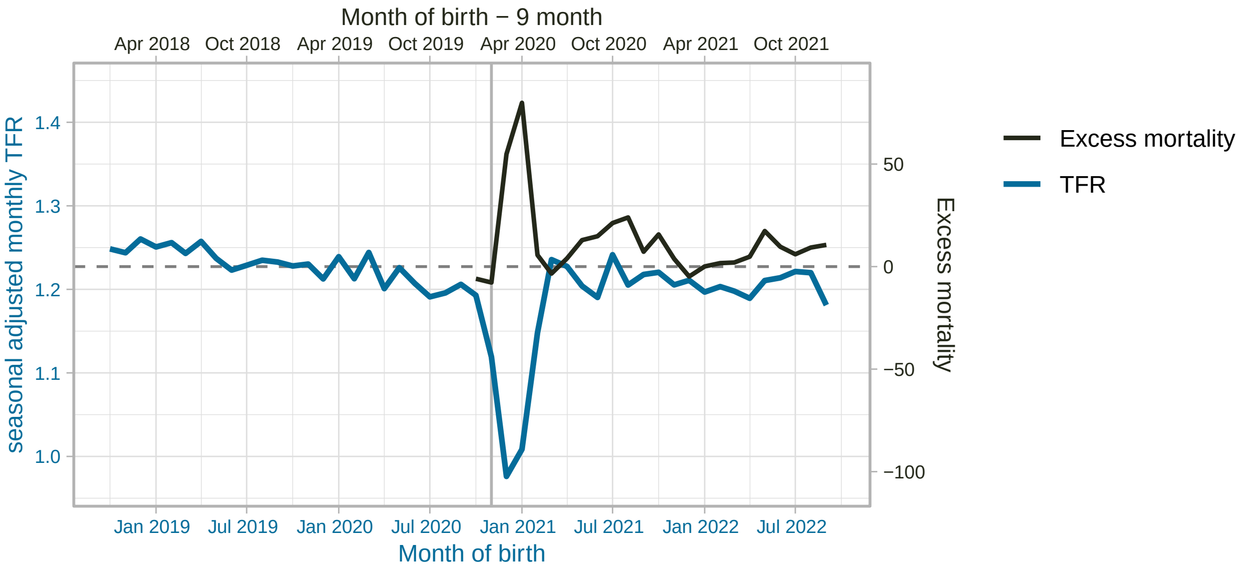

(figure continued on next page)

Finland

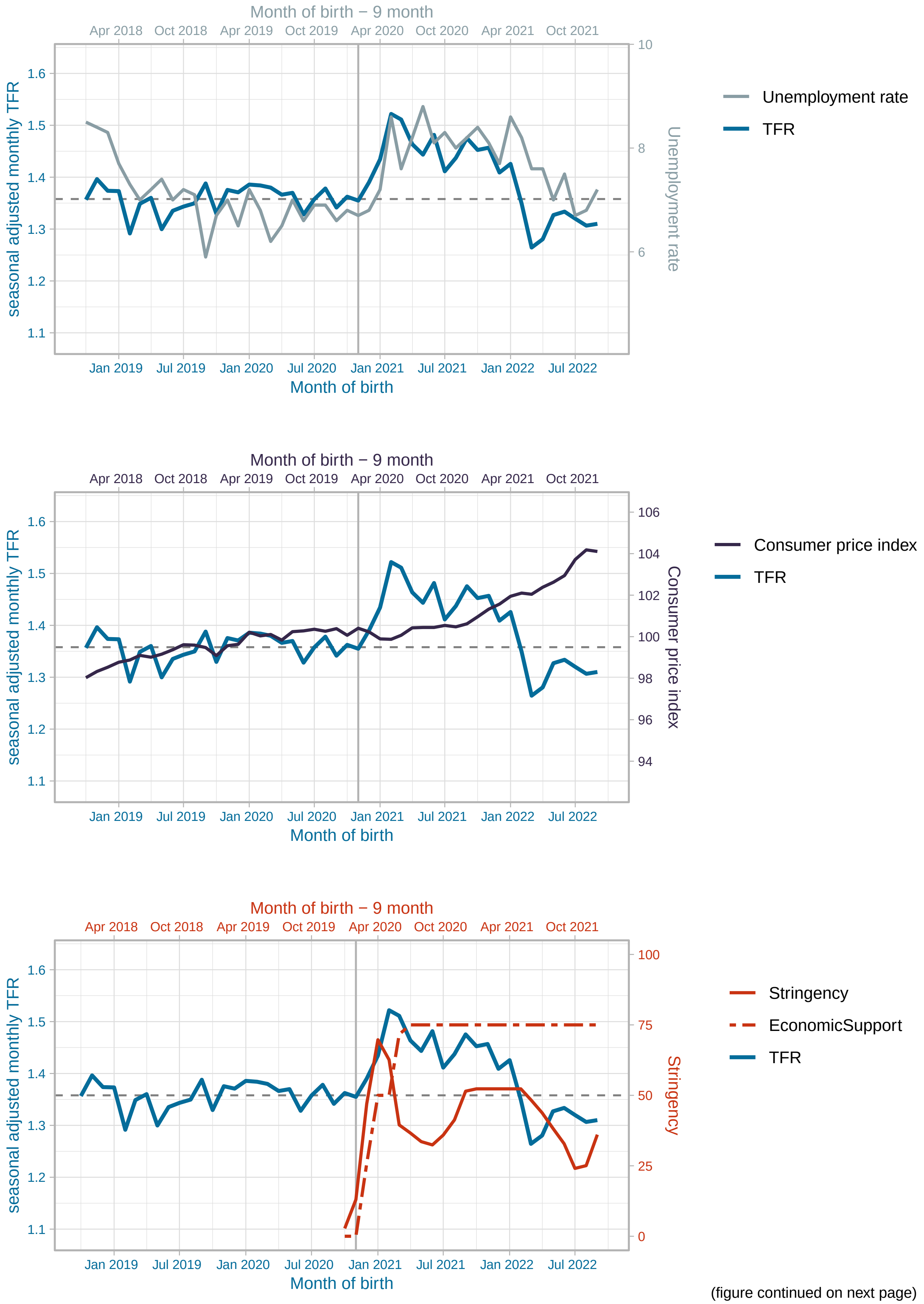

(figure continued on next page)

Finland

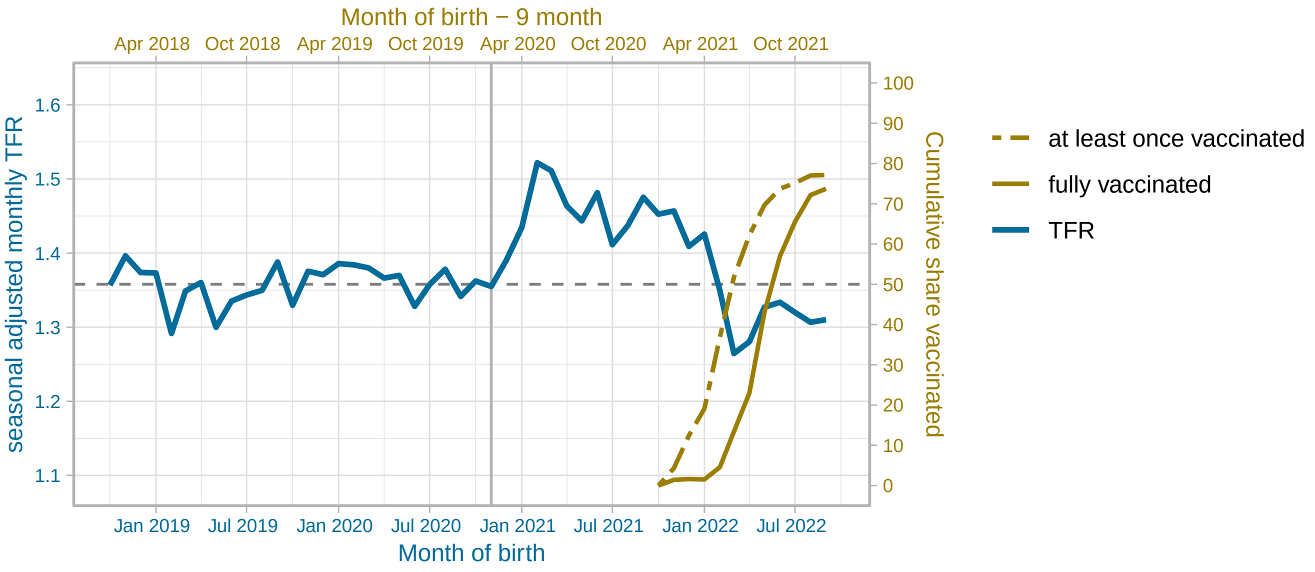

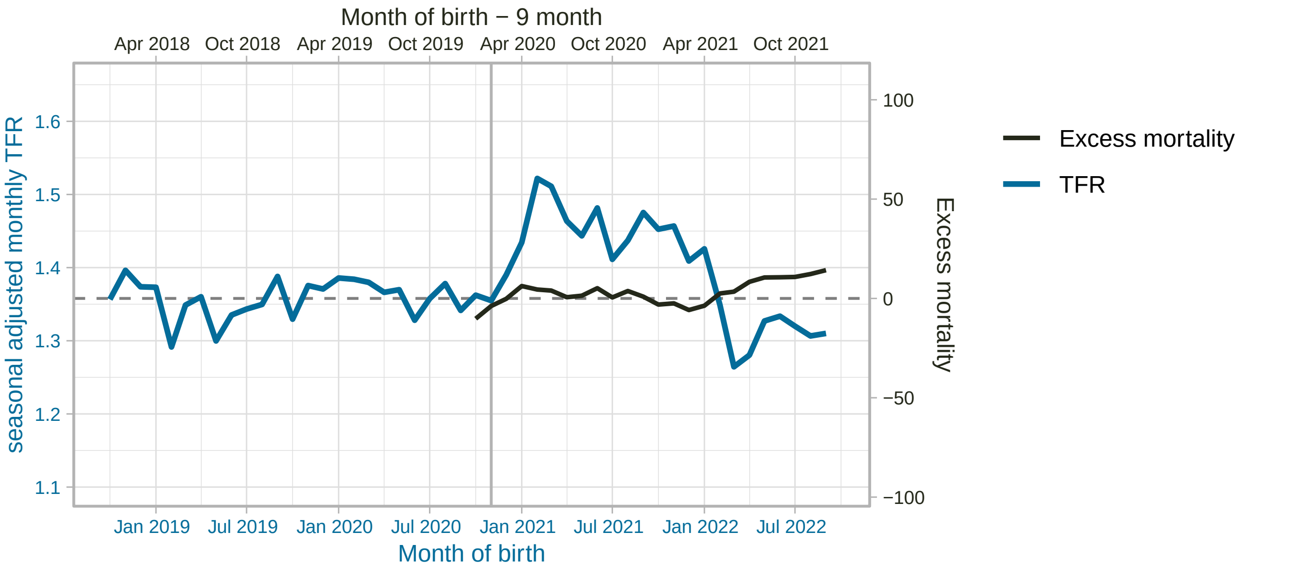

(figure continued on next page)

France

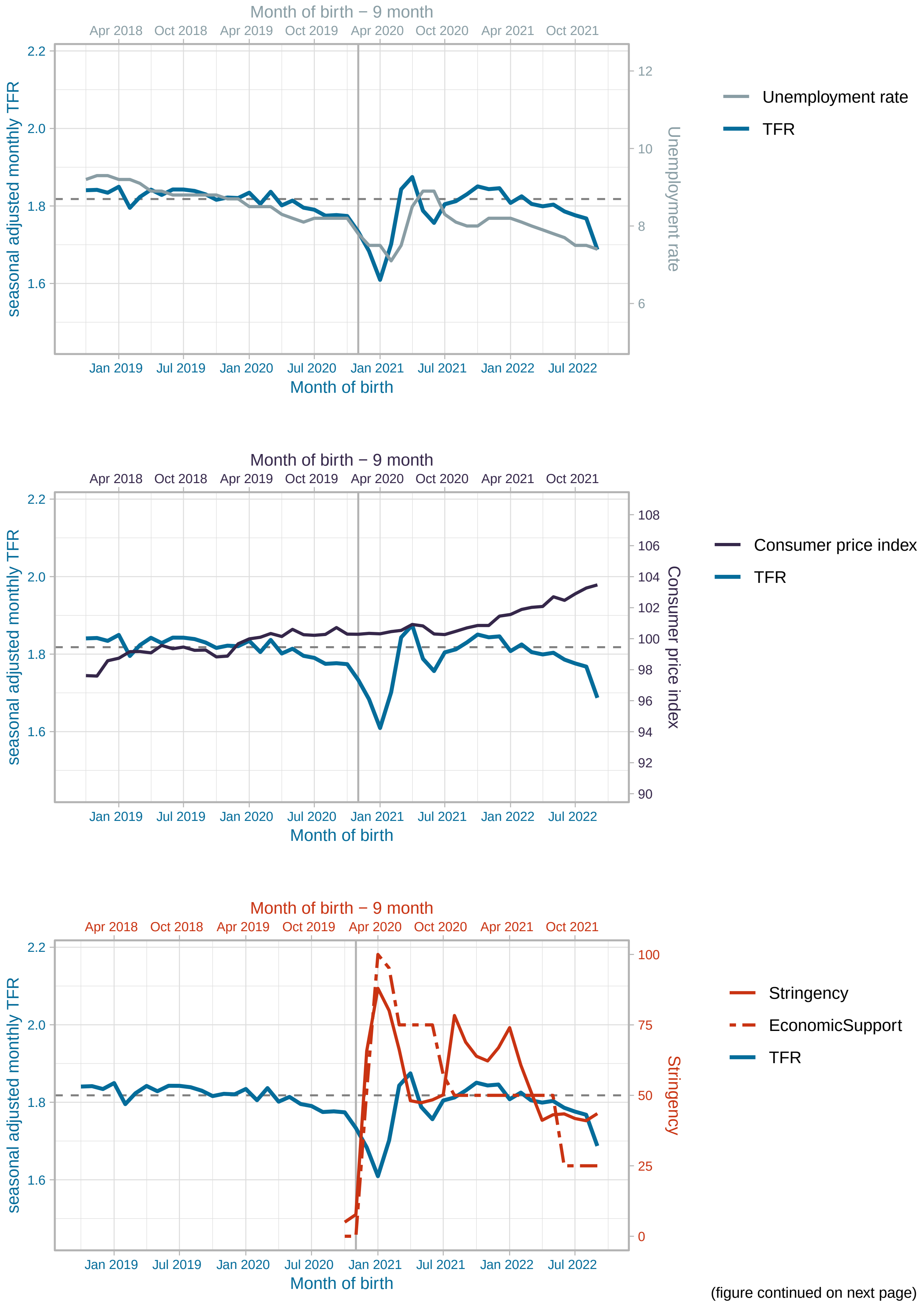

(figure continued on next page)

France

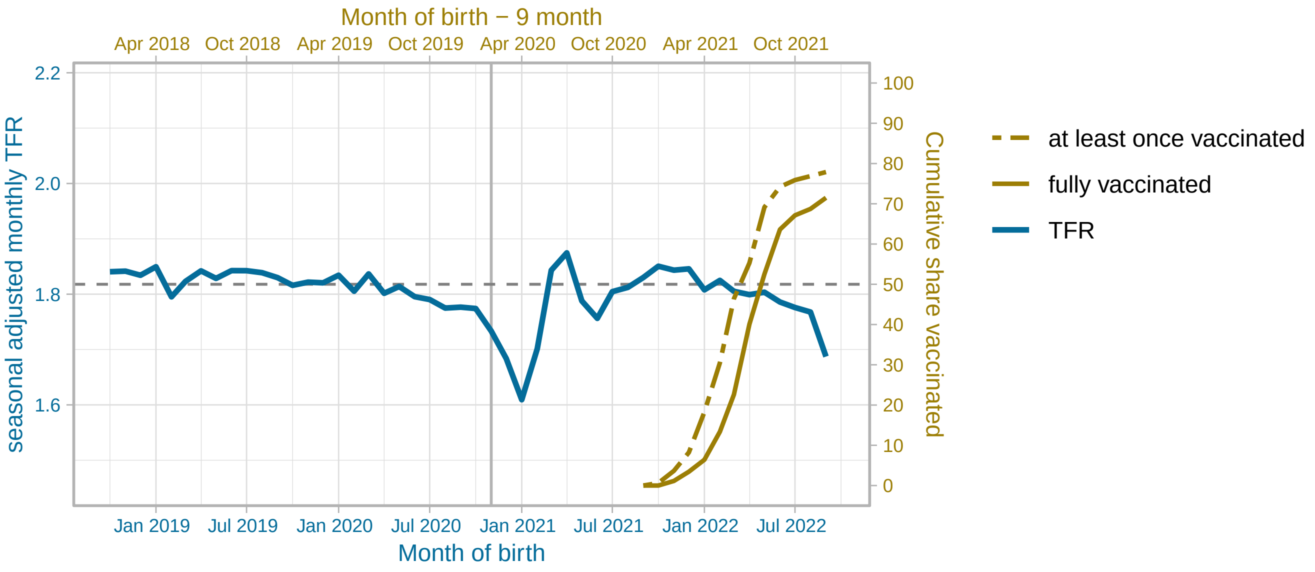

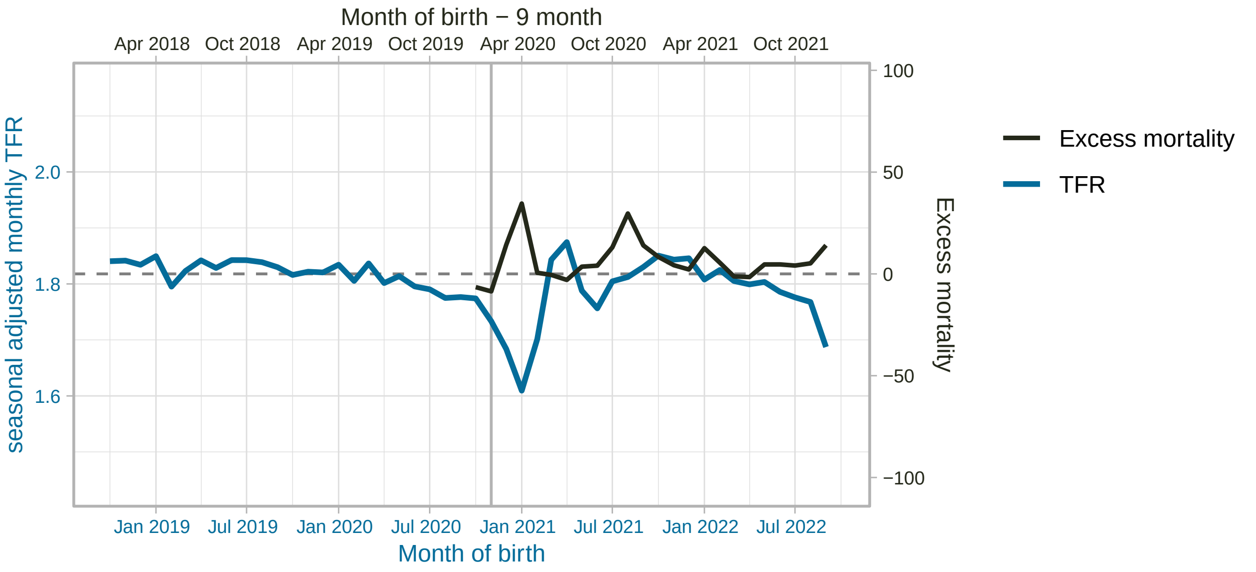

(figure continued on next page)

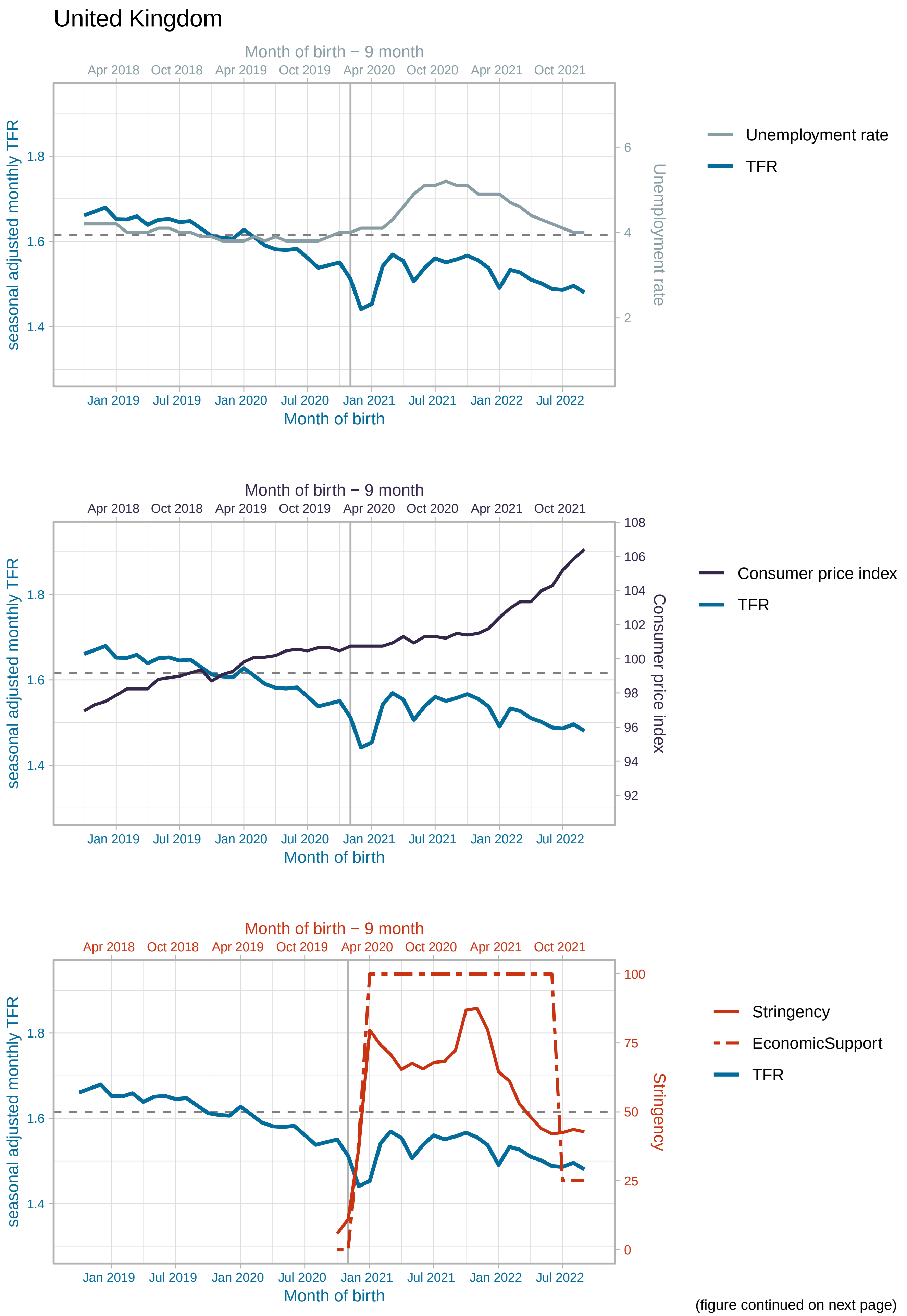

(figure continued on next page)

United Kingdom

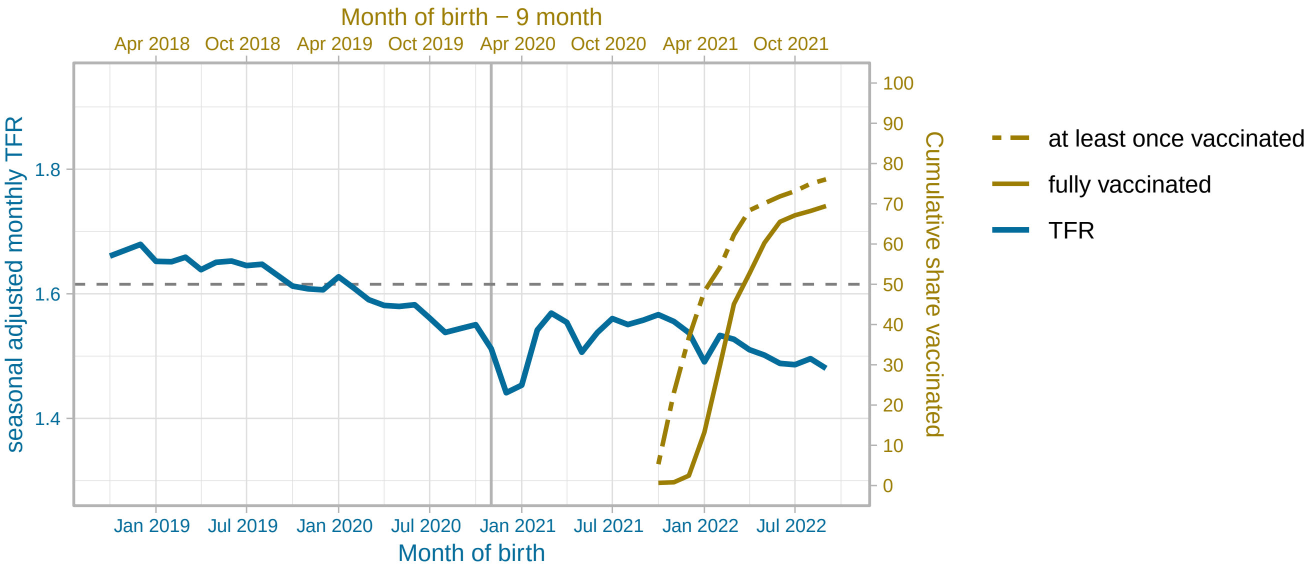

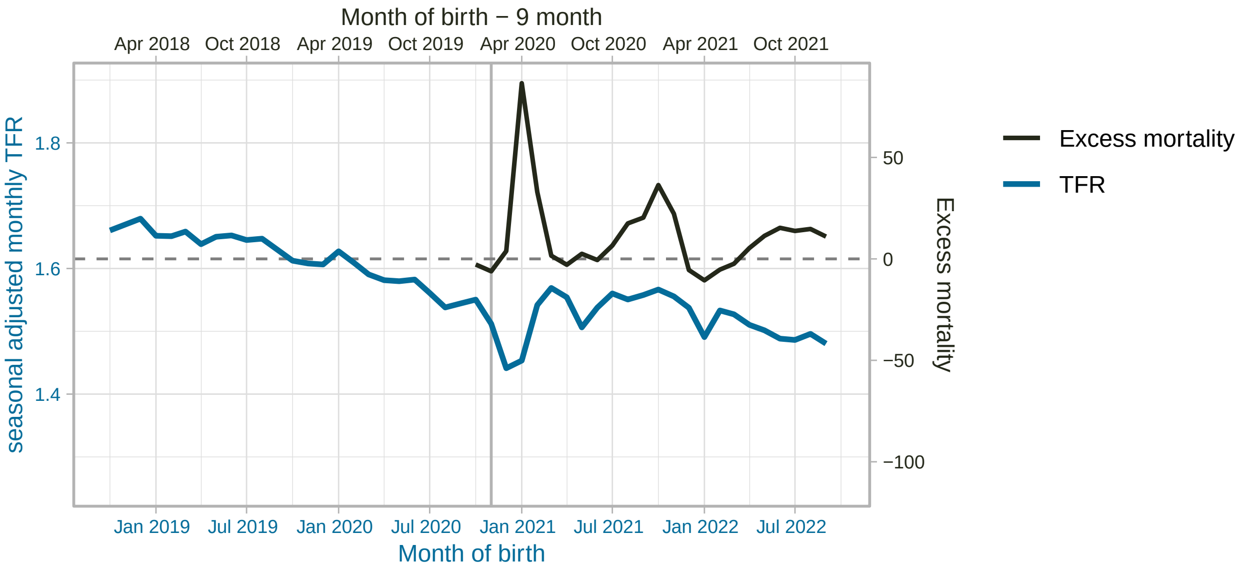

(figure continued on next page)

Greece

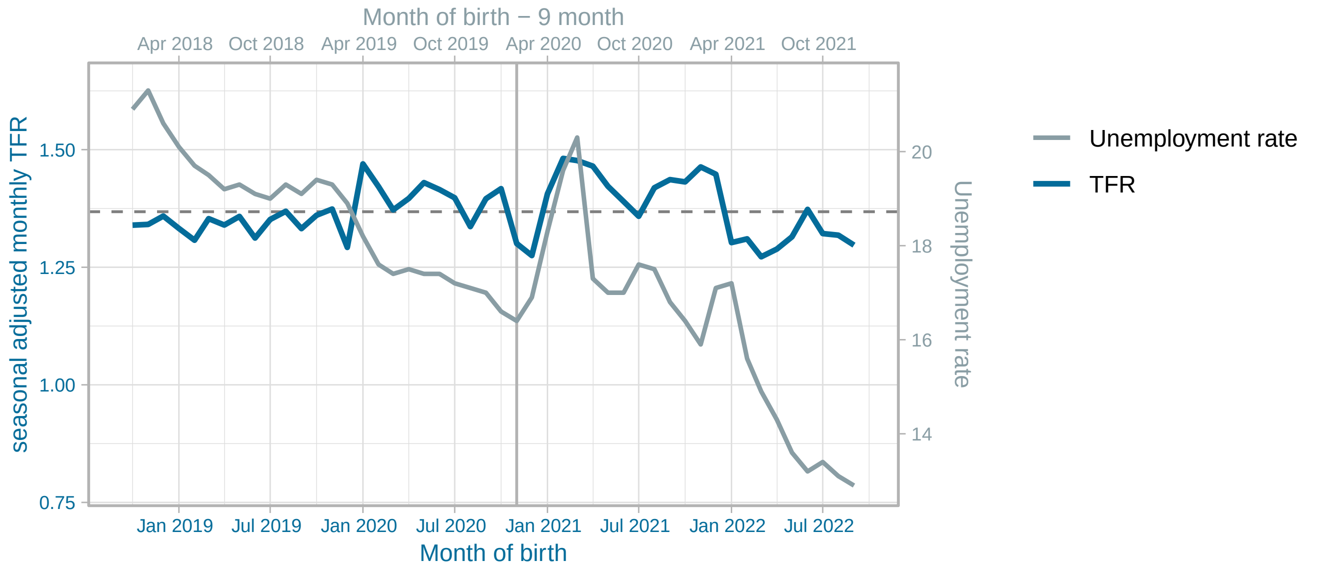

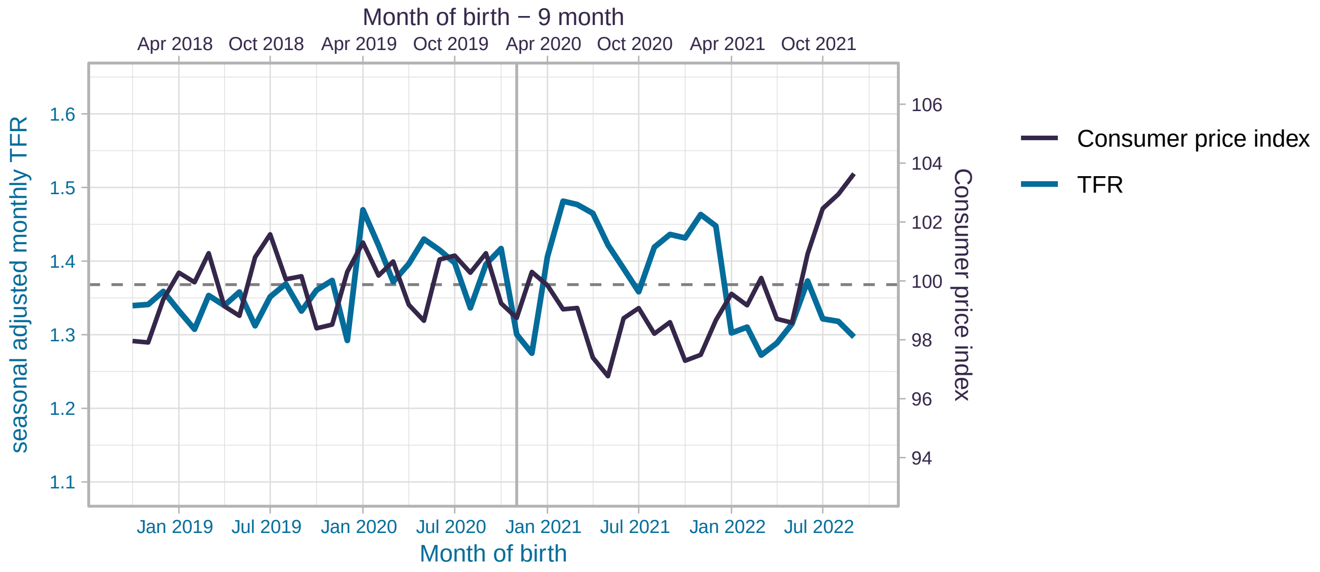

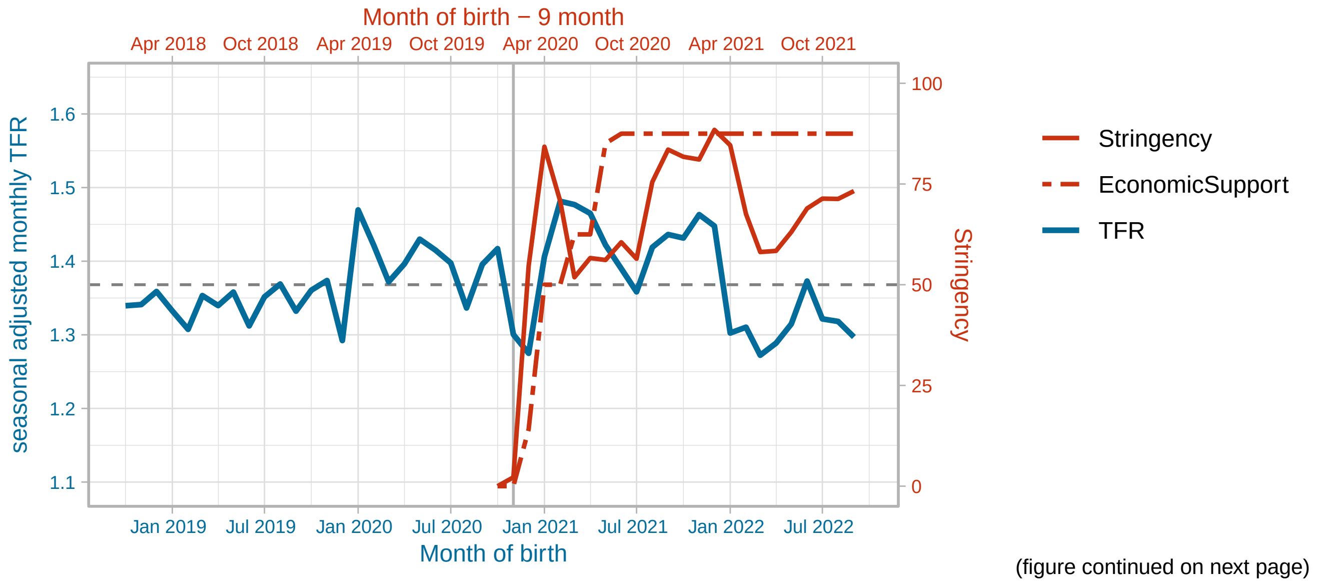

(figure continued on next page)

Greece

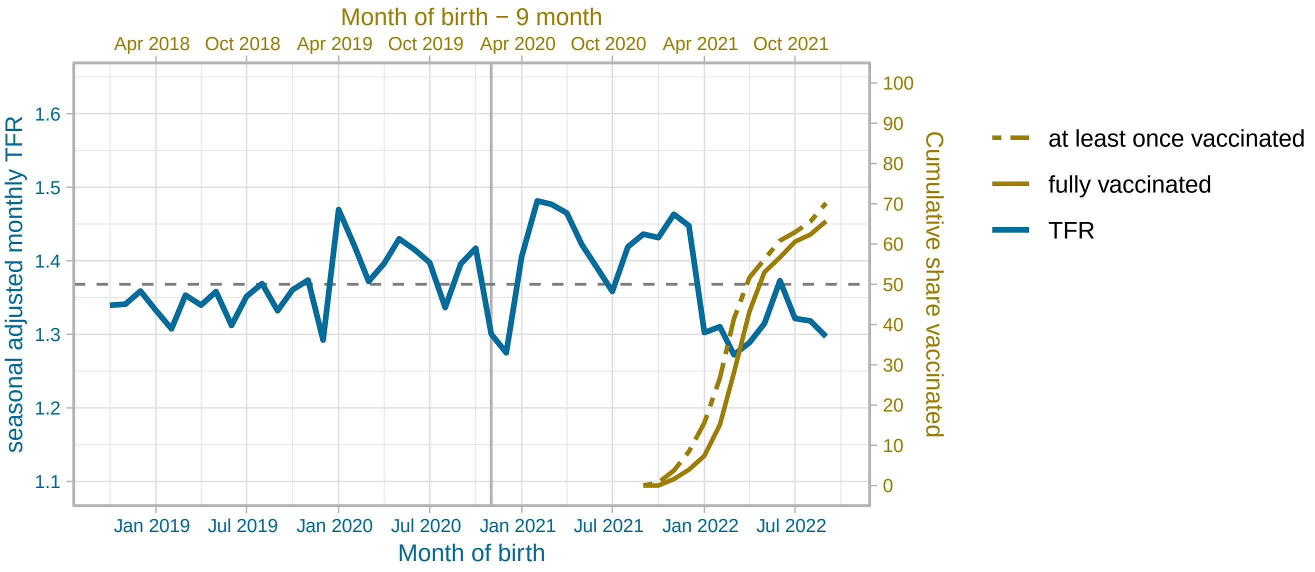

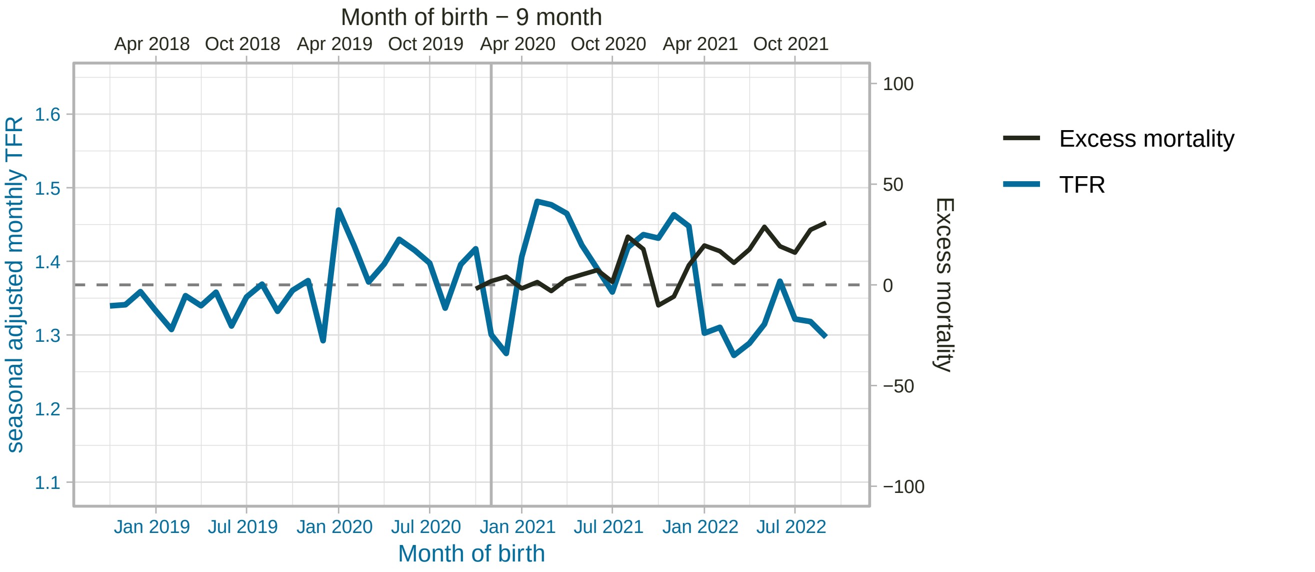

(figure continued on next page)

Hungary

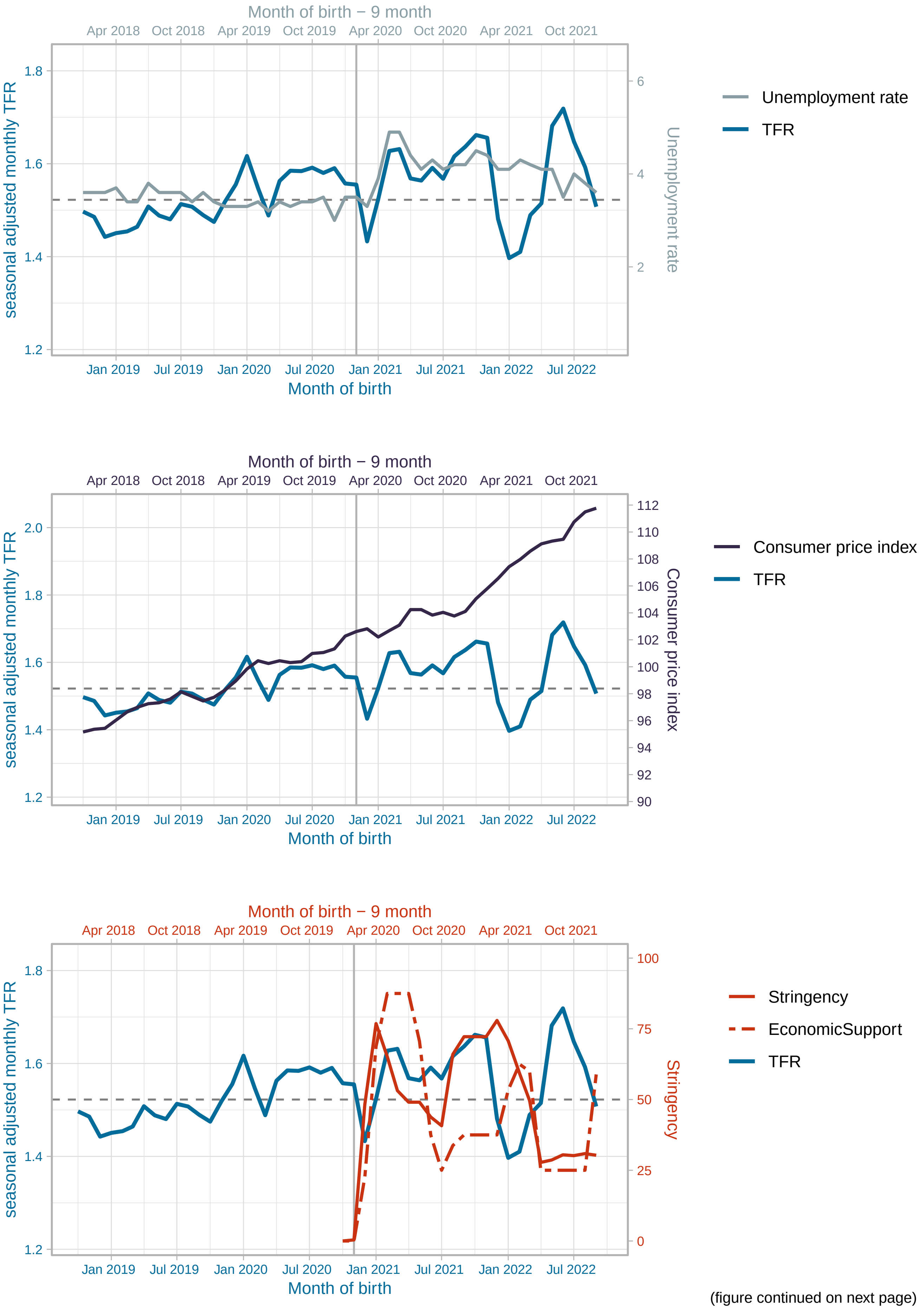

(figure continued on next page)

Hungary

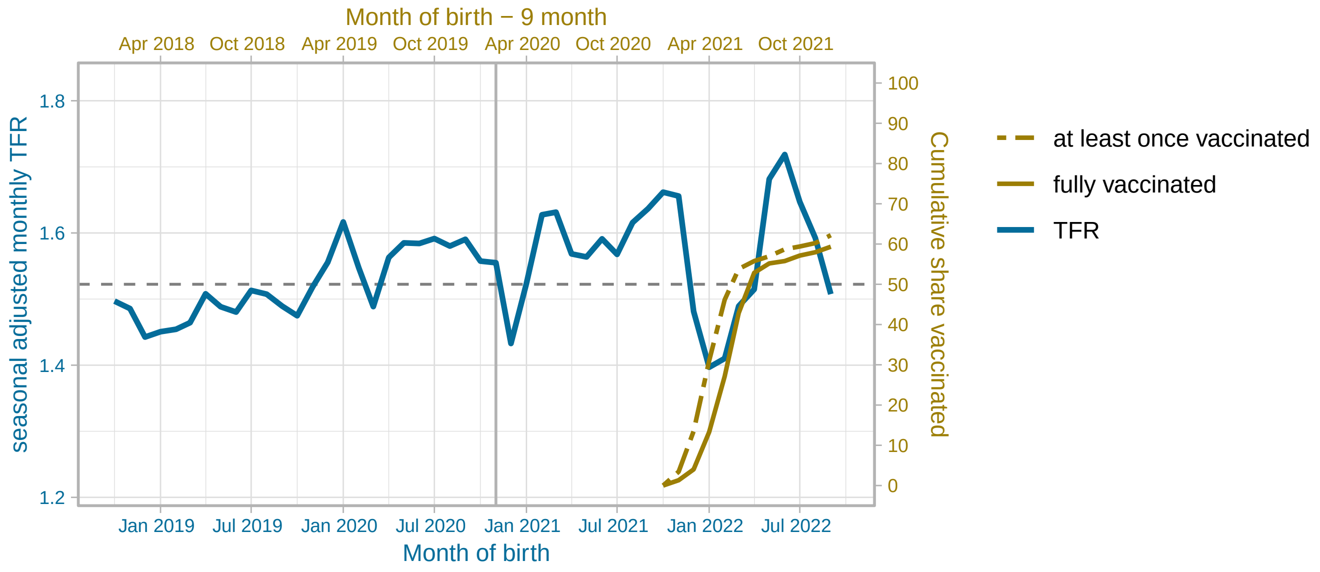

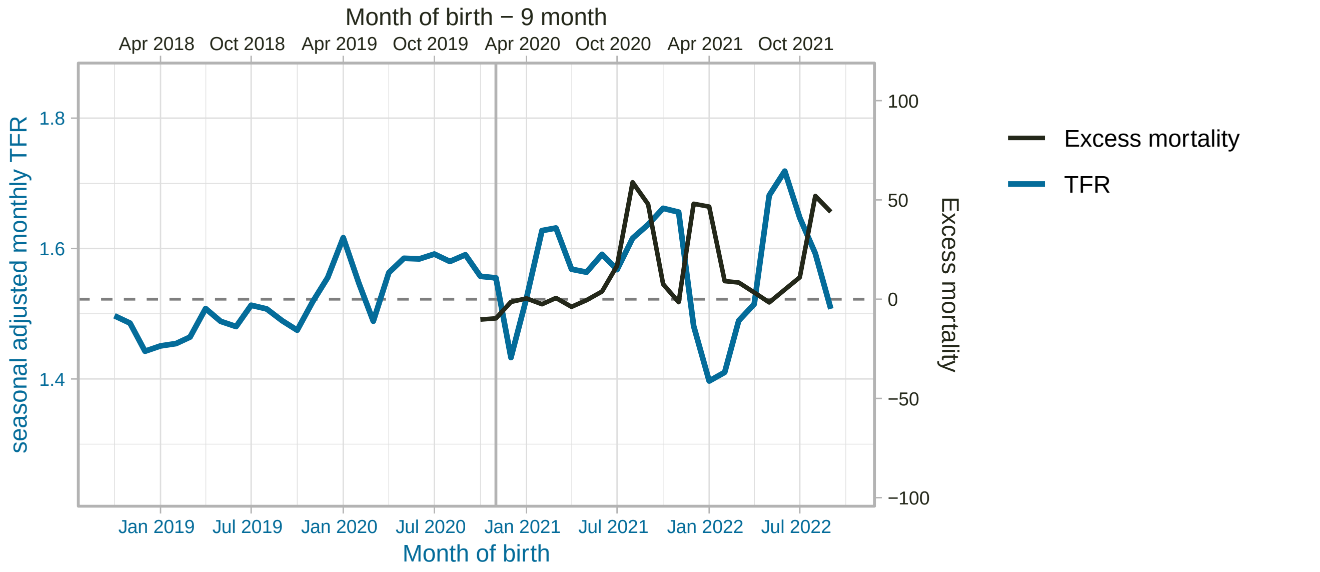

(figure continued on next page)

Ireland

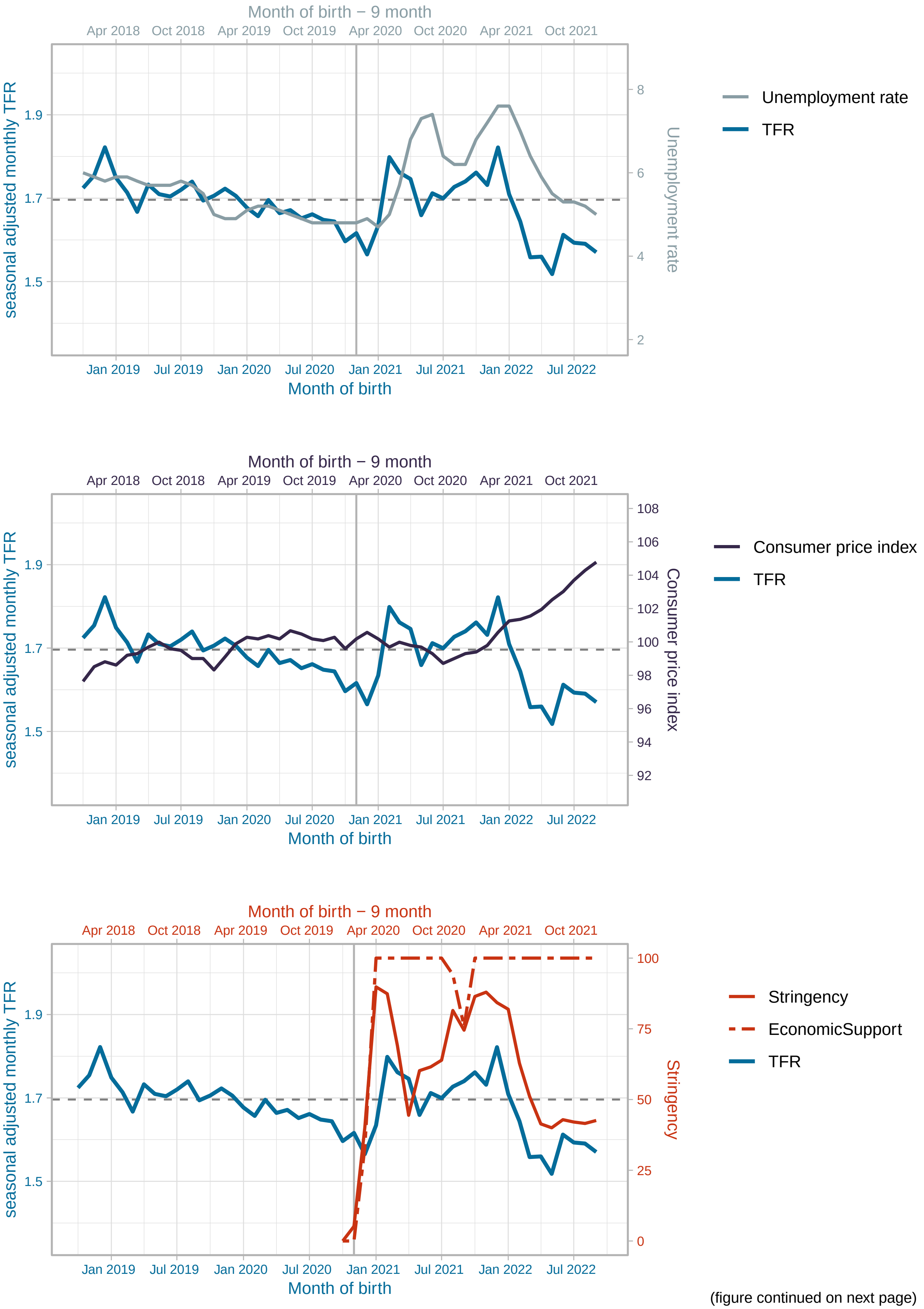

(figure continued on next page)

Ireland

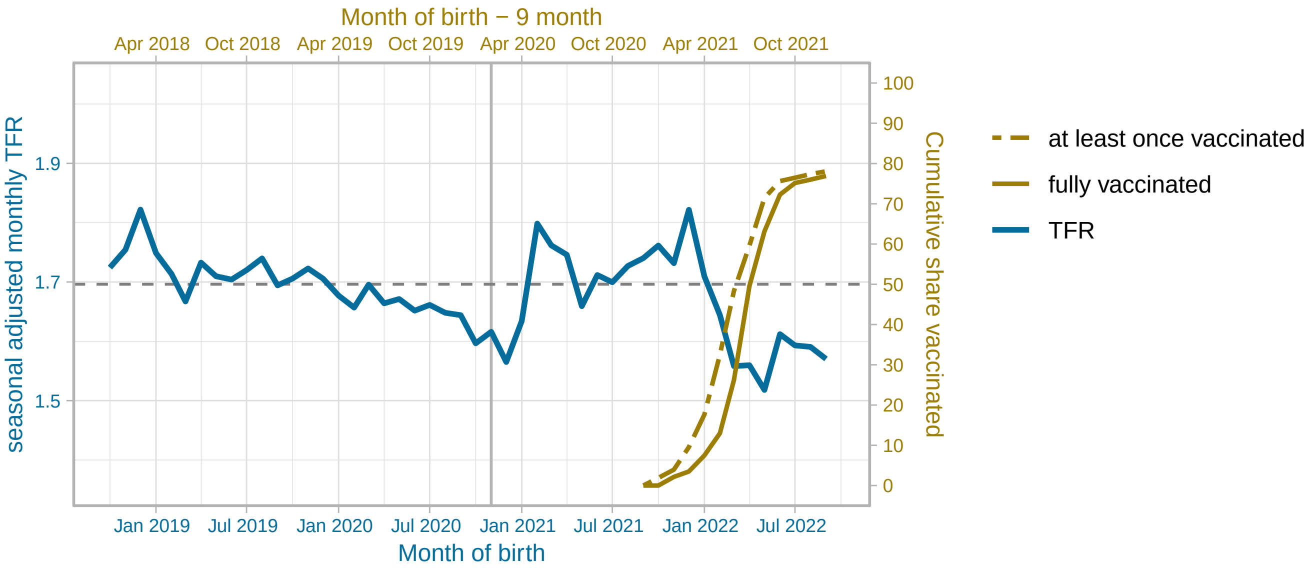

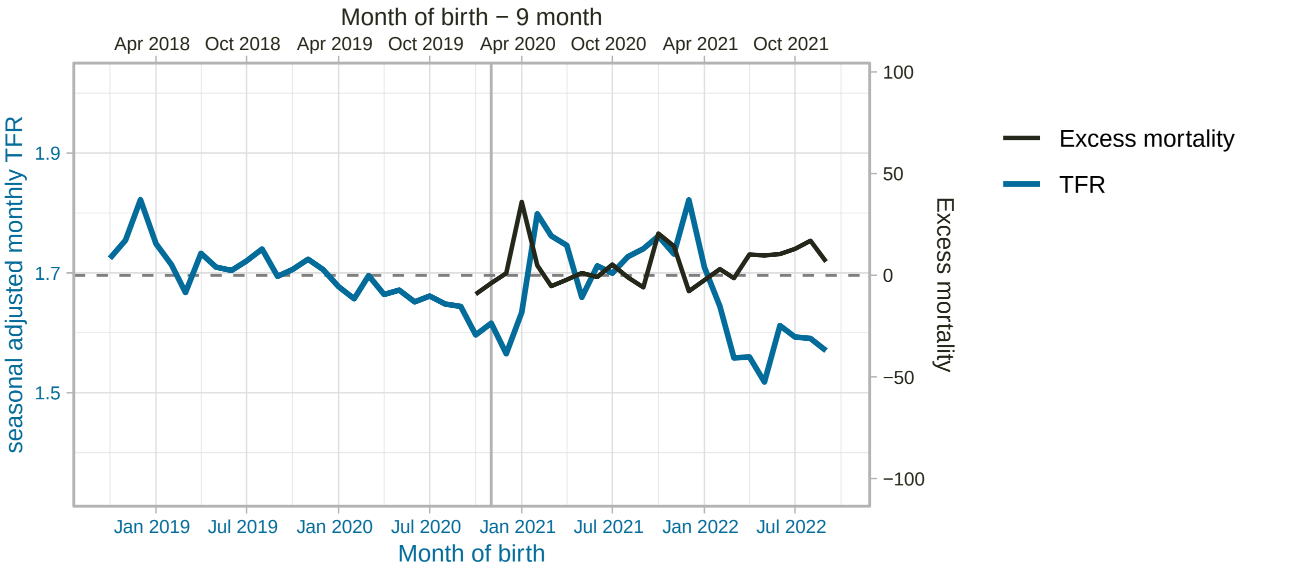

(figure continued on next page)

Israel

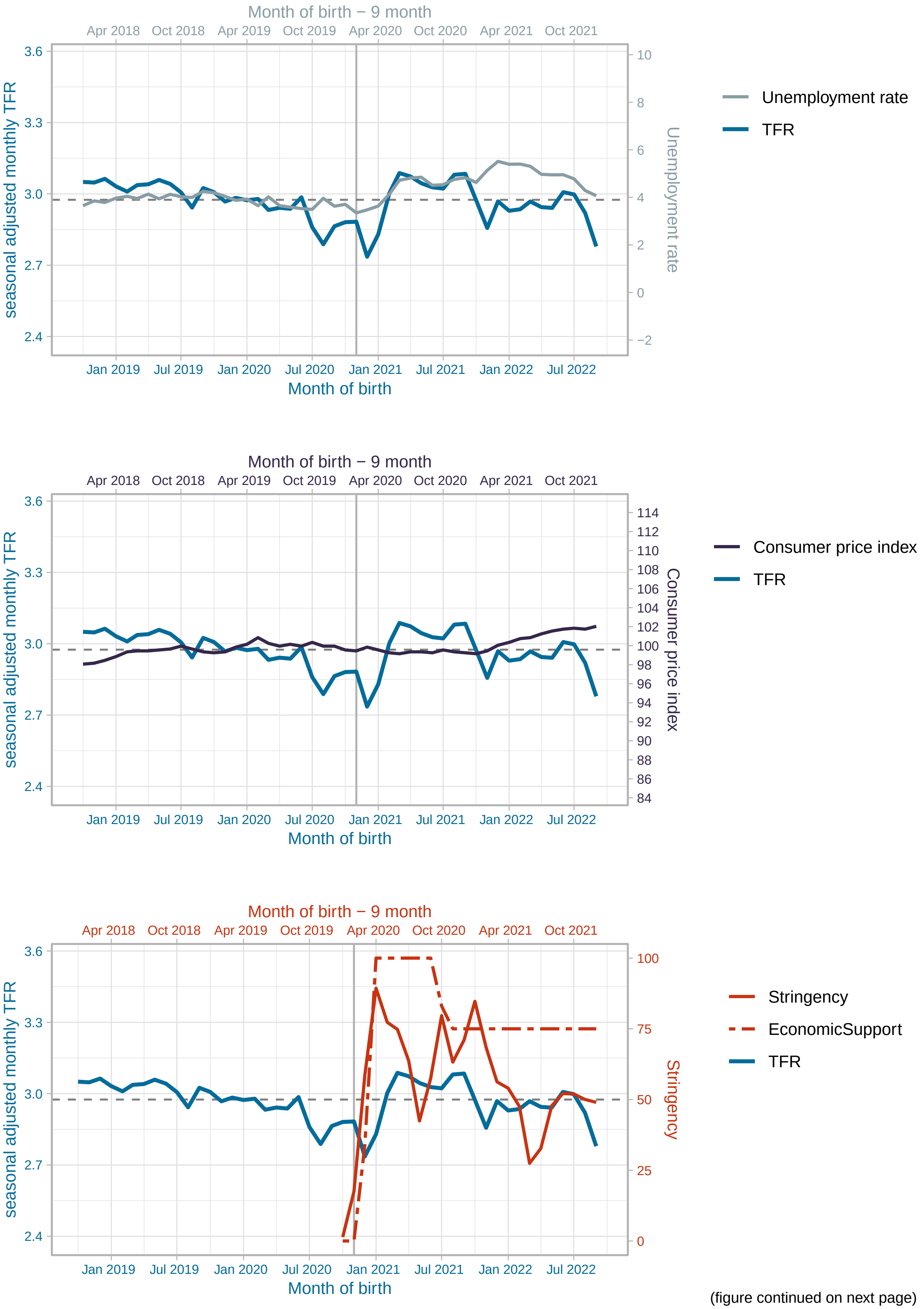

(figure continued on next page)

Israel

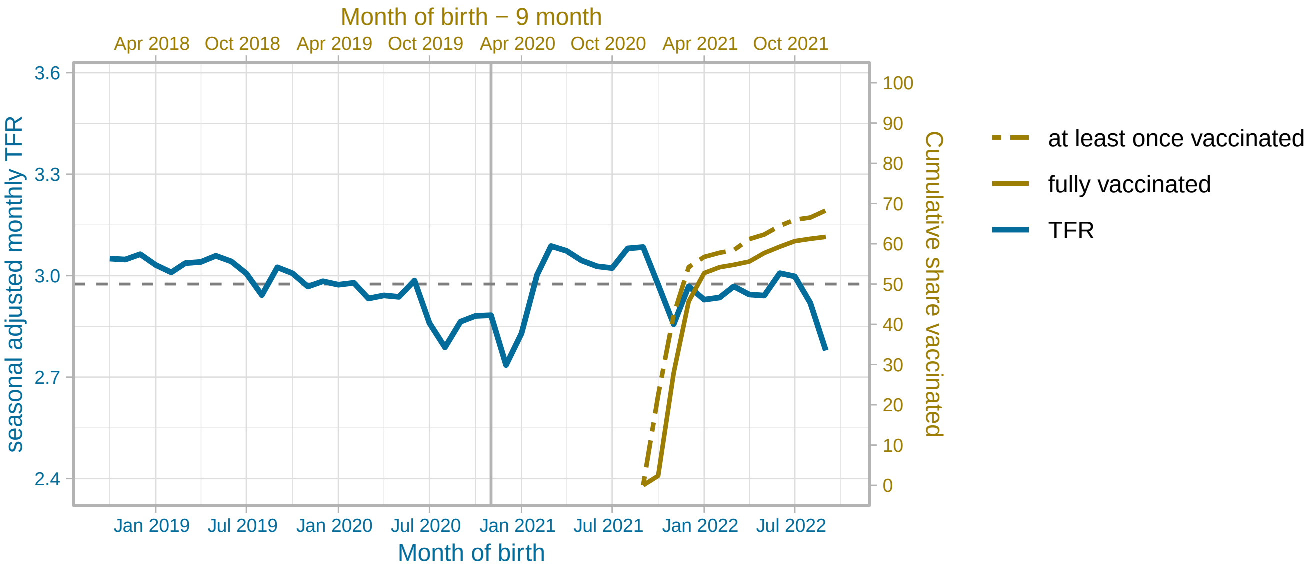

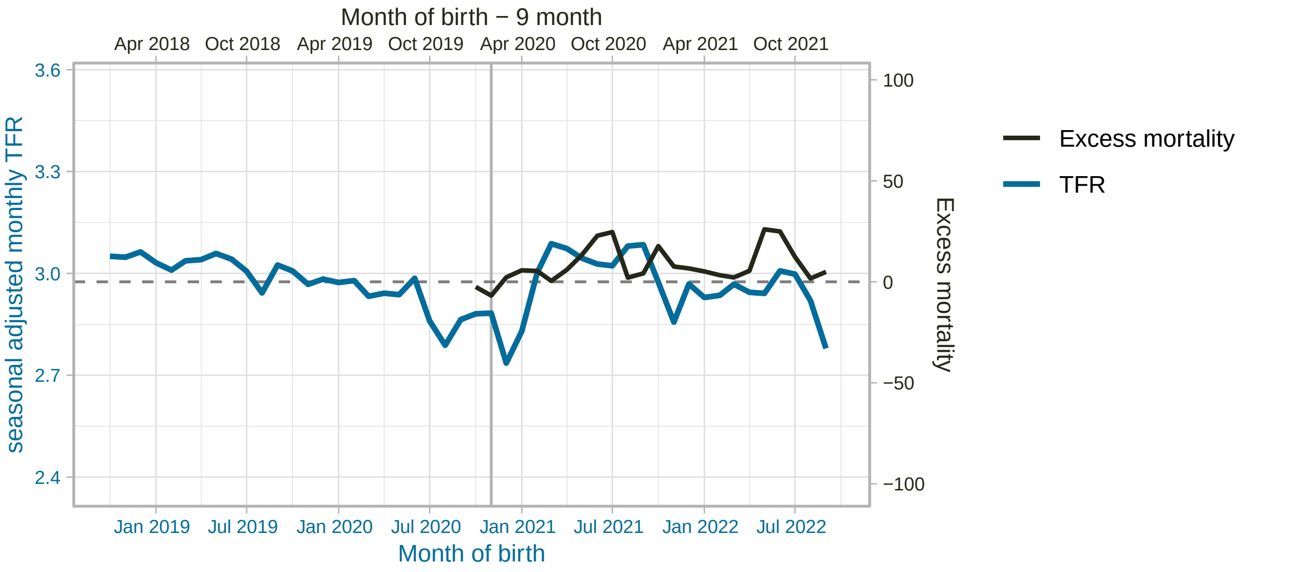

(figure continued on next page)

Italy

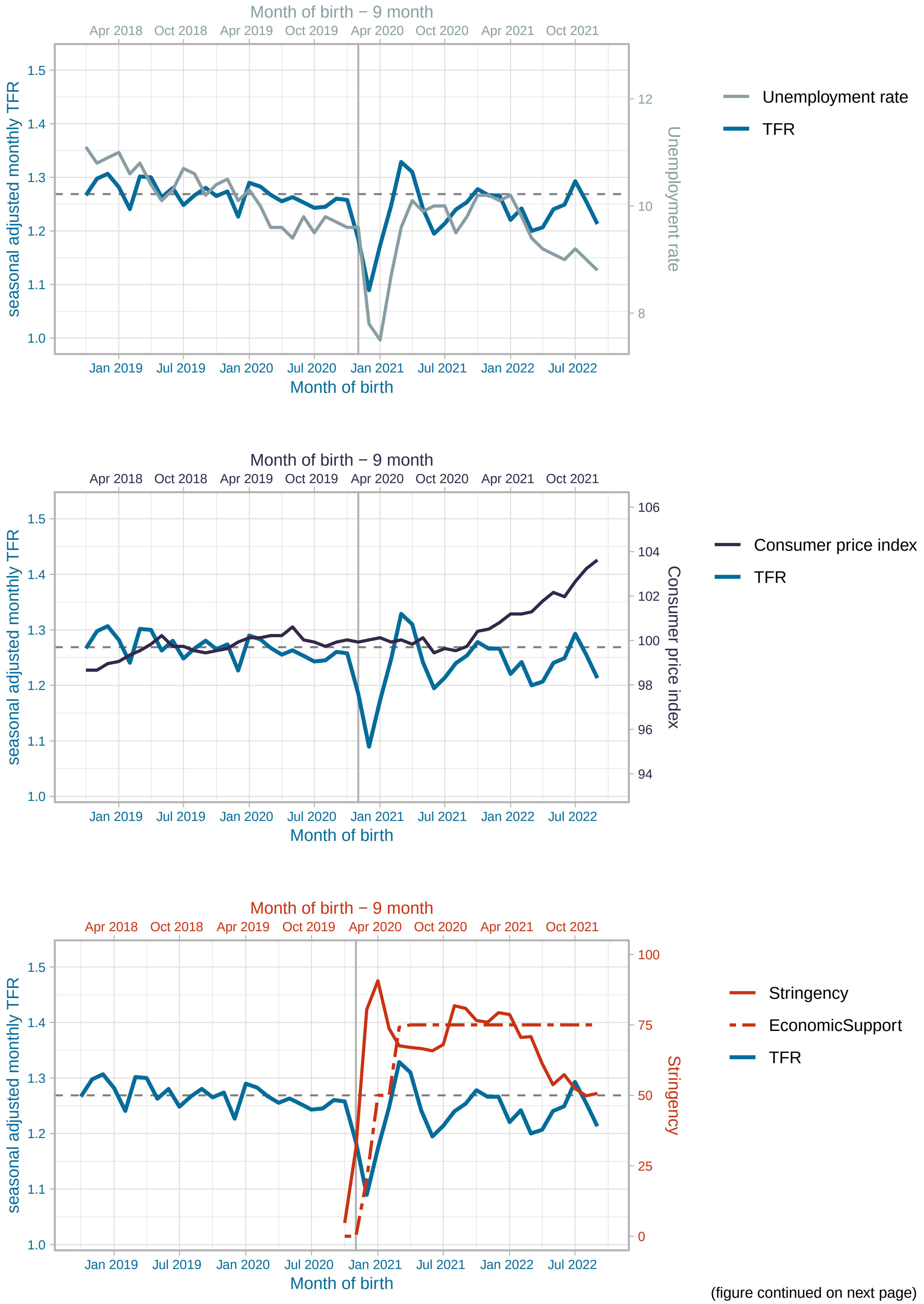

(figure continued on next page)

Italy

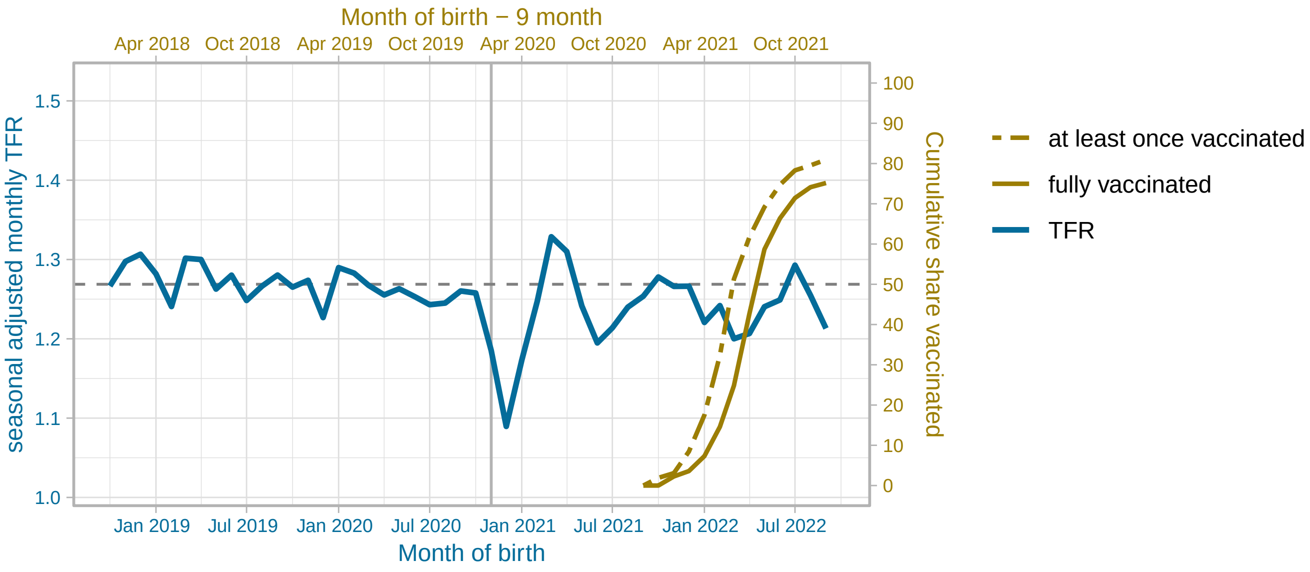

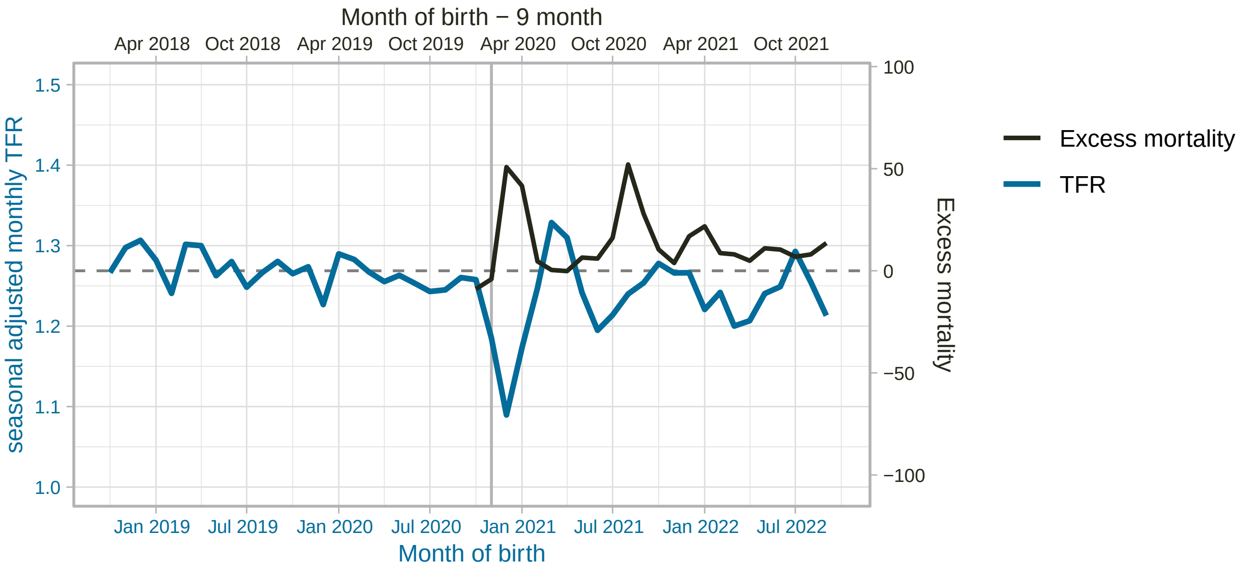

(figure continued on next page)

Japan

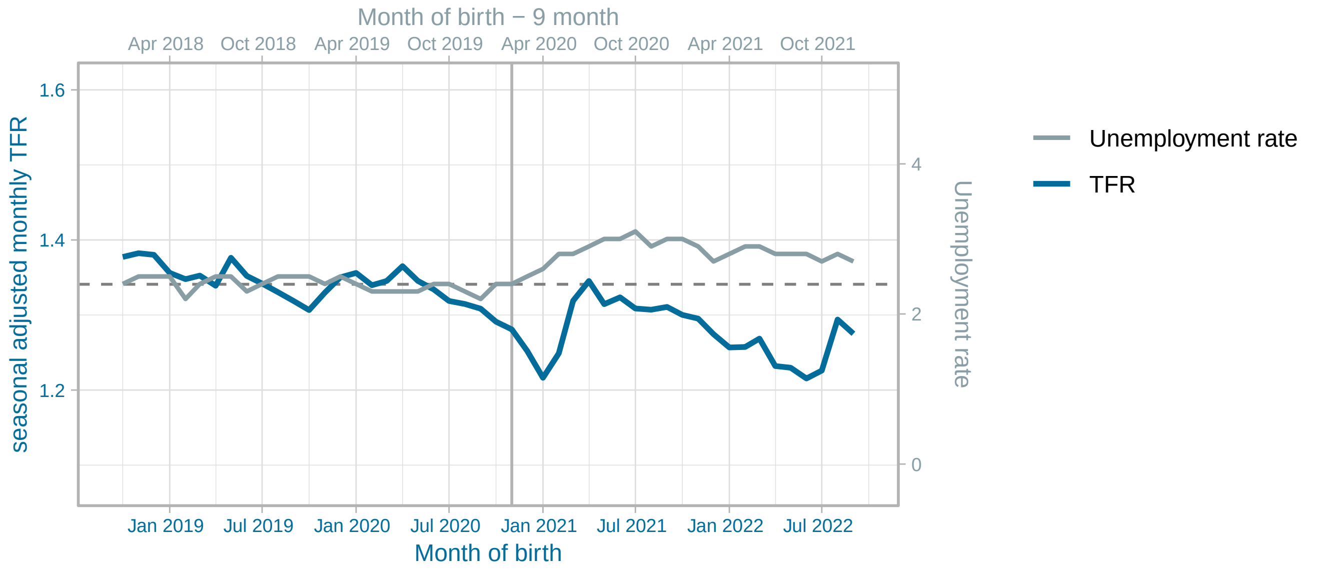

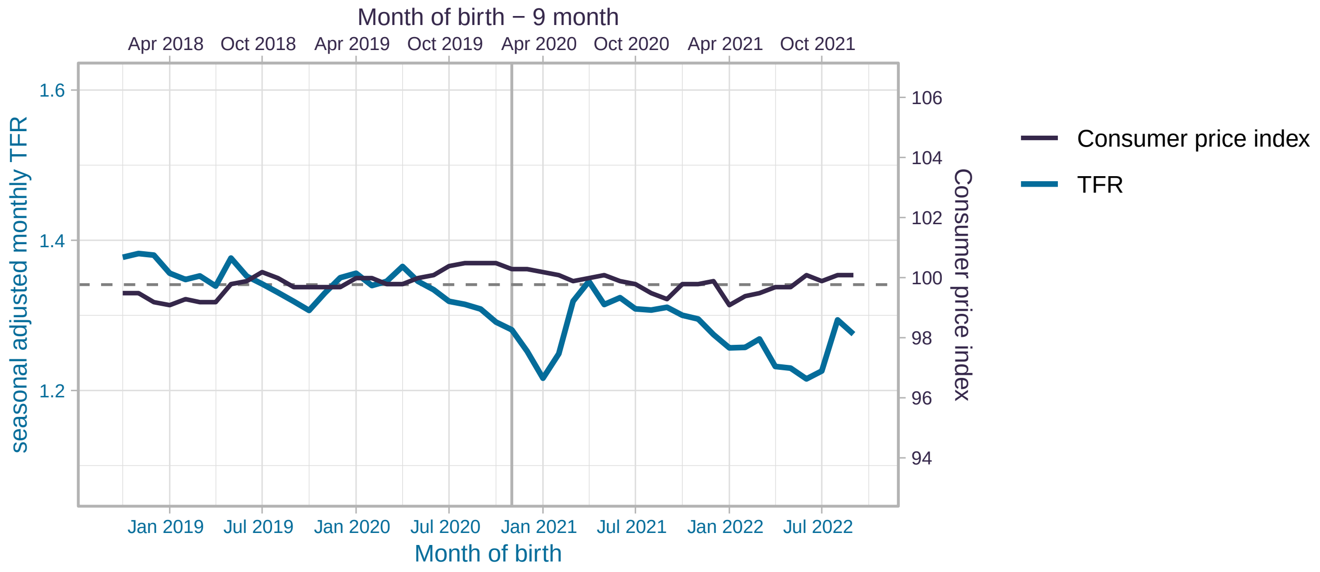

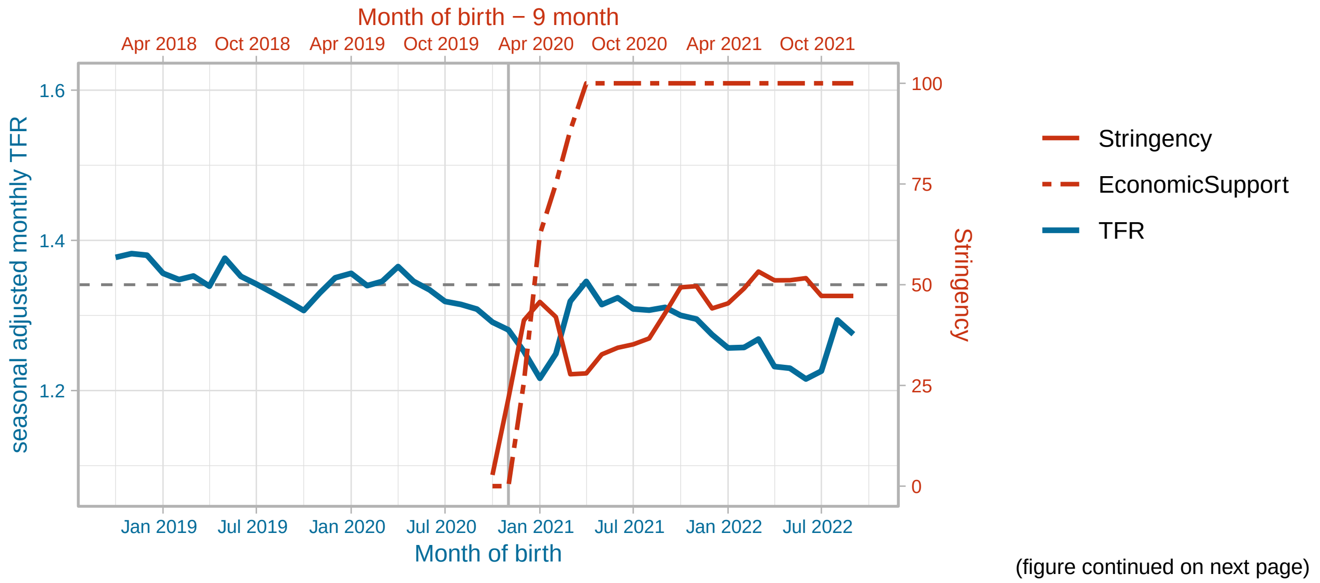

(figure continued on next page)

Japan

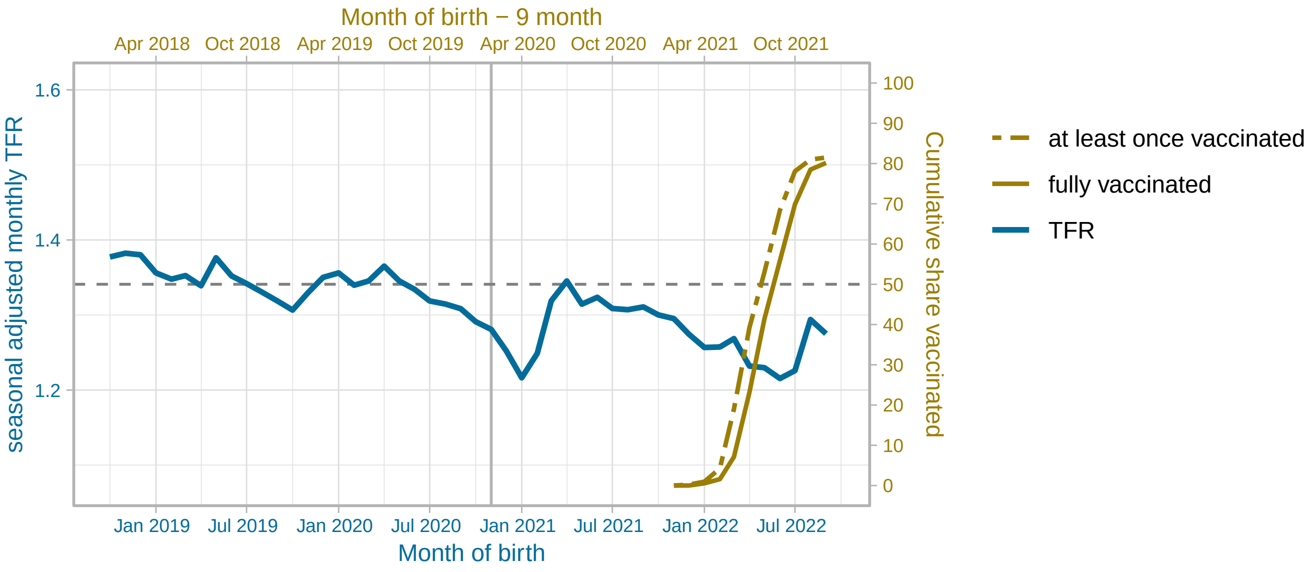

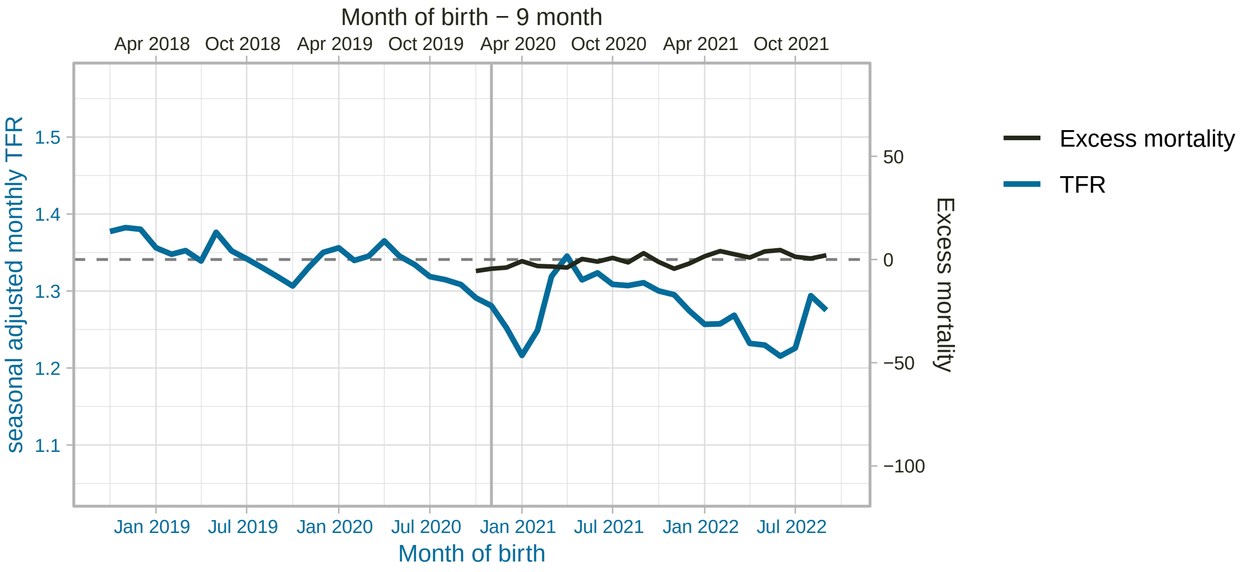

(figure continued on next page)

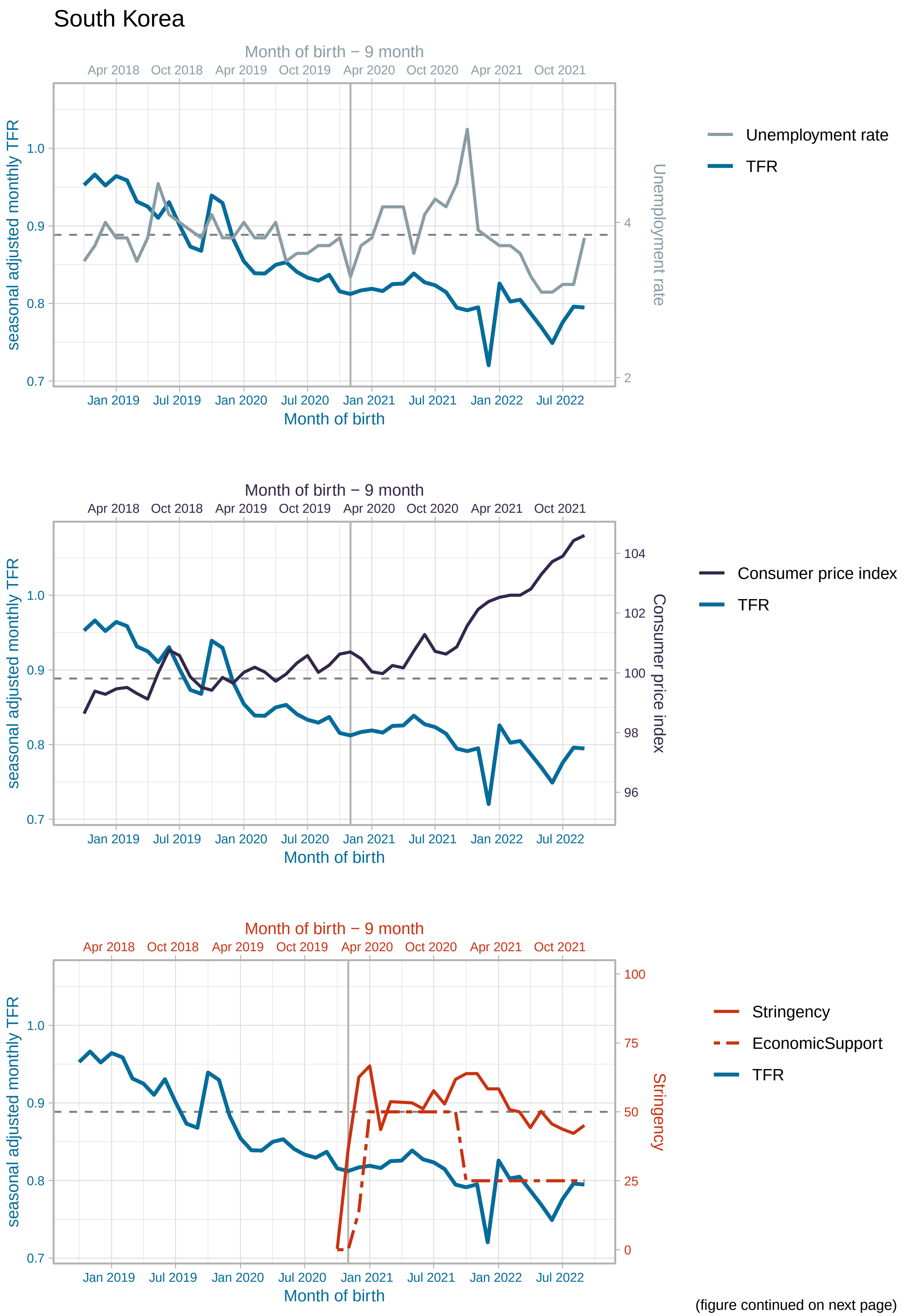

(figure continued on next page)

South Korea

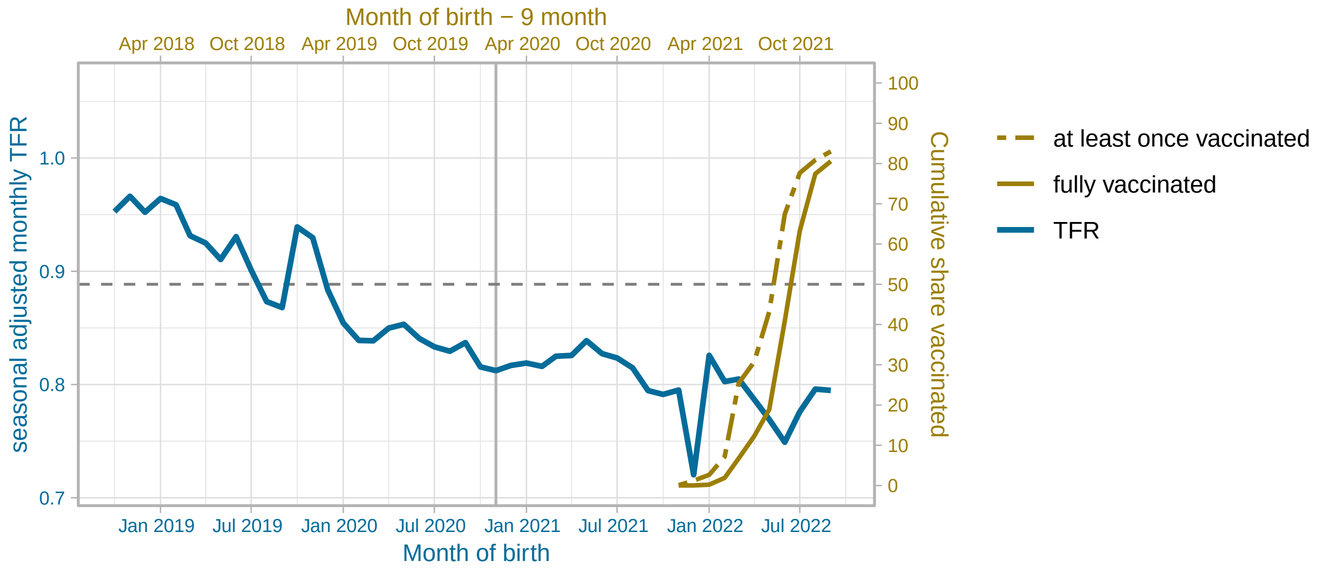

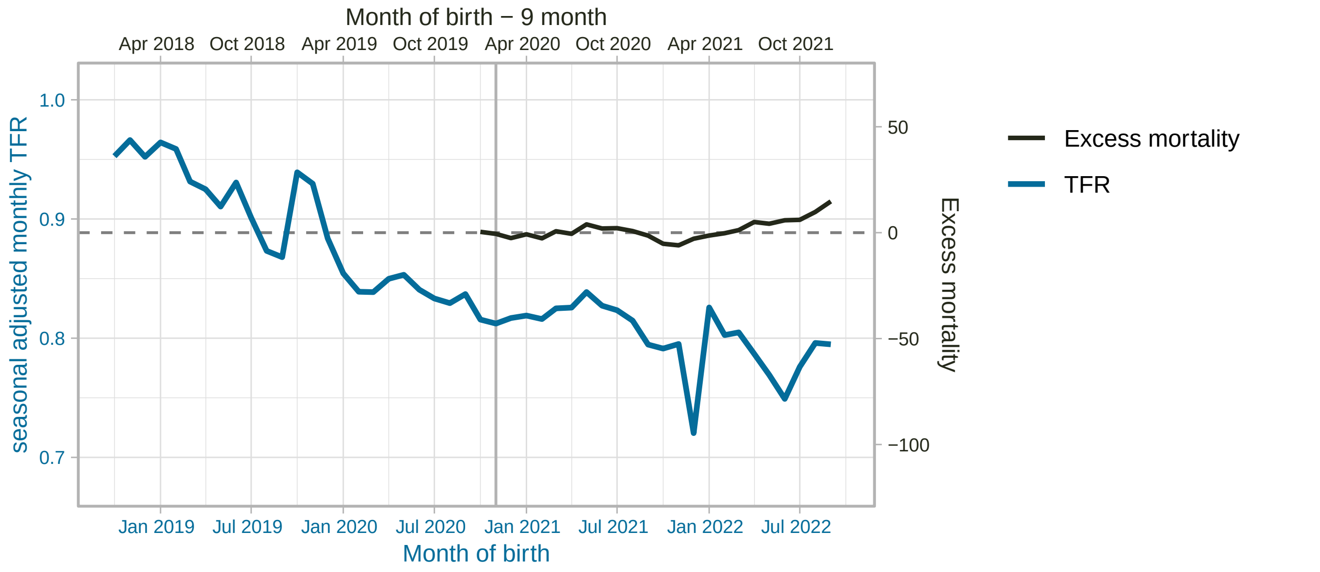

(figure continued on next page)

Latvia

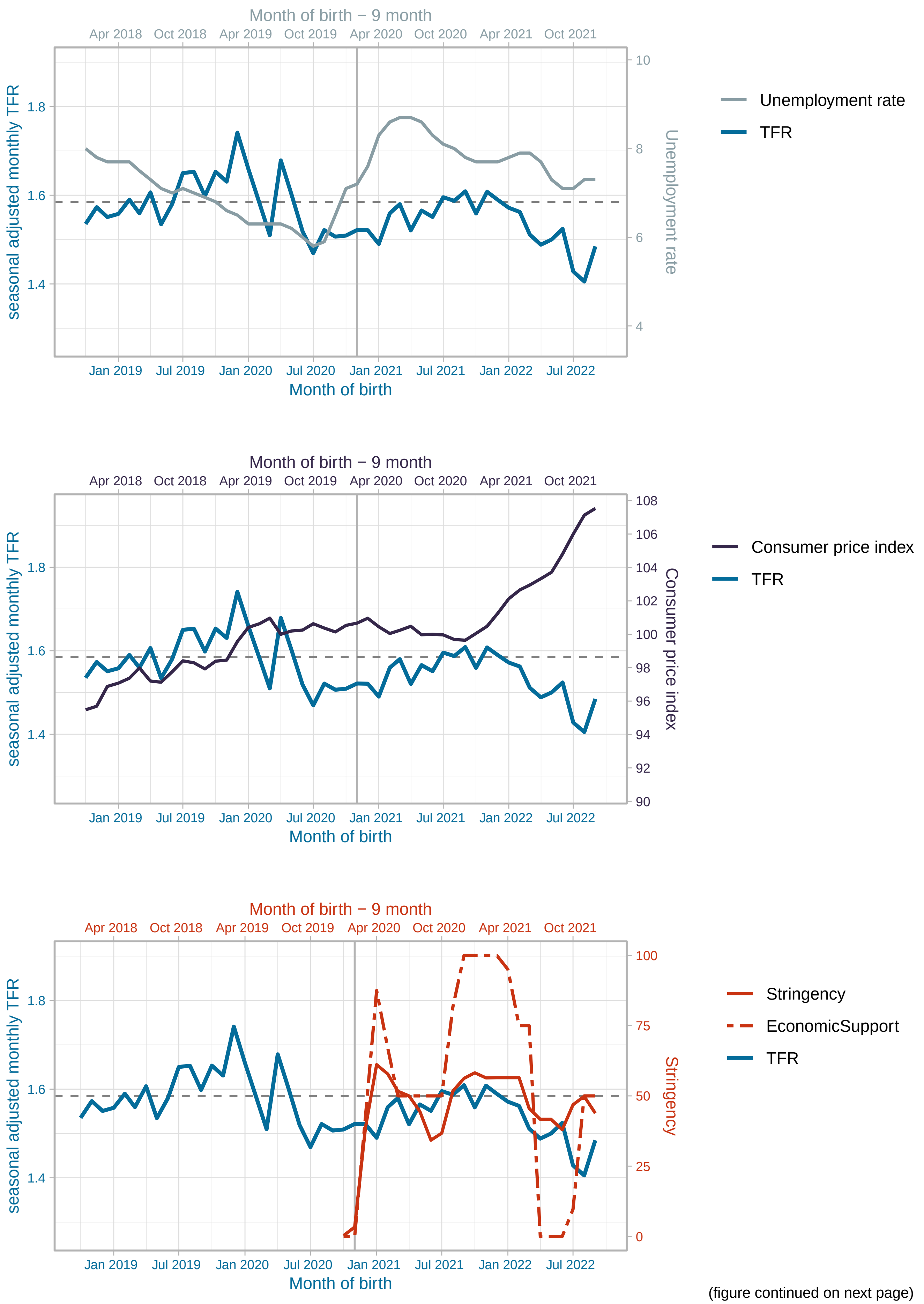

(figure continued on next page)

Latvia

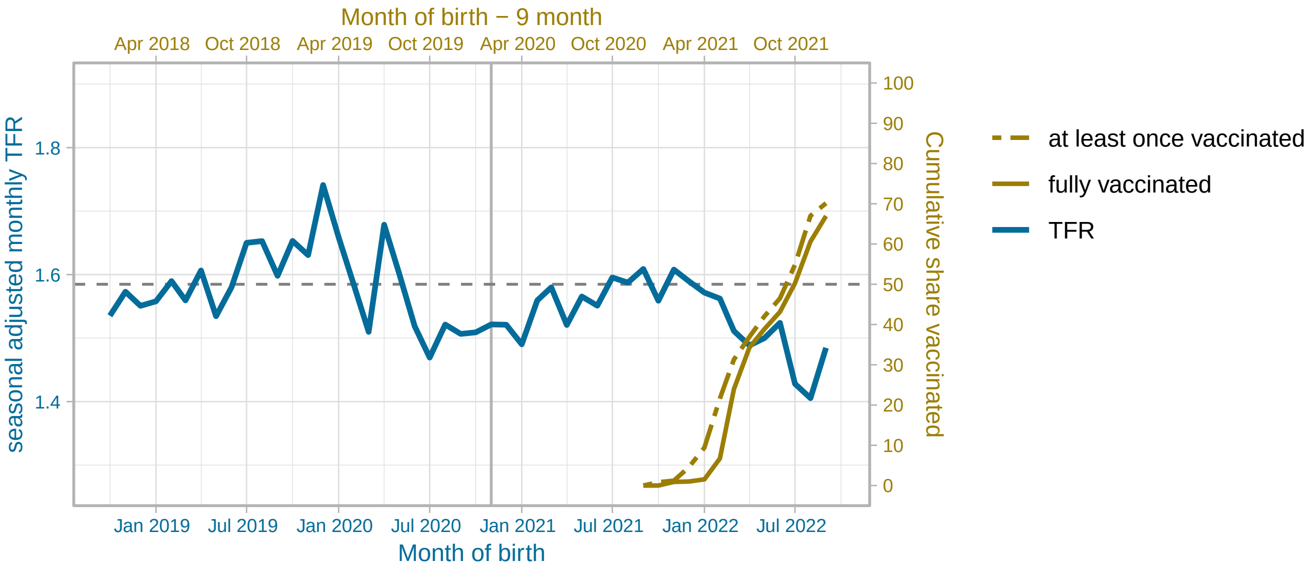

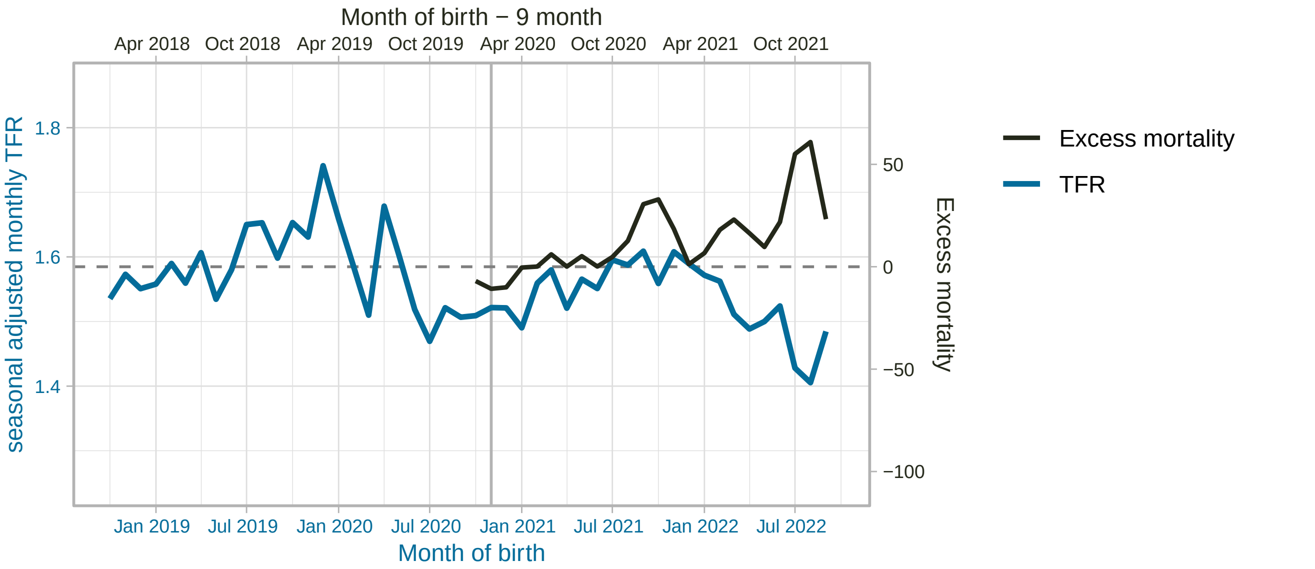

(figure continued on next page)

Netherlands

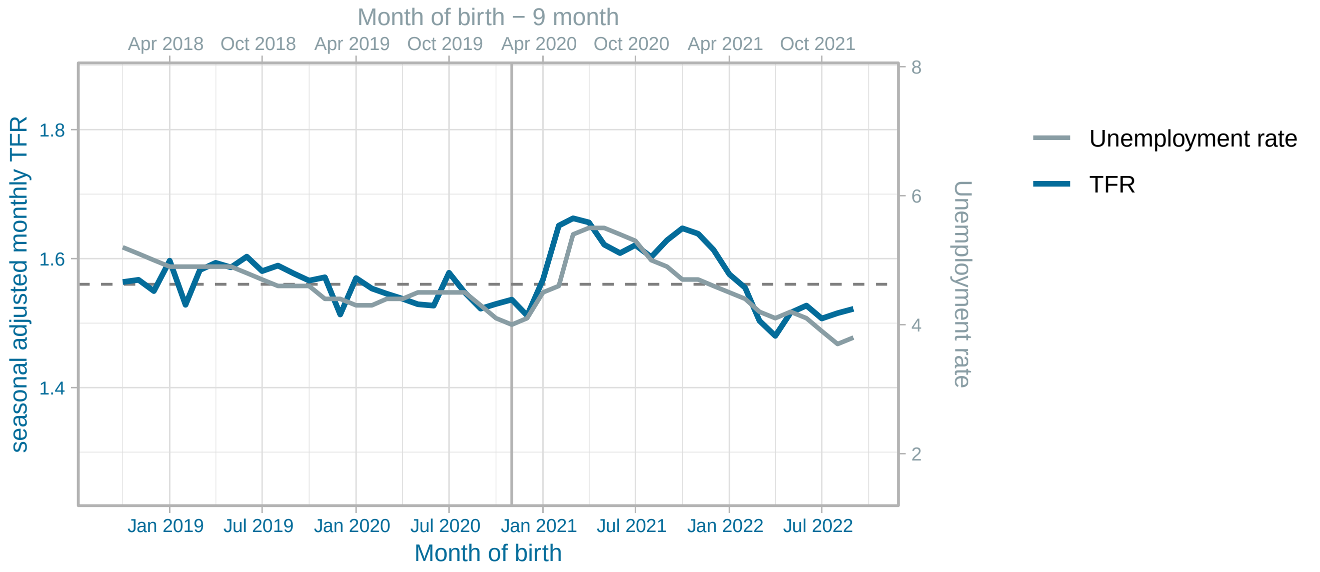

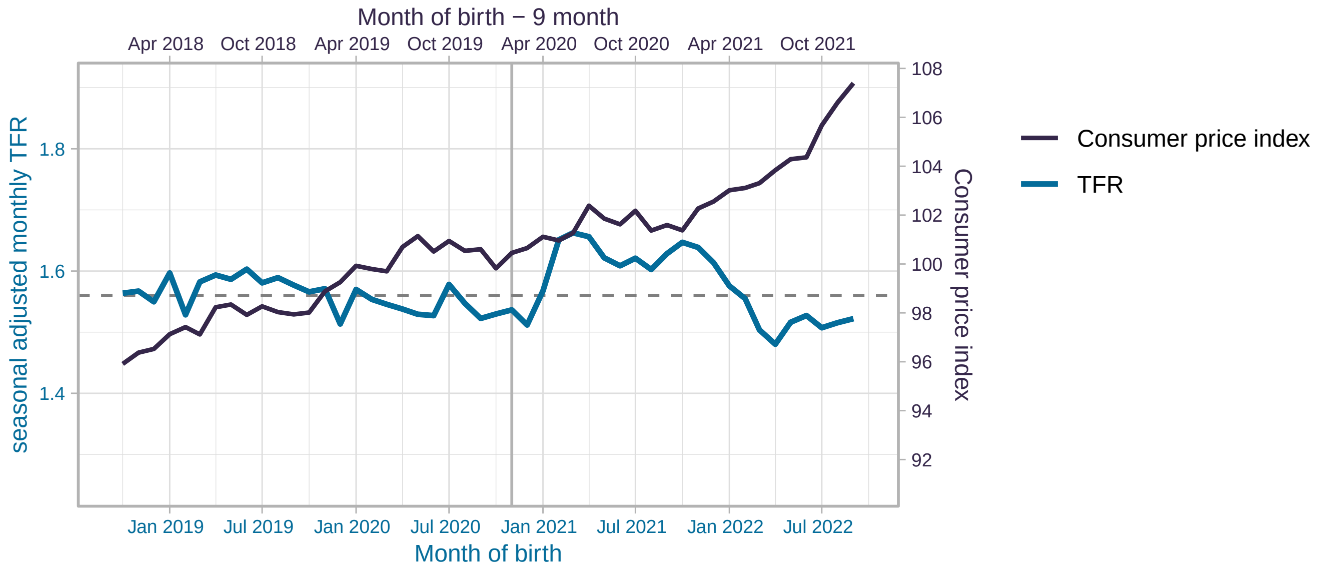

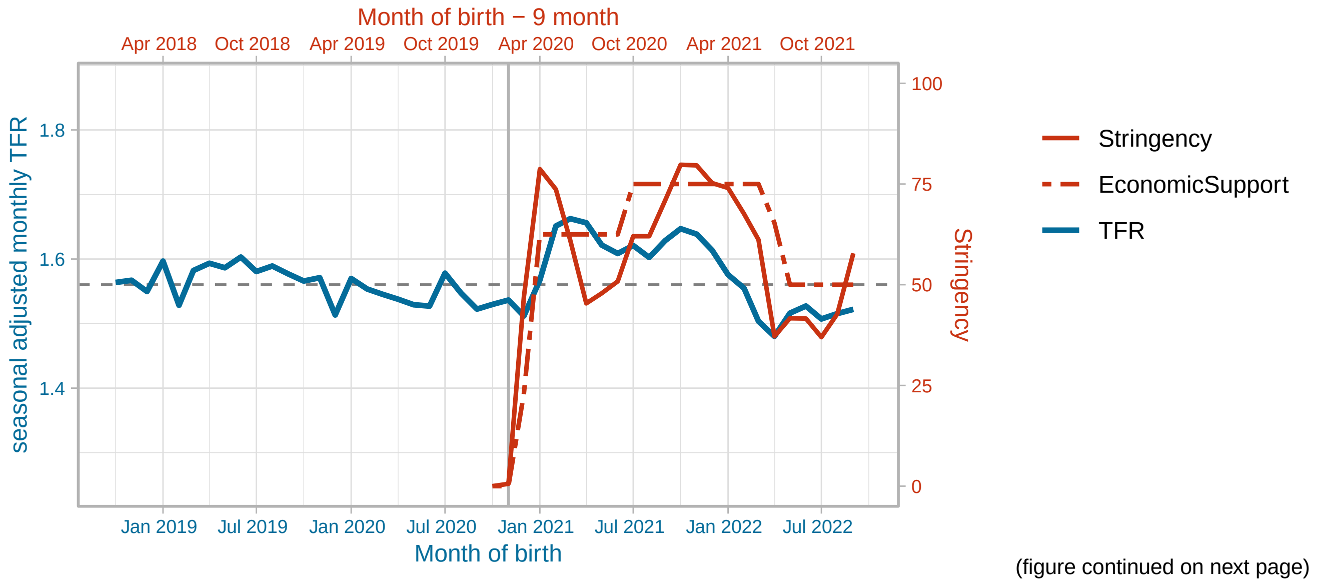

(figure continued on next page)

Netherlands

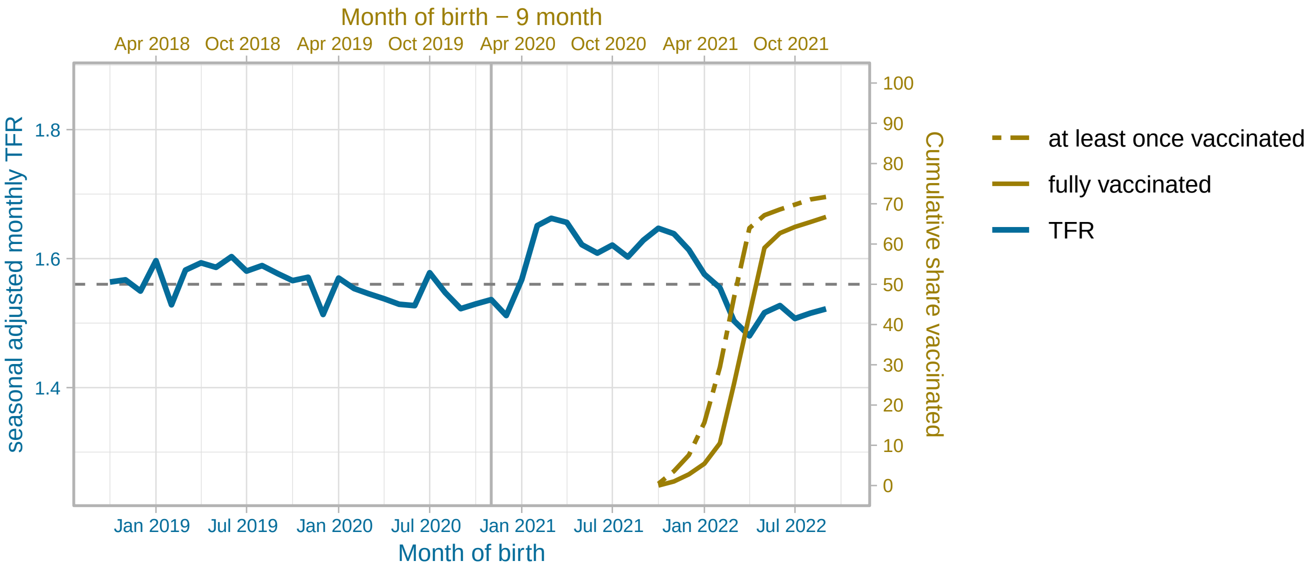

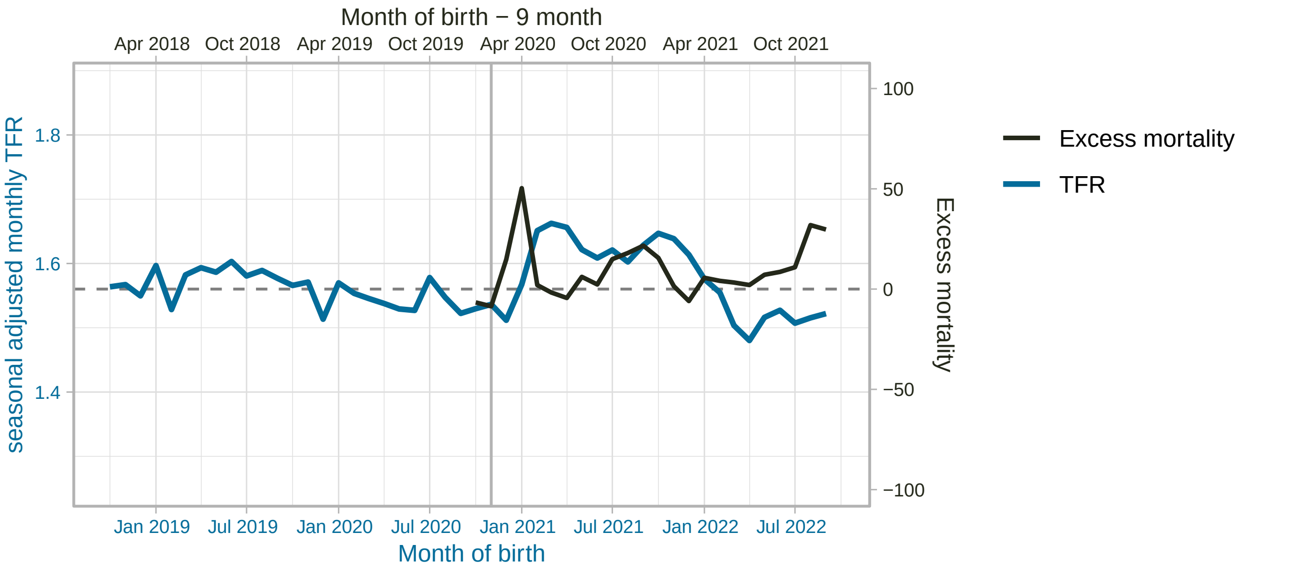

(figure continued on next page)

Norway

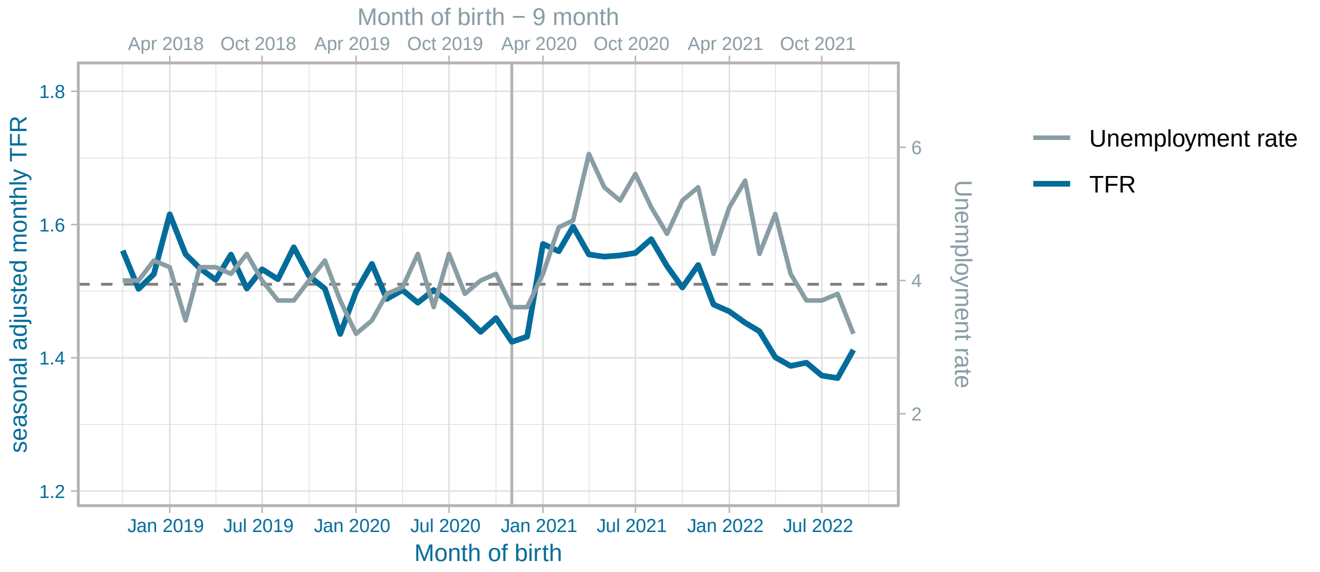

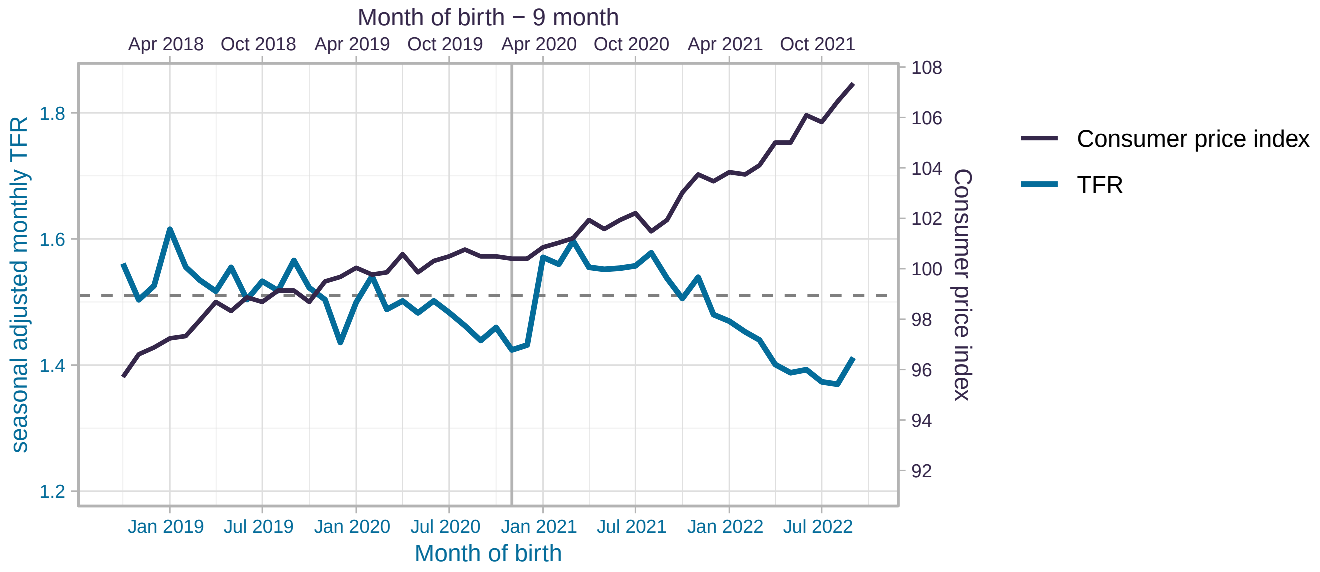

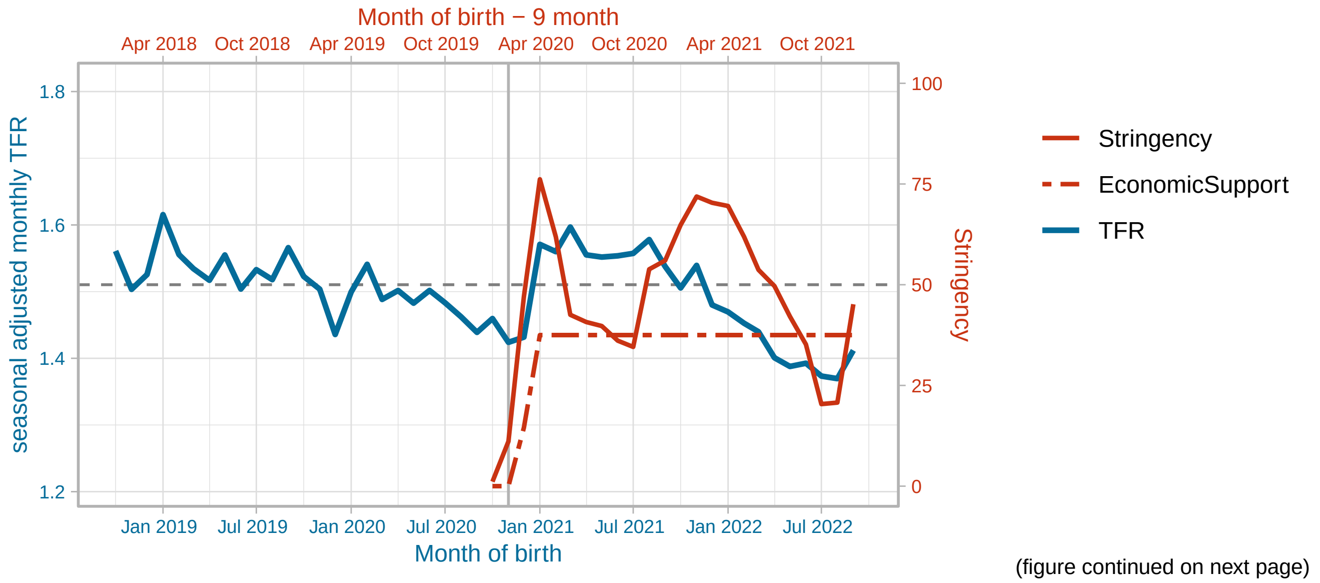

(figure continued on next page)

Norway

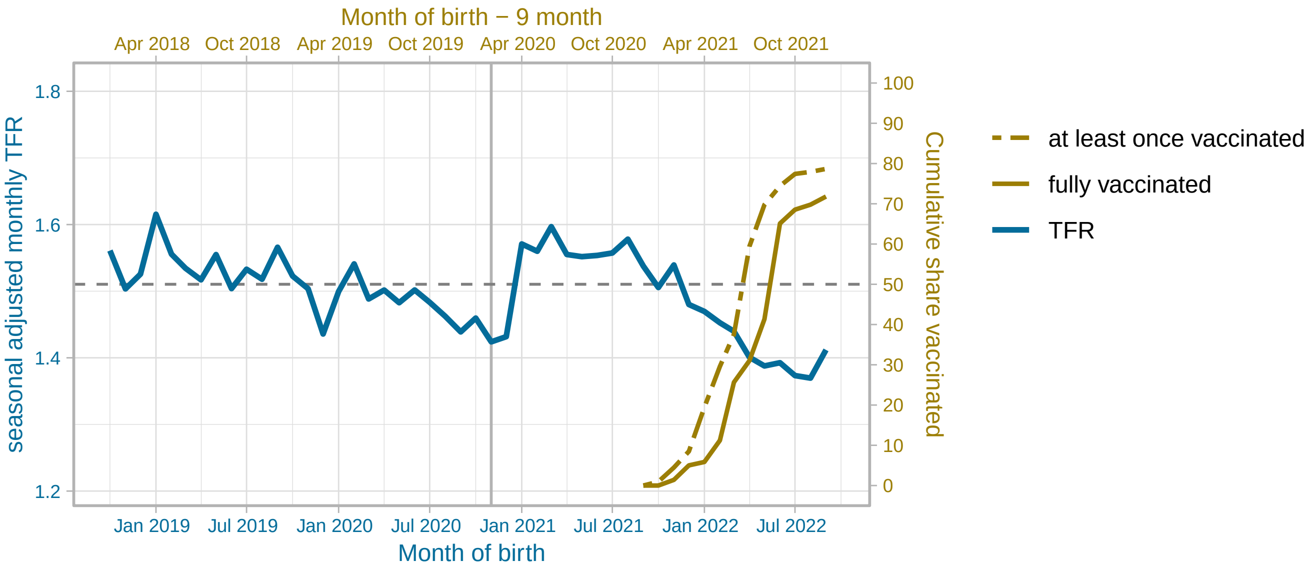

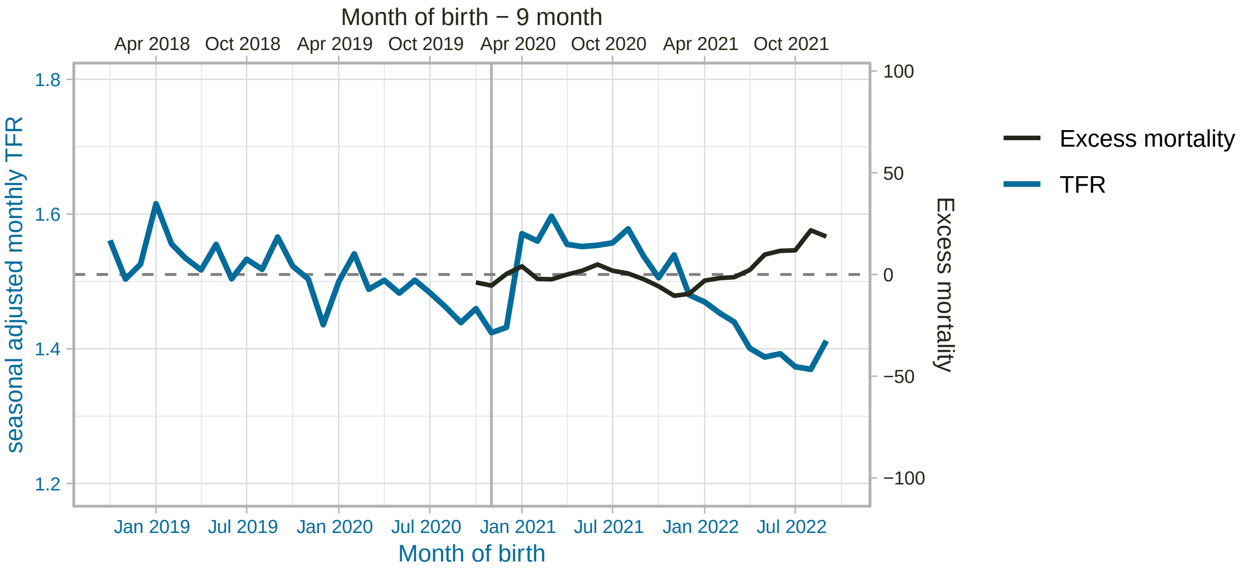

(figure continued on next page)

Poland

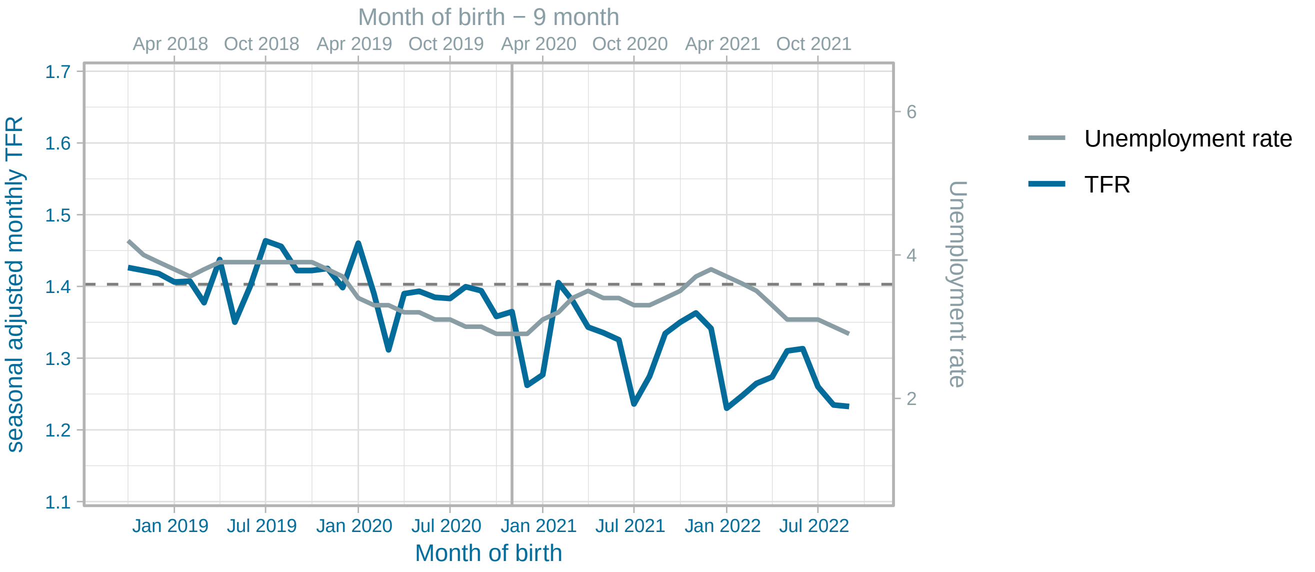

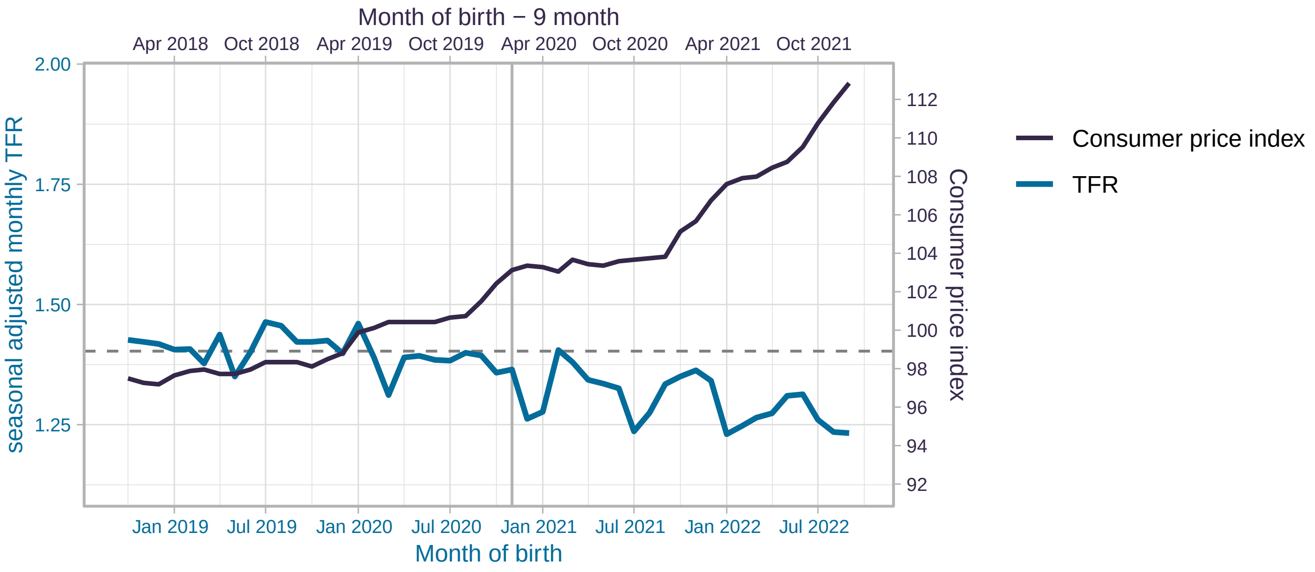

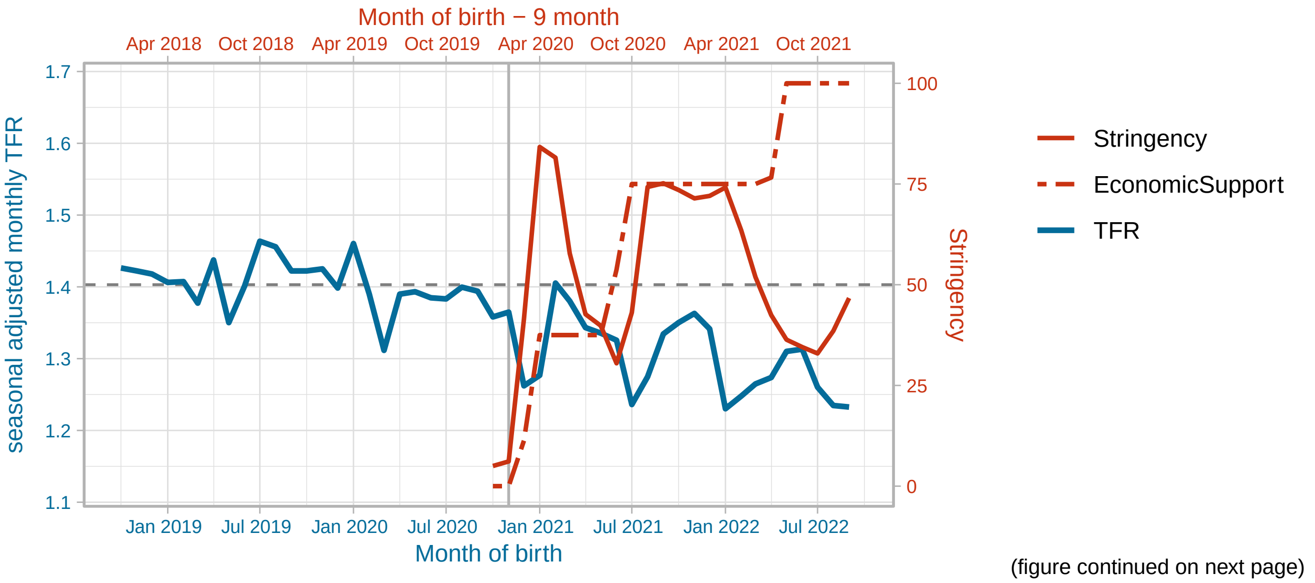

(figure continued on next page)

Poland

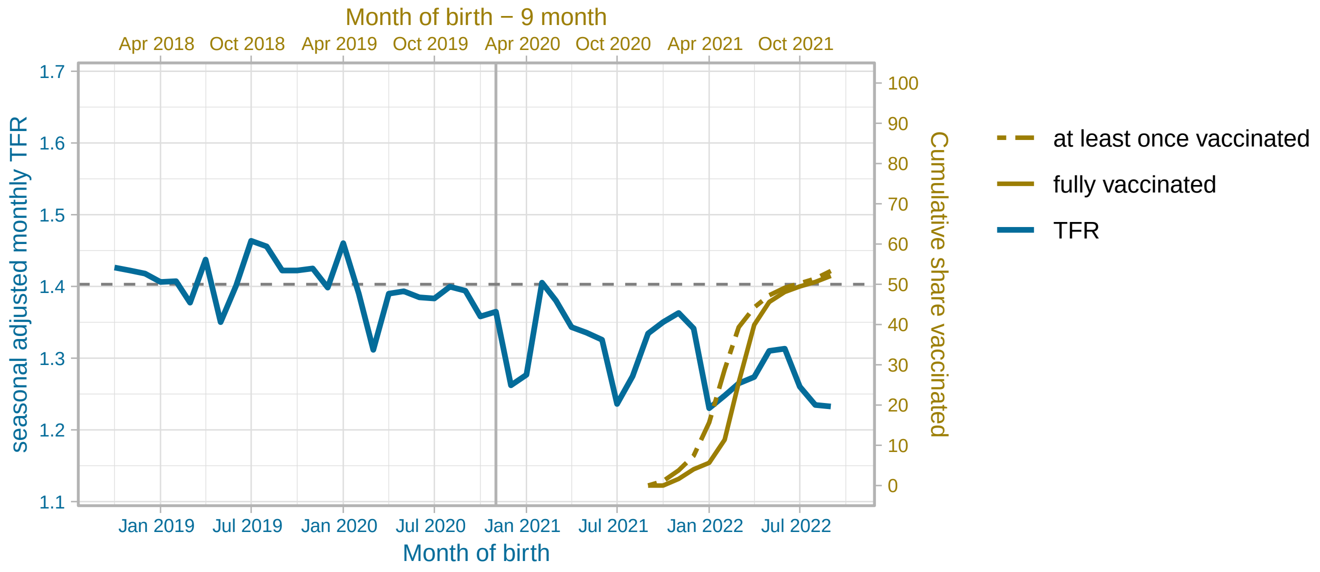

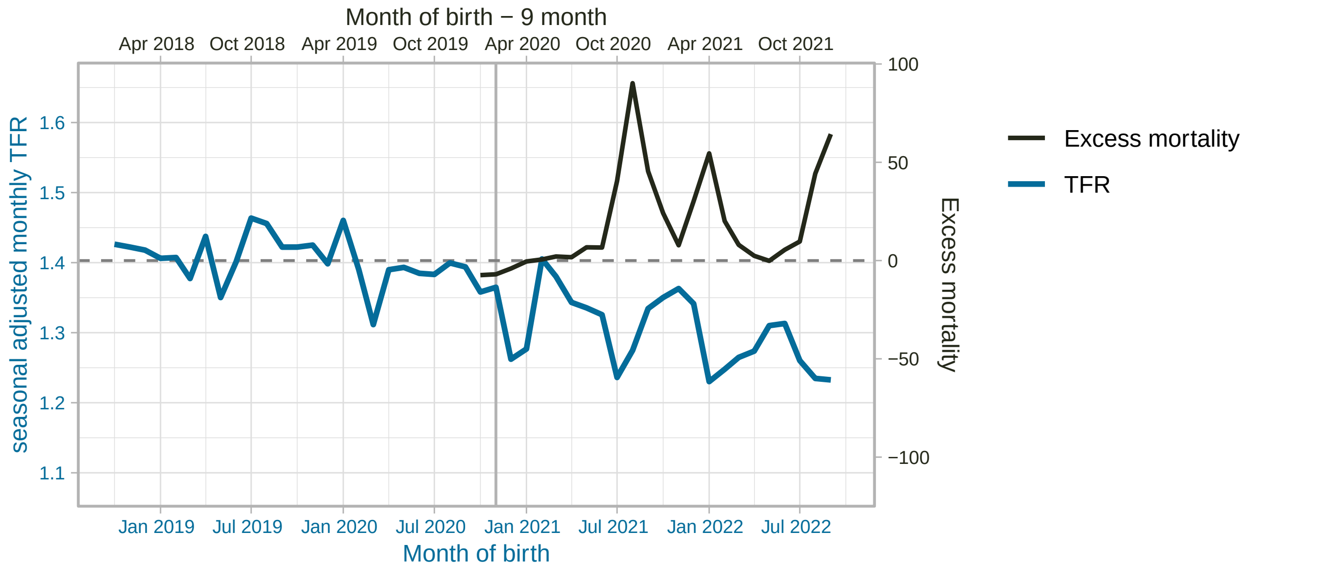

(figure continued on next page)

Portugal

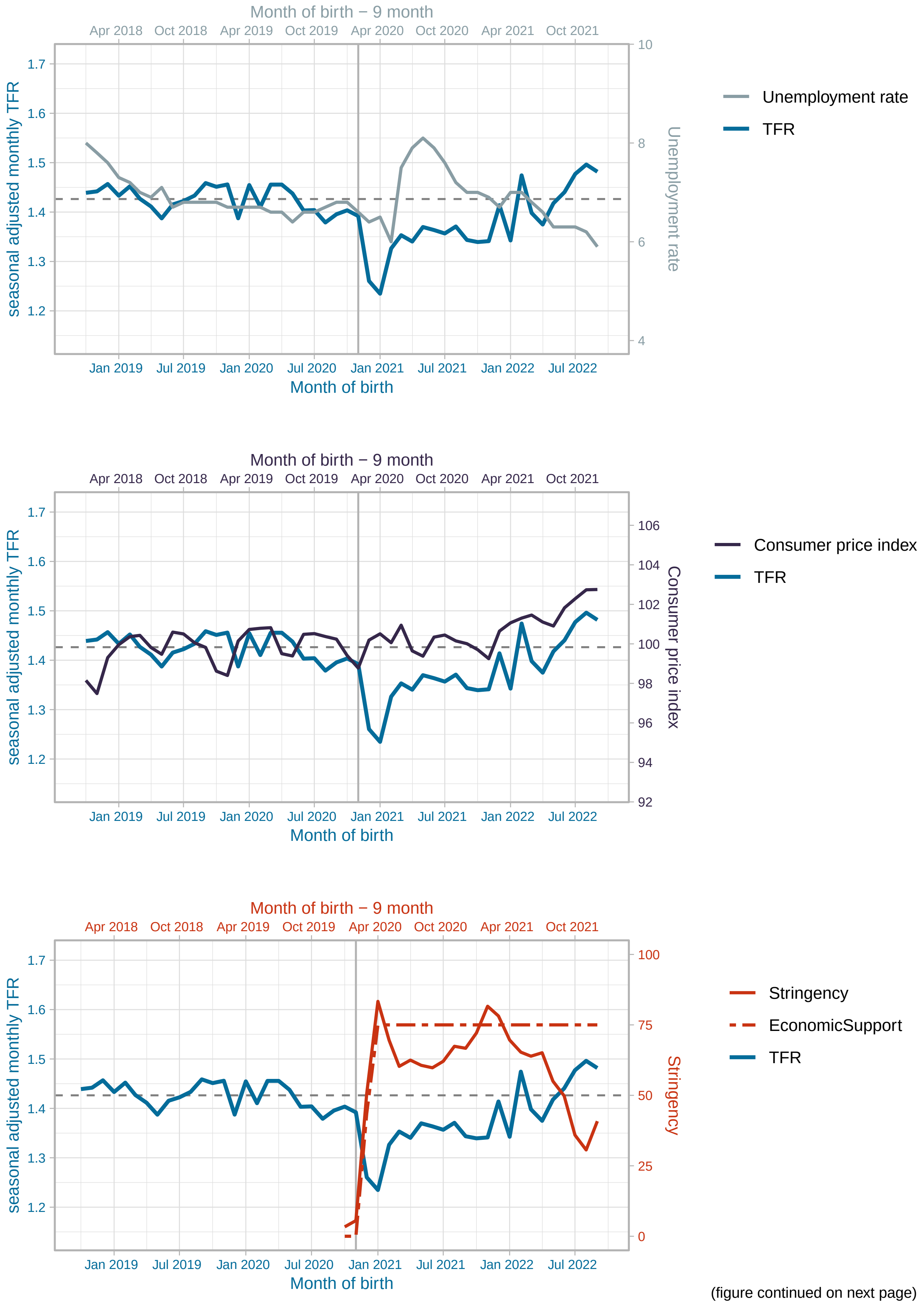

(figure continued on next page)

Portugal

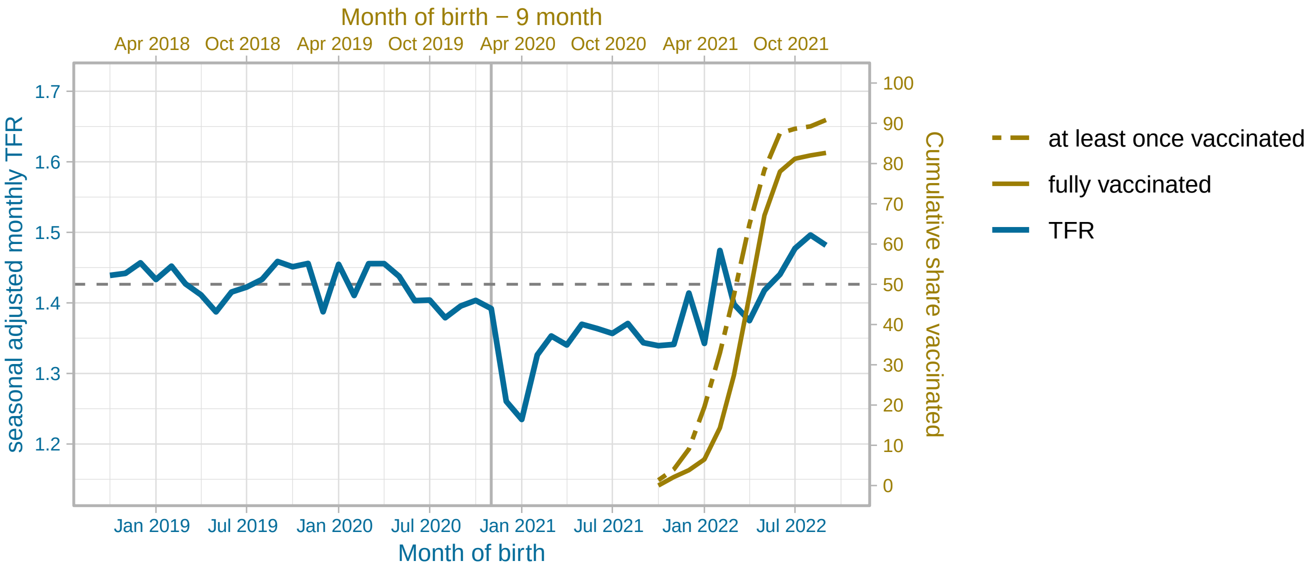

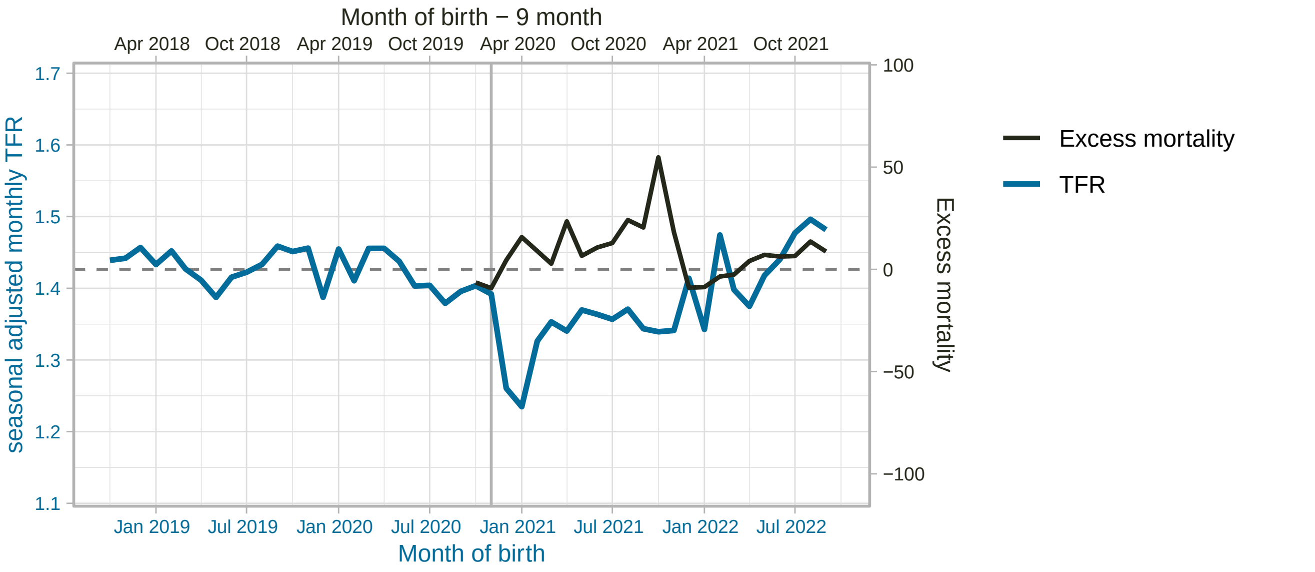

(figure continued on next page)

Slovenia

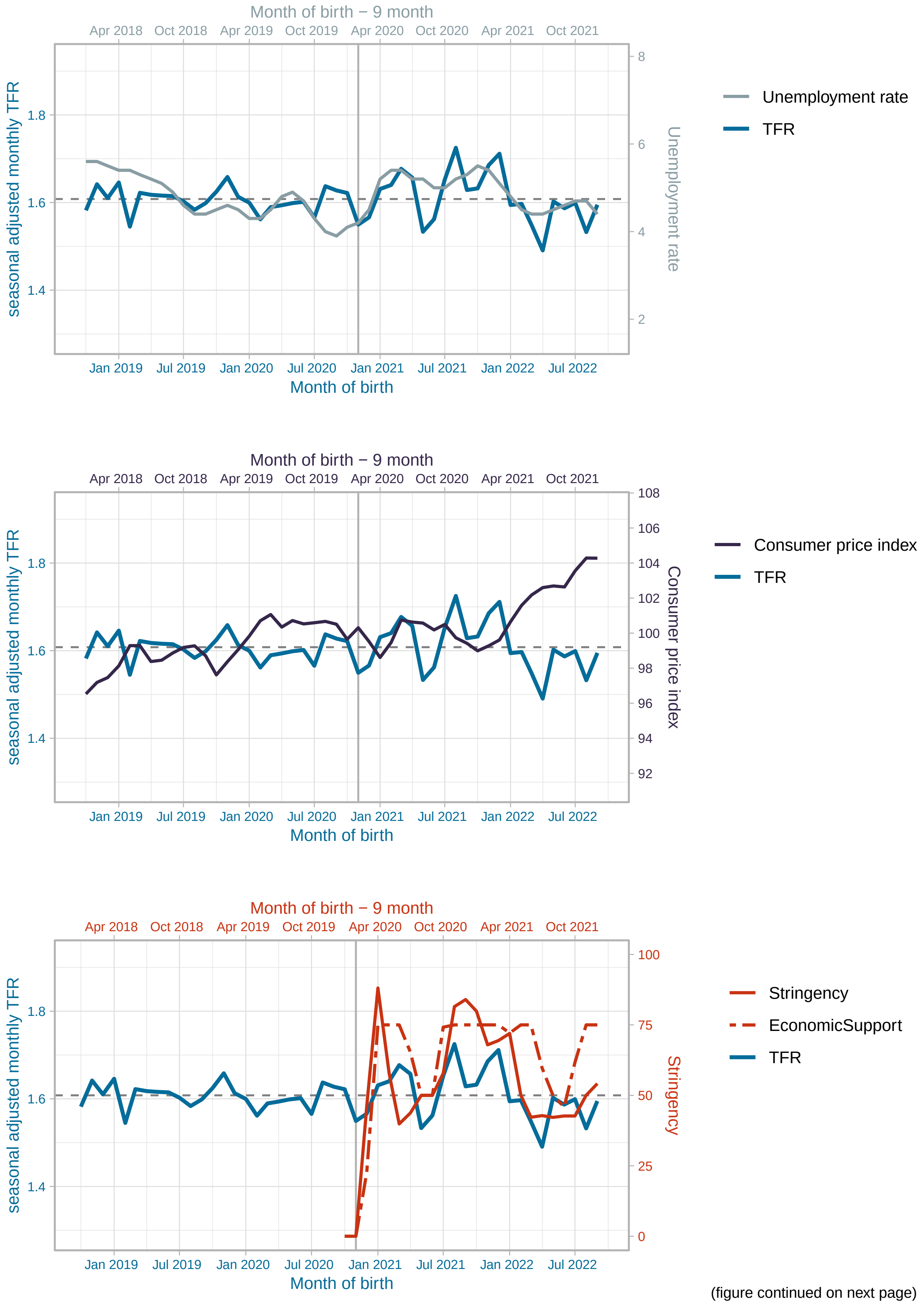

(figure continued on next page)

Slovenia

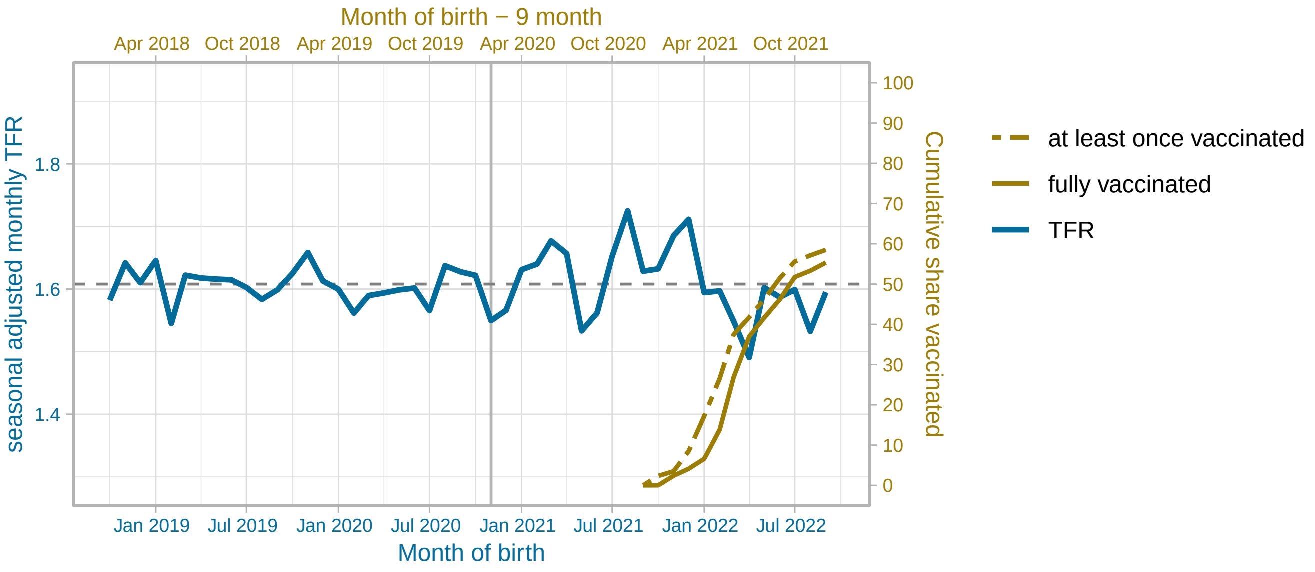

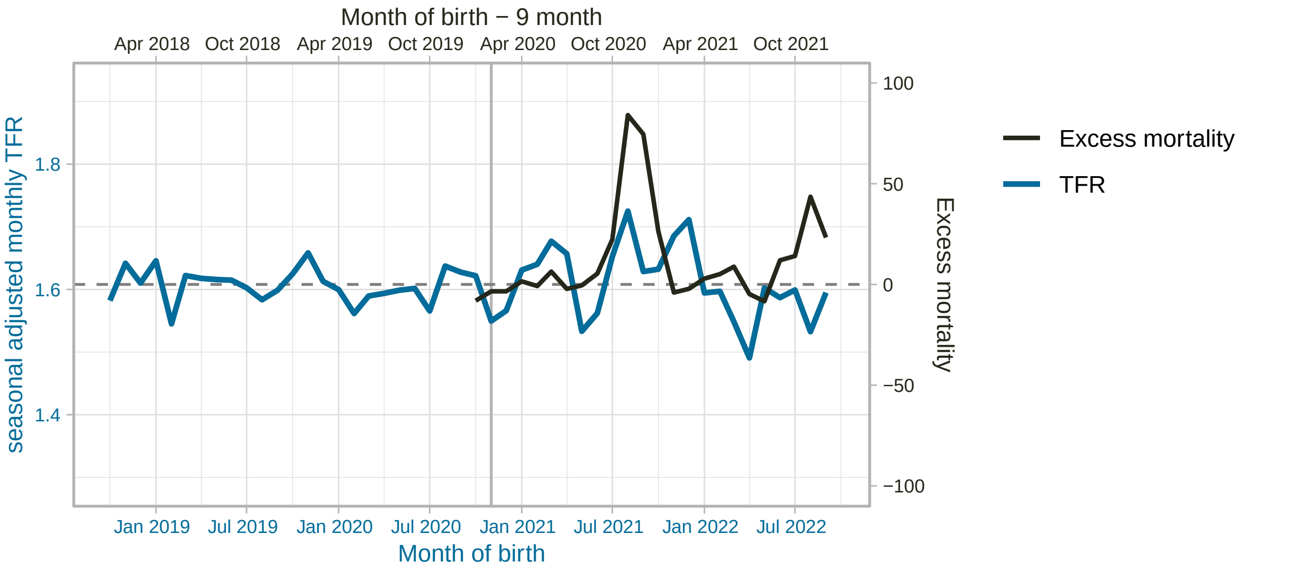

(figure continued on next page)

Sweden

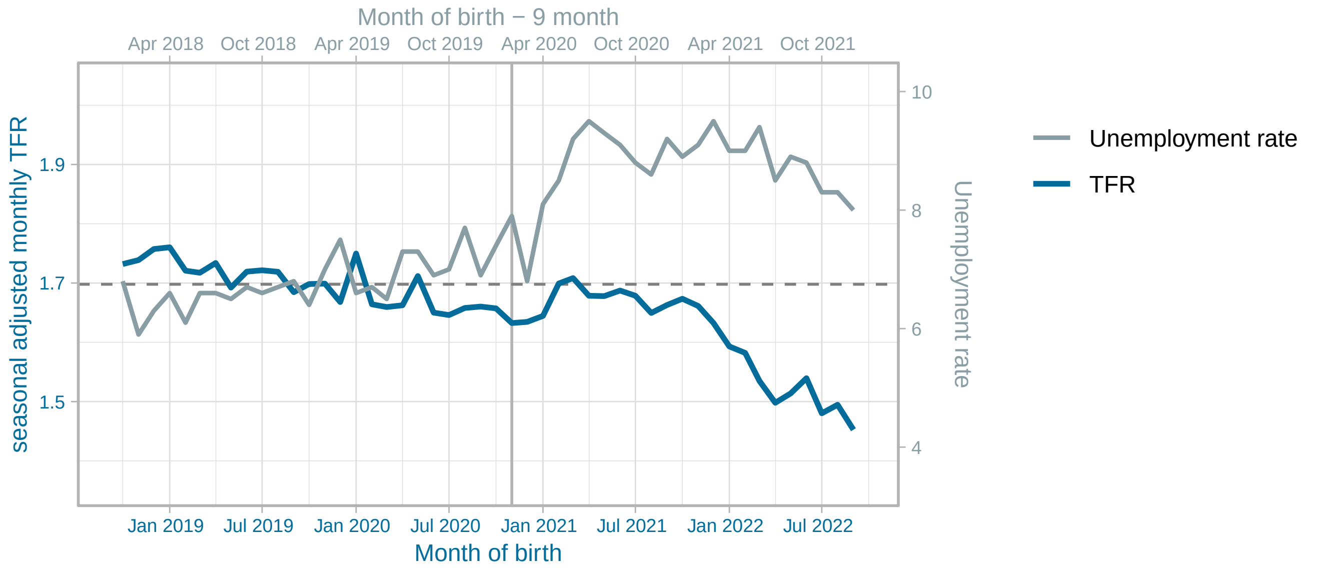

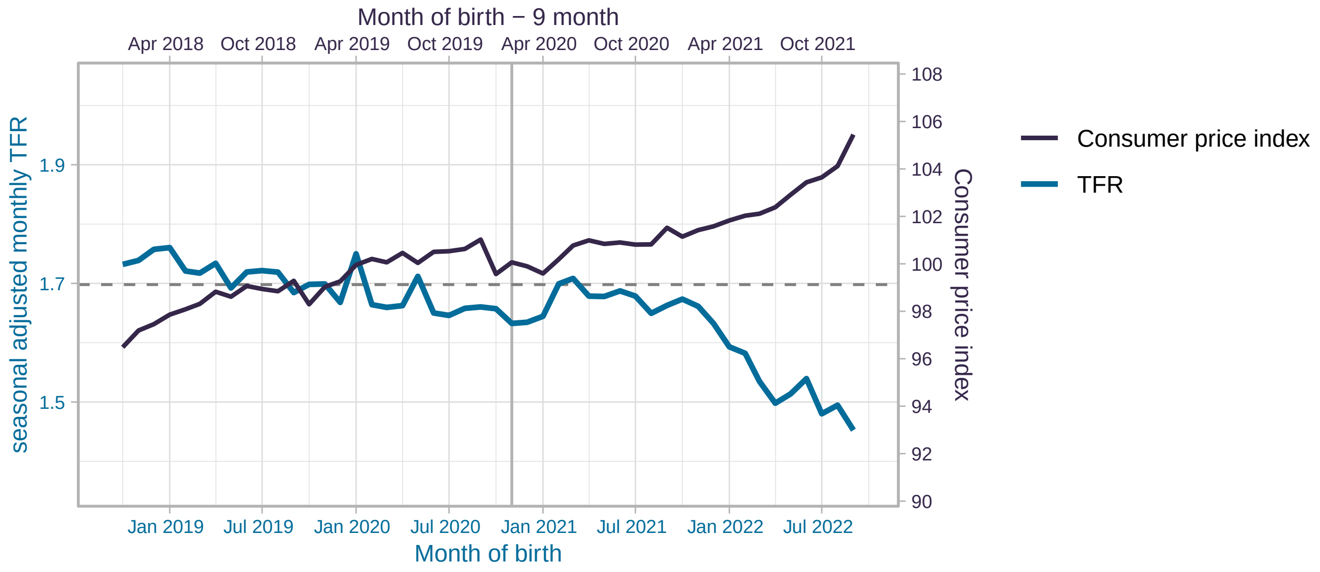

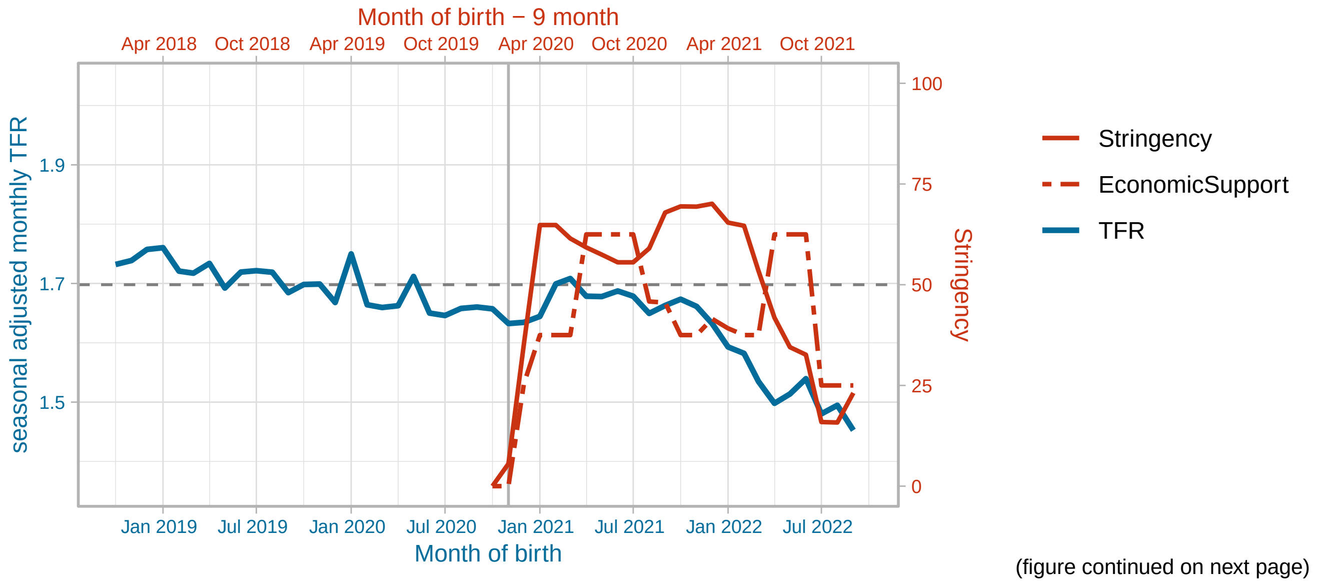

(figure continued on next page)

Sweden

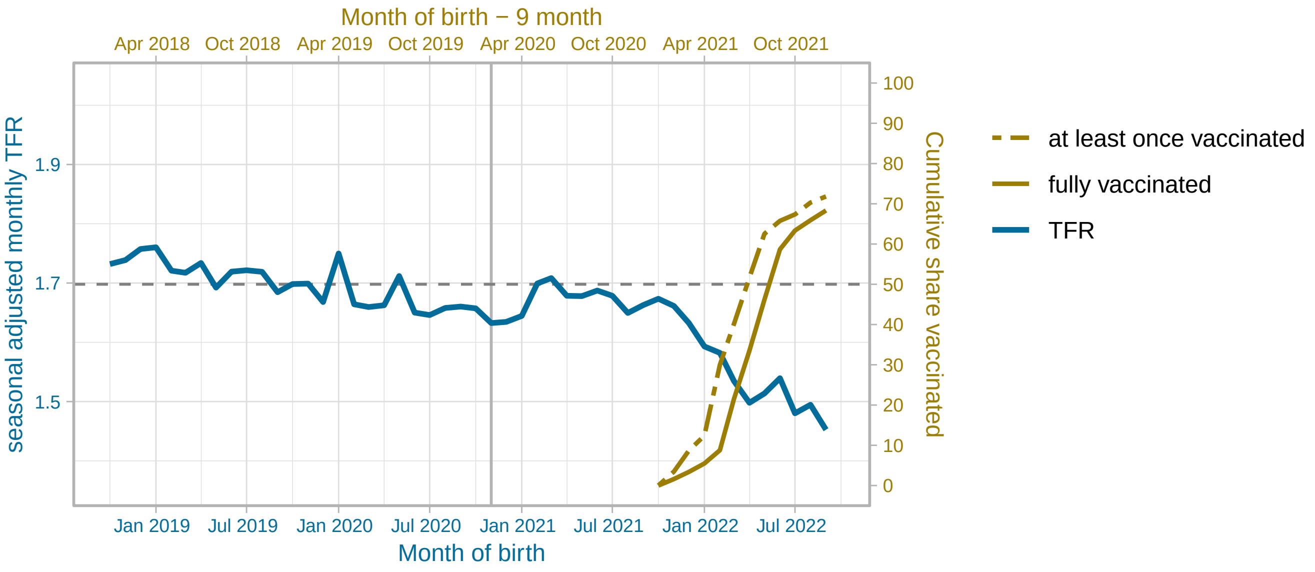

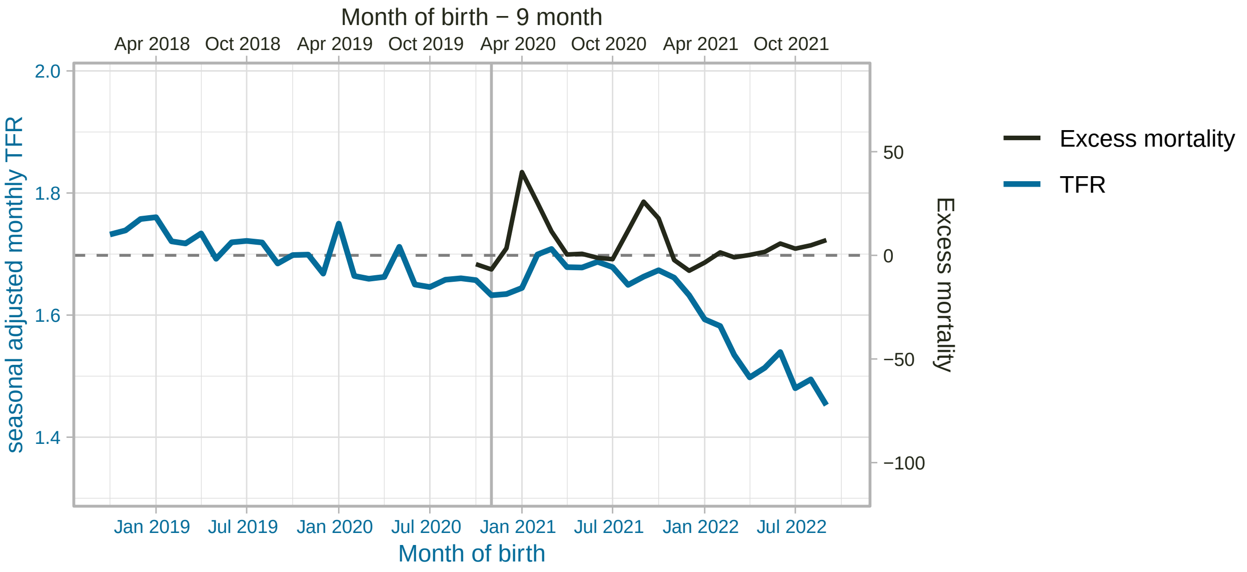

(figure continued on next page)

United States

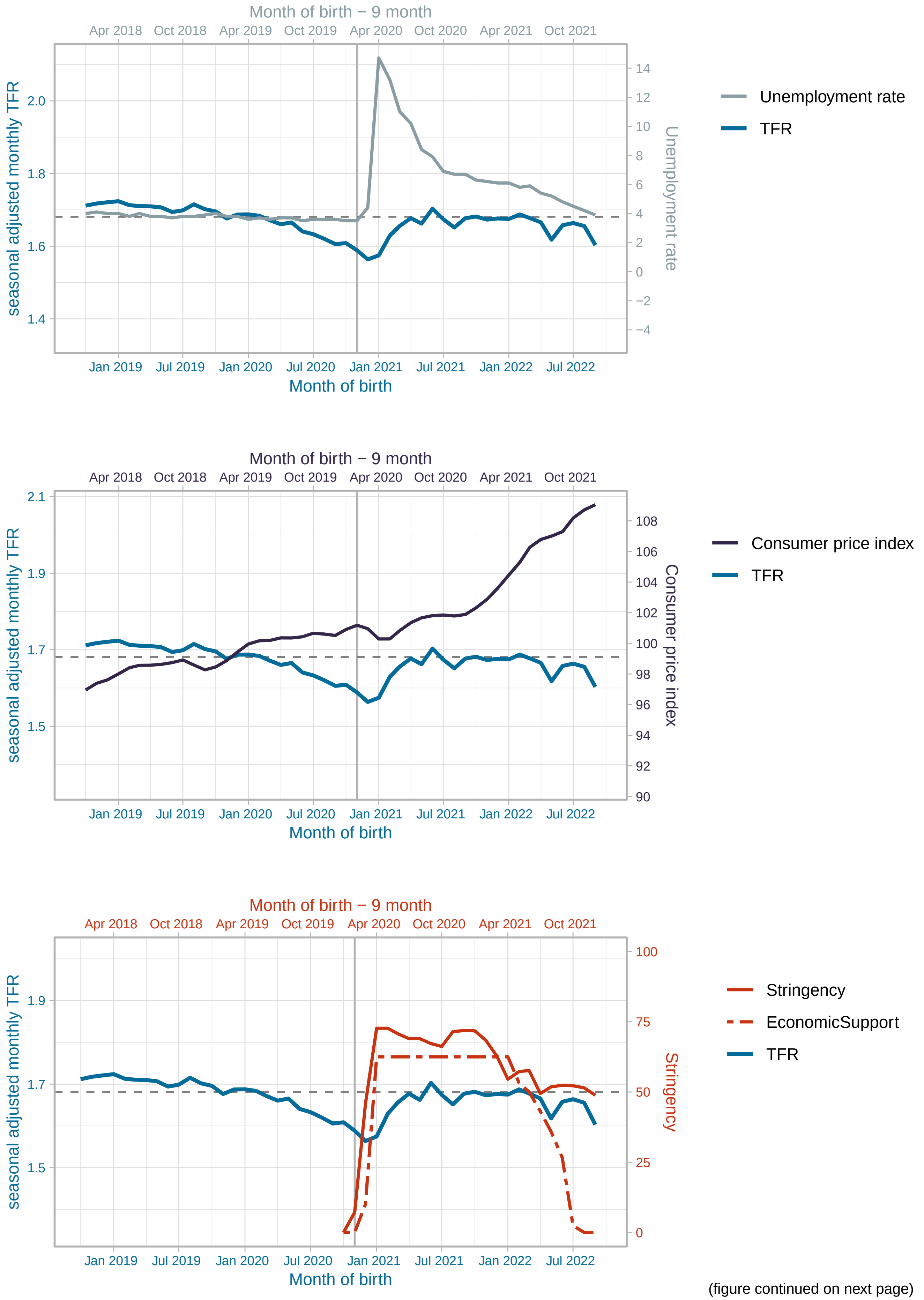

(figure continued on next page)

United States

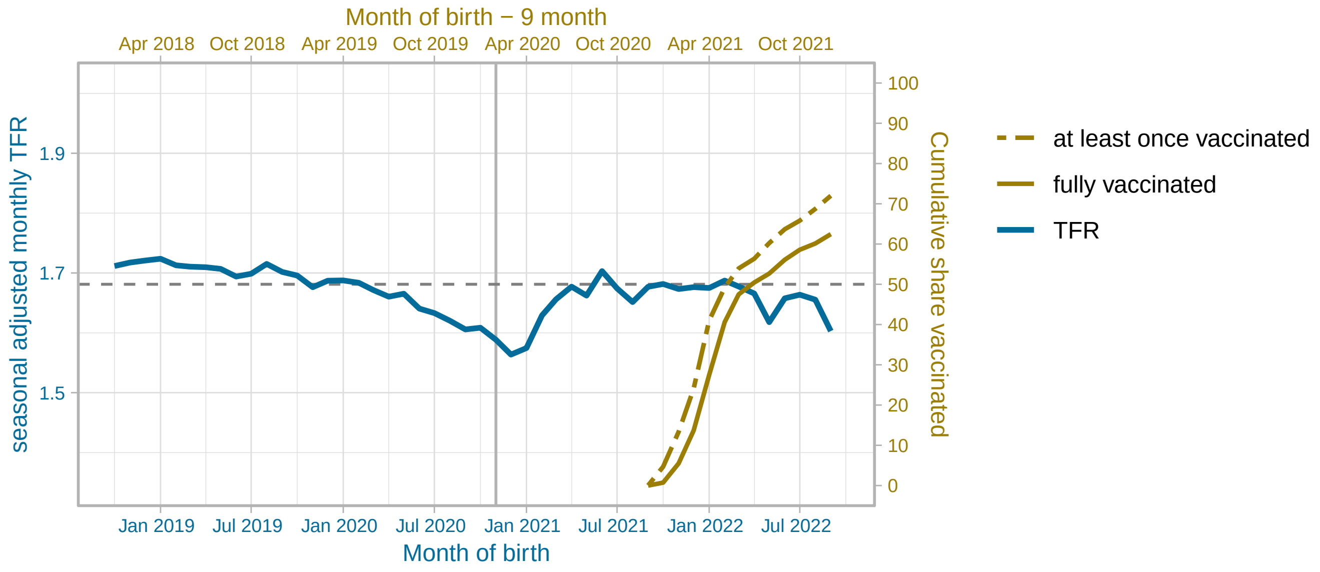

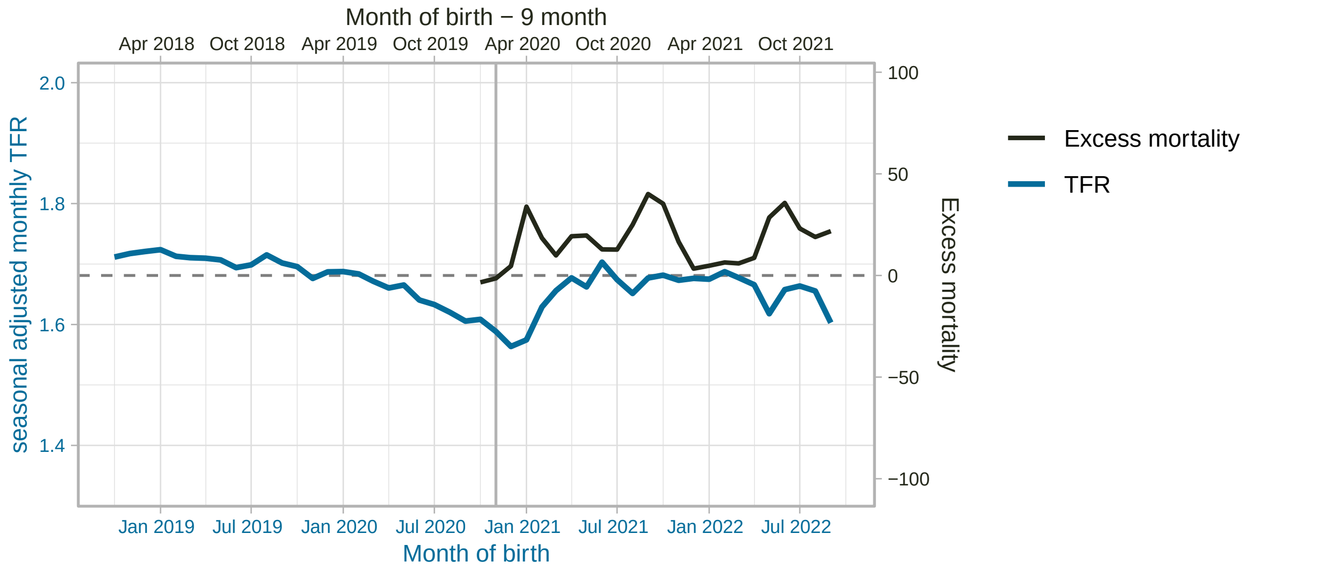

Source: Own computations based on Human Fertility Database (HFD, 2023), OECD (2023a,c,d), Oxford COVID−19 Government Response Tracker (Hale et al., 2021), and Our World in Data (Mathieu et al., 2020).

Supplementary Figure S3: Estimated model coefficients of separate fixed effect models of the monthly seasonally adjusted total fertility rate per 100 women for conceptions occurring in three different pandemic periods, respectively.

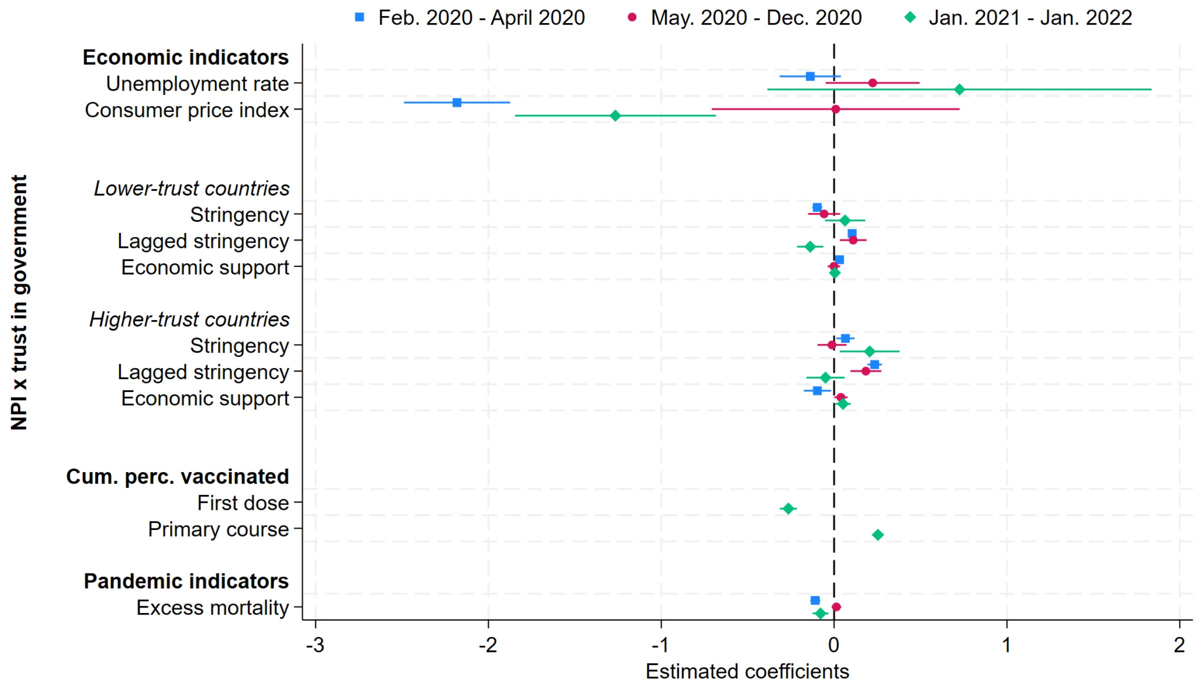

Note: The regression models additionally include country fixed effects.
NPI … non-pharmaceutical policy interventions
